# Development and Characterization of New Single Nucleotide Polymorphism Markers from Expressed Sequence Tags in Common Carp (*Cyprinus carpio*)

**DOI:** 10.3390/ijms13067343

**Published:** 2012-06-14

**Authors:** Chuankun Zhu, Lei Cheng, Jingou Tong, Xiaomu Yu

**Affiliations:** 1 State Key Laboratory of Freshwater Ecology and Biotechnology, Institute of Hydrobiology, Chinese Academy of Sciences, Wuhan 430072, China; E-Mails: zhuchuankun@ihb.ac.cn (C.Z.); mailchenglei@gmail.com (L.C.); xmyu@ihb.ac.cn (X.Y.); 2 Graduate School of Chinese Academy of Sciences, Beijing 100039, China

**Keywords:** common carp (*Cyprinus carpio*), expressed sequence tag (EST), single nucleotide polymorphism (SNP), validation, genetic variations

## Abstract

The common carp (*Cyprinus carpio*) is an important aquaculture fish worldwide but only limited single nucleotide polymorphism (SNP) markers are characterized from expressed sequence tags (ESTs) in this species. In this study, 1487 putative SNPs were bioinformatically mined from 14,066 online ESTs mainly from the European common carp, with the occurrence rate of about one SNP every 173 bp. One hundred and twenty-one of these SNPs were selected for validation using PCR fragment sequencing, and 48 out of 81 primers could amplify the expected fragments in the Chinese common carp genome. Only 26 (21.5%) putative SNPs were validated, however, 508 new SNPs and 68 indels were identified. The ratios of transitions to transversions were 1.77 for exon SNPs and 1.05 for intron SNPs. All the 23 SNPs selected for population tests were polymorphic, with the observed heterozygosity (Ho) ranging from 0.053 to 0.526 (mean 0.262), polymorphism information content (PIC) from 0.095 to 0.357 (mean 0.246), and 21 SNPs were in Hardy–Weinberg equilibrium. These results suggest that different common carp populations with geographic isolation have significant genetic variation at the SNP level, and these new EST-SNP markers are readily available for genetics and breeding studies in common carp.

## 1. Introduction

The common carp (*Cyprinus carpio*) is an important freshwater species for aquaculture and has been domesticated for *ca*. 4000 years [1]. Annual production of common carp has been increasing for 30 years, and recent production reached 3.3 million tons [2]. Numerous microsatellite markers [3–5] have been developed in common carp in response to the demands of genetic and breeding studies. Compared with microsatellites, single nucleotide polymorphisms (SNPs) are more promising molecular markers in genetics, genomics and aquaculture. Because of their high abundance in the genome, hereditable stability and allele portability, and the possibility for high-throughput analysis, they may replace traditional molecular markers in genetic studies [6,7]. EST-database mining is an efficient way to obtain SNP markers and was first applied for SNP discovery in human sequences [8], EST-SNPs were also obtained in many fish species [9–12], while to date only limited EST-SNPs have been characterized [13] in common carp. In this study, discovery of a set of EST-SNPs from online common carp EST databases and validation of selected EST-SNPs in the Chinese common carp were carried out, with the aims of (1) developing novel SNP resources for future studies of genetics and breeding in common carp; (2) validating and characterizing SNPs in common carp with different genetic backgrounds for the comparison of genetic variations.

## 2. Results and Discussion

**Figure 1:**
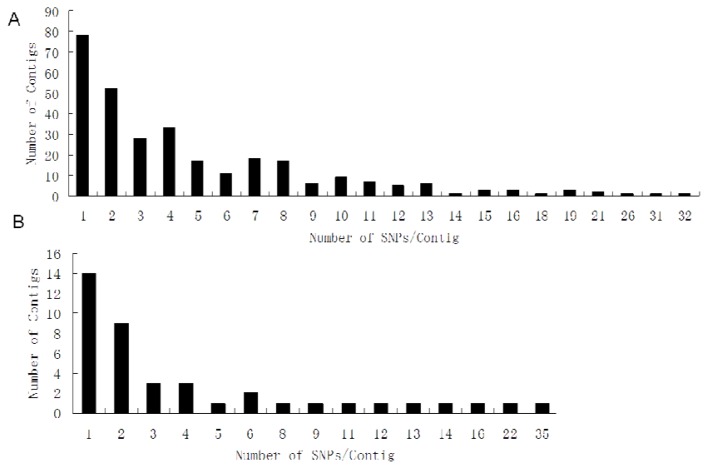
Distribution of putative and actual single nucleotide polymorphisms (SNPs) per contig in common carp. (**A**) Distribution of putative SNPs discovered from online expressed sequence tags (ESTs); (**B**) Distribution of validated and new SNPs detected in the Chinese common carp ESTs.

The alignment of 14,066 online common carp ESTs resulted in the identification of 8862 unigenes (6727 singletons and 2135 contigs). The number of EST sequences per contig ranged from two to 60 (mean 3.4). Based on searches of the 2135 contigs, a total of 49,476 variations were detected, including 40,749 nucleotide substitutions and 8509 indels. After the additional filtering criterion was applied, a total of 1487 putative SNPs were obtained from 303 contigs, with an average frequency of one SNP per 173 bp of contig sequence, which was greater than frequencies reported in Atlantic salmon (one SNP per 614 bp) [10], brown trout (one SNP per 463 bp) [14] and Pacific salmon (one SNP per 239 bp) [15], but close to the frequency reported in chum salmon (one SNP per 175 bp) [15]. The distribution of SNPs in these EST contigs ranged from one to 32 (Figure 1). Of the unigenes described in this study, 4127 (46.7%) were annotated, and the annotations for the contigs containing valid SNPs in the Chinese common carp are listed in Table 1.

**Figure 2:**
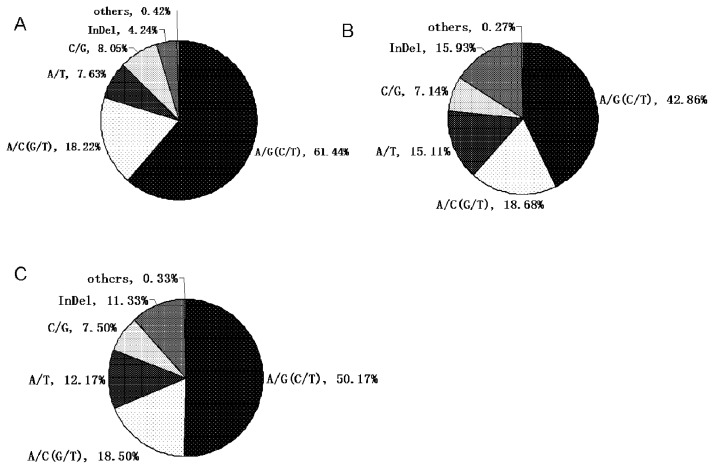
Distribution of different kinds of nucleotide variances in exons (**A**), introns (**B**) and complete sequences (**C**) in successfully amplified sequences.

Of the 81 pairs of primers, 48 produced PCR products. Among these, 30 amplified products were larger than the expected size, indicating the existence of introns (Tables 1,S1). A total length of *ca*. 27,010 bp was amplified and sequenced, of which 13,213 bp were exons and 13,797 bp were introns. Finally, 121 SNPs were identified from 13,213 bp of exons, and 26 (21.5%) of these putative SNPs were verified in a Chinese common carp population. On the other hand, a large number of new SNPs, which were not detected in the SNP discovery by data mining from the online EST sequences, were identified during the validation. These new SNPs included 202 SNPs and 10 indels in exons, and 306 SNPs and 58 indels in introns (Table 1, Figure 2, Supplement Material 1). The distribution of SNPs in exon sequences ranged from one to 34 (Figure 1). In addition, the average rates of occurrence were about one SNP per 58 bp in the exon sequences, one SNP per 45 bp in the intron sequences, and one SNP per 53 bp in the complete genomic sequences. These occurrence rate are much higher than the rates of putative SNPs discovered from the online EST databases in most fish species, but quite close to that of the hybridized catfish (1.32 SNP per 100 bp) [16]. In the present study, although the validation rate of predicted SNPs from online ESTs of European originated common carp was low, many new SNPs were found during the validation by sequencing the PCR products from the Chinese carp. These findings may suggest that different common carp breeds or geographic populations have different levels of genetic divergence and population structures, which are indicated by the gain or loss of SNP loci at a great level. However, these results should be interpreted with caution since the SNP frequency in discovery could be affected by many factors, such as sequencing depth, number of samples and population variation. Another factor that may be involved in this phenomenon is the higher rates of genome duplications in the common carp as indicated in other tetraploid fish such as some salmonids [15,17,18].

Among the 228 exon SNPs, 145 were transitions (81 C/T, 64 A/G) and 82 were transversions (27 A/C, 19 C/G, 18 A/T, 18 G/T), while in intron SNPs there were 154 transitions and 149 transversions (Table 2). Two SNPs had both transition and transversion (C/G/T and A/G/T), this type of SNP has also been detected in Atlantic cod [11]. The ratio of transitions to transversions (t_s_/t_v_) was 1.77 for exon SNPs, 1.05 for intron SNPs and 1.31 for SNPs in complete genomic sequences. These ratios are similar to those reported in turbot (1.885) [12], chinook salmon (1.49) [15], gilthead seabream (1.375) [9] and zebrafish (1.20) [19]. However, in other fishes, such as chum salmon (t_s_/t_v_ = 0.95) and sockeye salmon (t_s_/t_v_ = 0.98) [15], the t_s_/t_v_ ratios were significantly lower, and only close to the ratio estimated from the intron regions of common carp ESTs (1.05) in this study. This discrepancy in t_s_/t_v_ ratios may suggest biased codon usage or substitution rate because fishes from different phylogenetic units may be subject to different selection pressures. Comparison of exon and intron SNPs revealed extreme differences in almost all aspects, including SNP frequency, t_s_/t_v_ value, and commonest and rarest SNP types (Figure 2, Table 2). This may be explained by dissimilar natural selection pressures on exons and introns. In exons, most destructive mutations, which cause loss of protein function, vanish with less fit individuals, while beneficial mutations are retained and accumulate during evolution. Conversely, evolutionary constraints on introns are relatively weak, and more variations could be retained [20,21].

Twenty-three SNPs were chosen to test the polymorphisms in 38 unrelated individuals from a Yangtze River common carp population. All these loci were found to be polymorphic, with frequencies of minor allele ranging from 0.053 to 0.368 (mean 0.19; Table 3), observed heterozygosity from 0.053 to 0.526 (mean 0.262; Table 3), and polymorphism information content (PIC) from 0.095 to 0.357 (mean 0.246; Table 3). In the exact test for HWE, 21 SNPs were in HWE (*p* > 0.05), and 2 SNPs deviated significantly from HWE after Bonferroni corrections (*p* < 0.00217). Linkage disequilibrium (LD) was detected between 19 pairs of the SNP loci after Bonferroni corrections (*p* < 0.00020).

## 3. Experimental Section

### 3.1. Detection and Annotation of Putative SNPs

A total of 14,192 EST sequences of common carp (mainly European breeds) were downloaded from GenBank, DNA Databank of Japan (DDBJ) and European Molecular Biology Laboratory (EMBL) databases. Sequences of length less than 100 bases were removed, and the remaining 14,066 EST sequences were used for further processing. Overlapping sequences were identified by cluster analysis using the UIcluster v. 2.02 software [22]. After this, the ESTs were divided into many clusters, and then alignment was performed using the Phrap program [23] for every cluster containing more than four sequences. SNPs were detected by the autoSNP program [24], with an additional criterion that the appearance of the minor allele of a given locus in the assembly of overlapping sequences must occur at least two times. Complete contigs containing SNPs were compared (BLASTX) to the UniProt_SWISSProt database and annotated with the top BLASTX hit if the database match had an *e*-value of ≤10^−5^. Matches to hypothetical gene and protein sequences were filtered out.

### 3.2. Validation and Characterization of SNPs

One hundred and ninety-seven SNPs (Supplement material 2) from 73 contigs were randomly selected for validation, and 81 pairs of primers were designed using the Primer 5 software to amplify the genomic fragments containing these SNPs via polymerase chain reaction (PCR). The validation panel contained eight individuals from three different common carp populations (breeds) (Yangtze River common carp *n* = 4, Wuyuan (purse) red carp *n* = 2, and Xingguo red carp *n* = 2). A population of common carp with 38 unrelated individuals from the Zhangdu Lake of the Yangtze River, Wuhan, China, was used for polymorphism analysis of selected valid SNPs. Genome DNA was extracted from fin clips following the standard phenol–chloroform protocol [25].

PCR amplifications were carried out in a thermal cycler (MyCycler, BIO-RAD) in 60 μL reaction volumes containing 6 μL of 10× reaction buffer, 2 μL of dNTP (10 mmol/l), 2 U of *Taq* polymerase (TIANGEN, China), 3.5 μL of forward and reverse primer mixture (2.5 mmol/L), 3.5 μL of template DNA and 44 μL of sterile water, using the following program: 94 °C for 5 min, followed by 35 cycles of 94 °C for 40 s, optimum annealing temperature for 40 s, and 72 °C for 60 s, and a final extension of 72 °C for 7 min. PCR amplicons were purified using the Gel Extraction System B (BioDev-tech, China), according to the manufacturer’s instructions. Purified PCR products were cloned into the PMD18-T vector and sequenced on an ABI 3730XL machine (Majorbio, China). For SNP genotyping, PCR-restricted fragment length polymorphism [26], direct sequencing and fragment length discrepant allele specific-PCR [27] were carried out for a given SNP.

### 3.3. Data Analysis

Alignment of the sequenced fragments was performed using ClustalX v. 1.81 [28], and putative SNP and small indels were detected using autoSNP program [24] and checked manually. Polymorphic indices were calculated using the Popgene v. 1.31 [29] and Excel Microsatellite Toolkit [30] software. The fitness to the Hardy-Weinberg equilibrium (HWE) at each locus, and pairwise linkage disequilibrium (LD) were tested for all validated SNPs using the Arlequin v. 3.1 software [31].

## 4. Conclusions

A total 1487 putative SNPs were identified by mining from online common carp EST sequences. Approximately 320 of the putative SNPs are expected to be true in the Chinese common carp as estimated based on the validation rate of 21.5% in this study. In spite of the low validation rate, large numbers of new SNPs were identified in the Chinese common carp. Of the 23 SNPs tested, all loci were polymorphic in a Yangtze River population with moderate diversity. These results indicate that the occurrence of SNPs varies significantly between European and Chinese populations, and these characterized SNPs are valuable resources for population genetics, high**-**resolution genetic maps, QTL (quantitative trait locus) identification, and maker assisted breeding in the common carp.

## Supplementary Materials

Supplementary Material 1

## Figures and Tables

**Table 1:** Validation of putative SNPs in the Chinese common carp and annotation for the SNP-containing EST sequences.

Cluster ID	Primers designed	Length (bp, anticipative/actual)	Number of SNPs (putative/validated)	New SNPs in exon/intron	Annotation
Cyprinus_Cluster7873.seq.Contig1	7873-1	180/388	1/0	14/32(5) a	extracellular space
	7873-2	196/313	1/0	4/0	
Cyprinus_Cluster7877.seq.Contig1	7877	163/250	6/1	0/0	malate dehydrogenase (oxaloacetate-decarboxylating) (NADP+) activity
Cyprinus_Cluster7885.seq.Contig1	7885	215/215	1/0	1(2)/−	actin binding
Cyprinus_Cluster7889.seq.Contig1	7889	319/822	1/0	0/0	cornified envelope
Cyprinus_Cluster7892.seq.Contig1	7892	253/377	4/1	0/0	structural constituent of ribosome
Cyprinus_Cluster7895.seq.Contig1	7895-2	157/954	1/0	2/11(5)	cerebroside-sulfatase activity
Cyprinus_Cluster7896.seq.Contig1	7896	448/450	1/0	1/−	nucleus
Cyprinus_Cluster7898.seq.Contig1	7898	238/238	0/0	13/−	creatine kinase activity
Cyprinus_Cluster7909.seq.Contig1	7909	533/533	2/0	13/−	N.A. b
Cyprinus_Cluster7918.seq.Contig1	7918	278/550	1/1	1/0	cathepsin H activity
Cyprinus_Cluster7921.seq.Contig1	7921	164/319	1/0	1/2	N.A.
Cyprinus_Cluster7927.seq.Contig1	7927	322/322	1/1	1/−	N.A.
Cyprinus_Cluster7929.seq.Contig1	7929	222/1433	1/0	1/13(3)	3-hydroxyanthranilate 3,4-dioxygenase activity
Cyprinus_Cluster7933.seq.Contig1	7933	429/1016	1/1	34(1)/55(18)	glutathione transferase activity
Cyprinus_Cluster7943.seq.Contig1	7943	402/405	4/0	1(2)/−	N.A.
Cyprinus_Cluster7944.seq.Contig1	7944	298/298	4/0	5/−	GTPase activity
Cyprinus_Cluster7947.seq.Contig1	7947	236/419	2/1	2/3	lysozyme activity
Cyprinus_Cluster7953.seq.Contig1	7953	347/347	1/1	7/−	serine-type endopeptidase inhibitor activity
Cyprinus_Cluster7955.seq.Contig1	7955	240/357	3/0	0/0	actin binding
Cyprinus_Cluster7957.seq.Contig1	7957	354/1428	5/0	2/17(2)	fructose-bisphosphate ldolase activity
Cyprinus_Cluster7961.seq.Contig1	7961	222/418	2/1	21/26(5)	external side of plasma membrane
Cyprinus_Cluster7969.seq.Contig1	7969	217/216	4/4	7/−	antigen binding
Cyprinus_Cluster7974.seq.Contig1	7974	215/443	6/0	1/5	N.A.
Cyprinus_Cluster7980.seq.Contig1	7980-2	211/529	7/0	1/13(1)	l-lactate dehydrogenase activity
Cyprinus_Cluster7986.seq.Contig1	7986-2	191/404	1/0	4/17	binding
Cyprinus_Cluster7988.seq.Contig1	7988	184/324	1/0	16/30(2)	receptor activity
Cyprinus_Cluster7990.seq.Contig1	7990	273/1301	2/1	(2)/3	ubiquitin-protein ligase activity
Cyprinus_Cluster7992.seq.Contig1	7992	139/139	1/0	1/−	adenyl-nucleotide exchange factor activity
Cyprinus_Cluster8000.seq.Contig1	8000	419/709	2/0	2/3	glucose-6-phosphatase activity
Cyprinus_Cluster8001.seq.Contig1	8001	376/1412	2/1	2/4(2)	N.A.
Cyprinus_Cluster8008.seq.Contig1	8008	233/1715	2/1	0/0	integral to membrane
Cyprinus_Cluster8009.seq.Contig1	8009-2	213/412	3/1	3/3	calcium ion binding
Cyprinus_Cluster8011.seq.Contig1	8011	303/302	2/0	2/−	thyroxine 5′-deiodinase activity
Cyprinus_Cluster8012.seq.Contig1	8012	203/203	2/0	1(1)/0	N.A.
Cyprinus_Cluster8013.seq.Contig1	8013	260/257	5/1	1/0	N.A.
Cyprinus_Cluster8017.seq.Contig1	8017	313/1384	3/0	(1)/6	regulation of progression through cell cycle
Cyprinus_Cluster8021.seq.Contig1	8021	447/656	8/0	6/1	N.A.
Cyprinus_Cluster8025.seq.Contig1	8025	318/875	2/2	0/7	steroid binding
Cyprinus_Cluster8034.seq.Contig1	8034	327/327	2/0	2/−	bisphosphoglycerate mutase activity
Cyprinus_Cluster8041.seq.Contig1	8041	173/329	1/0	0/2	cytoplasm
Cyprinus_Cluster8048.seq.Contig1	8048	256/989	2/0	1/0	dolichyl-diphosphooligosaccharide-protein glycotransferase activity
Cyprinus_Cluster8050.seq.Contig1	8050-1	179/179	1/0	6(1)/−	signal transducer activity
Cyprinus_Cluster8052.seq.Contig2	8052cg2	286/792	1/1	13/53(15)	l-lactate dehydrogenase activity
Cyprinus_Cluster8123.seq.Contig1	8123	428/428	7/3	6/0	mitochondrion
Cyprinus_Cluster8142.seq.Contig1	8142	420/420	7/3	1/0	N.A.
Cyprinus_Cluster8184.seq.Contig1	8184	413/413	3/0	12/0	N.A.
Total	47	13,213/27,010	121/26	202(10)/306(58)	-

a Values in parenthesis stand for the numbers of indels;

b N.A.: not annotated.

**Table 2:** Numbers of transitions and transversions in different genomic regions of common carp.

	Transitions		Transversions				Multiple
	
	A/G	C/T	A/C	A/T	G/C	GT	
In exons	64	81	27	18	19	18	1C/G/T
In introns	88	68	29	55	26	39	1A/G/T
In complete sequences	152	149	56	73	45	55	2

**Table 3:** Characterization of 23 polymorphic SNPs in a test population of the Chinese common carp.

Loci	Allele Frequencies	*H*_e_	*H*_o_	PIC	*p*-value
CC a 7892G > A	0.355(A)/0.645(G)	0.464	0.342	0.403	0.15446
CC7953C > G	0.263(G)/0.737(C)	0.393	0.526	0.313	0.04006
CC7943G > A	0.316(A)/0.684(G)	0.438	0.474	0.339	0.71678
CC7909-1G > A	0.355(A)/0.645(G)	0.464	0.500	0.353	0.73065
CC7909-2T > C	0.145(C)/0.855(T)	0.251	0.237	0.217	0.57020
CC7909-3A > G	0.25(G)/0.75(A)	0.380	0.395	0.305	1.00000
CC7909-4A > T	0.145(T)/0.855(A)	0.251	0.237	0.217	0.56984
CC7909-5T > C	0.145(C)/0.856(T)	0.251	0.237	0.217	0.56926
CC7909-6G > C	0.145(C)/0.857(G)	0.251	0.237	0.217	0.56908
CC7909-7T > A	0.092(A)/0.908(T)	0.169	0.079	0.153	0.01852
CC7909-8A > G	0.079(G)/0.921(A)	0.147	0.105	0.135	0.19207
CC7909-9T > A	0.066(A)/0.934(T)	0.125	0.079	0.115	0.13050
CC7909-10T > C	0.224(C)/0.776(T)	0.352	0.184	0.287	0.00768
CC7909-11T > C	0.092(C)/0.908(T)	0.169	0.079	0.153	0.01862
CC7909-12T > A	0.092(A)/0.909(T)	0.169	0.079	0.153	0.01853
CC7909-13T > C	0.368(C)/0.632(T)	0.472	0.158	0.357	0.00003
CC7969-1G > T	0.053(T)/0.947(G)	0.101	0.053	0.095	0.07912
CC7969-2C > A	0.290(A)/0.710(C)	0.417	0.158	0.327	0.00030
CC7969-3C > G	0.111(G)/0.889(C)	0.200	0.111	0.178	0.04048
CC7969-4A > G	0.167(G)/0.833(A)	0.282	0.333	0.239	0.55952
CC7969-5A > C	0.236(C)/0.764(A)	0.366	0.472	0.296	0.15560
CC7969-6A > G	0.236(G)/0.764(A)	0.366	0.472	0.296	0.15479
CC7969-7T > G	0.236(G)/0.764(T)	0.366	0.472	0.296	0.15639

a CC is the abbreviation of the cluster ID prefix “Cyprinus_Cluster”.
