# Supplementary material for: Development and Characterization of New Single Nucleotide Polymorphism Markers from Expressed Sequence Tags in Common Carp (Cyprinus carpio)
Source: Int J Mol Sci. 2012 Jun 14;13(6):7343–53. doi: 10.3390/ijms13067343 (PMC3397529; doi:10.3390/ijms13067343)
Supplement: Supplementary Material 1 [file ijms-13-07343-s001.pdf]

Development and Characterization of New Single Nucleotide Polymorphism Markers from Expressed Sequence Tags in Common Carp (*Cyprinus carpio*)

Supplementary Material 1

Location of new-found SNPs (regions with red characters are introns).

1.

```
                   1 1111111112 2222222223 3333333334 4444444445 5555555556 6666666667 7777777778
          1234567890 1234567890 1234567890 1234567890 1234567890 1234567890 1234567890 1234567890
7873-1-3 gtggcaacag tgctaaagtg atgcttaaag actgtattgc tgactgtcca agtggatcca tcaacctcgg cattgcaaaa
7873-1-5 gtggcaacag tgctaaagtg atgcttaaag actgtattgc tgactgtcca agtggatcca tcaacctcgg cattgcaaaa
7873-1-4 gtggcaacag tgctaaagtg atgcttaaag actgtattgc tgactgtcca agtggatcca tgaacctcgg cattgcaaaa
7873-1-1 gtggcaacag tgctaaagtg aagcttaaag actgtgctgc tgactgtgca agtggatcca tgaacttcgg ctatgcaaag
7873-1-2 gtggcaacag tgctaaagtg aagcttaaag actgtgctgc tgactgtgca agtggatcca tgaacttcgg ctatgcaaag

                              1 1111111111 1111111111 1111111111 1111111111 1111111111 1111111111
          8888888889 9999999990 0000000001 1111111112 2222222223 3333333334 4444444445 5555555556
          1234567890 1234567890 1234567890 1234567890 1234567890 1234567890 1234567890 1234567890
7873-1-3 acatcttttg cgtgctgtaa cacagaccag tgtaacgtcc aagacgctcc aggtatcata ctctactgac ca-aatgcaa
7873-1-5 acatcttttg cgtgctgtaa cacagaccag tgtaacgtcc aagacgctcc aggtatcata ctctactgac ca-aatgcaa
7873-1-4 acatcttttg cgtgctgtaa cacagaccag tgtaacgtcc aagacgctcc aggtatcata ctctactgac ca-aatgcaa
7873-1-1 acatctttaa cgtgctgtat cacagaccgg tgtaacgtcc aagacactcc aggtatcagg ccttattgac cataaaataa
7873-1-2 acatctttaa cgtgctgtaa cacagaccgg tgtaacgtcc aagacactcc aggtatcagg ctttattgac cataaaataa

          1111111111 1111111111 1111111111 1111111112 2222222222 2222222222 2222222222 2222222222
          6666666667 7777777778 8888888889 9999999990 0000000001 1111111112 2222222223 3333333334
          1234567890 1234567890 1234567890 1234567890 1234567890 1234567890 1234567890 1234567890
7873-1-3 ataactatct ttaaggtcaa ctcagatttt tcttccaaag gacattc--- -attcaggaa atacaataca gggtgcggct
7873-1-5 ataactatct ttaaggtcaa ctcagatttt tcttccaaag gacattc--- -attcaggaa atacaataca gggtgcggct
7873-1-4 ataactatct ttaaggtcaa ctcagatttt tcttccaaag gacattc--- -attcaggaa atacaataca gggtgcggct
```

```
7873-1-1 ataa--atct ttaaggtcaa tactcatttt acttccaaag gagatcagga aatacaaatt atacaatacа gagtgcagct
7873-1-2 ataa--atct ttaaggtcaa tactcatttt acttccaaag gagatcagga aatacaaatt atacaatacа gagtgcagct

         2222222222 2222222222 2222222222 2222222222 2222222222 2222222223 3333333333 3333333333
         4444444445 5555555556 6666666667 7777777778 8888888889 9999999990 0000000001 1111111112
         1234567890 1234567890 1234567890 1234567890 1234567890 1234567890 1234567890 1234567890
7873-1-3 tgtcagttgt gaactgatta agtggtgcag ctaacagagt gttatttcat tagat----- ---------- --aaataaaa
7873-1-5 tgtcagttgt gaactgatta agtggtgcag ctaacagagt gttatttcat tagat----- ---------- --aaataaaa
7873-1-4 tgtcagttgt gaactgatta agtggtgcag ctaacagagt gttatttcat tagat----- ---------- --aaataaaa
7873-1-1 tgtcagttgt gaatttattg attggaacag ctaacagagt gttatttcat tacatgttat attttgcaca gaagataaaa
7873-1-2 tgtcagttgt gaatttattg attggaacag ctaacagagt gttatttcat tacatgttat attttgcaca gaagataaaa

         3333333333 3333333333 3333333333 3333333333 3333333333 3333333333 3333333333 3
         2222222223 3333333334 4444444445 5555555556 6666666667 7777777778 8888888889 9
         1234567890 1234567890 1234567890 1234567890 1234567890 1234567890 1234567890 1
7873-1-3 taatacattt gttttatcct gtagatcctt ctaatgtccc caacggaaag acgtgttact cttgtgatgg a
7873-1-5 taatacattt gttttatcct gtagatcctt ctaatgtccc caacggaaag acgtgttact cttgtgatgg a
7873-1-4 taatacattt gttttatcct gtagatcctt ctaatgtccc caacggaaag acgtgttact cttgtgatgg a
7873-1-1 caatgc-ttg tttttgtcct gtagatcctt ctattgtccc caatggaaag acgtgttact cttgtgatgg a
7873-1-2 caatgc-ttg tttttgtcct gtagatcctt ctaatgttcc caatggaaag acgtgttact cttgtgatgg a
```

SNP LOCATIONS: 22A/T, 36A/G, 37T/C, 48C/G, 62C/G, 66C/T, 72A/T, 73A/T, 80A/G, 89A/T, 90A/G, 109A/G, 126A/G, 139A/G, 140T/G, 143C/T, 146C/T, 156A/T, 157A/G, 158C/T, 181C/T, 182A/T, 184A/T, 185C/G, 191A/T, 203C/G, 220A/T, 232A/G, 237A/G, 254C/T, 256G/T, 260A/G, 262G/T, 266A/T, 267A/G, 293C/G, 314A/G, 321C/T, 325A/G, 330T/G, 331T/G, 336A/G, 364C/T

2.

```
                   1 1111111112 2222222223 3333333334 4444444445 5555555556 6666666667 7777777778 8888888889 9999999990
          1234567890 1234567890 1234567890 1234567890 1234567890 1234567890 1234567890 1234567890 1234567890 1234567890
7873-2-2 tccttctaat gtccccaacg g-aaagacgt gttactcttg tgatggacag agctgctcaa acatattgag ctgtacaggg aatgaagacc gctgcttgaa
7873-2-3 tccttctaat gtccccaacg g-aaagacgt gttactcttg tgatggacag agctgctcaa acatattgag ctgtacaggg aatgaagacc gctgcttgaa
7873-2-1 tccttctaat gtccccaacg g-aaagacgt gttactcttg tgatggacag agctgctcaa acatattgag ctgtacaggg aatgaagacc gctgcttgaa
7873-2-4 tccttctaat gtccccaacg g-aaagacgt gttactcttg tgatggacag agctgctcaa acatattgag ctgtacaggg aatgaagacc gctgcttgaa
7873-2-0 tccttctaat gtccccaacg ggaaagacgt gttactcttg tgatggacag agctgctcaa acatattgag ctgtacaggg aatgaagacc gctgcttgaa

          1111111111 1111111111 1111111111 1111111111 1111111111 1111111111 1111111111 1111111111 1111111111 1111111112
          0000000001 1111111112 2222222223 3333333334 4444444445 5555555556 6666666667 7777777778 8888888889 9999999990
          1234567890 1234567890 1234567890 1234567890 1234567890 1234567890 1234567890 1234567890 1234567890 1234567890
7873-2-2 agcaacaggc agggactttc ggtggccagt caatggttgt aaaaggctgt gtctctaaat ctatctgtga tgctgaaaca tcggttcagg atgttcaggg
7873-2-3 agcaacaggc agggactttc ggtggccagt caatggttgt aaaaggctgt gtctctaaat ctatctgtga tgctgaaaca tcggttcagg atgttcaggg
7873-2-1 agcaacaggc agggactttc ggtggccagt caatggttgt aaaaggctgt gtctctaaat ctatctgtga tgccggatca tcggttcagg atgttcaggg
7873-2-4 agcaacagg- -gggactttc ggtggccagt cattagttgt aaaaggctgt atctctaaat ctttctgtga tgctggatca tcggttcagg atgttcaggg
7873-2-0 agcaacagg- ---gcgtttc ggtggccagt caatggttat aaaaggctgt atctctaaat ctttctgtga tgctggatca tcggttcagg atgttcaggg
```

SNP LOCATIONS: 151G/A, 163A/T, 176G/A, 178T/A

3.

```
                 1 1111111112 2222222223 3333333334 4444444445 5555555556 6666666667 7777777778 8888888889 9999999990
        1234567890 1234567890 1234567890 1234567890 1234567890 1234567890 1234567890 1234567890 1234567890 1234567890
7877-1  taaccccacc agcaaggcag agtgcactgc tgagcagtgc tacacactca ctgaggtgca tttg--agga gtcacatgtg tacatttgtg tgagaaagtg
7877-3  taaccccacc agcaaggcag agtgcactgc tgagcagtgc tacacactca ctgaggtgca tttg--agga gtcacatgtg tacatttgtg tgagagagtg
7877-2  taaccccacc agcaaggcag agtgcactgc tgagcagtgc tacacactca ctgaggtgca tttg--agga gtcacatgtg tacatttgtg tgagagagtg
7877-5  taaccccacc agcaaggcag agtgcactgc tgagcagtgc tacacactca ctgaggtgca tttg--agga gtcacatgtg tacatttgtg tgagagagtg
7877-4  taaccccacc agcaaggcag agtgtaccgc tgagcagtgc tacacactca ctgaggtgct tttggaagga gtagcatgtg tacattcatg tgagagagtg
7877-0  taaccccacc agcaaggcag agtgcactgc tgagcagtgc tacacactca ctgag----- ---------- ---------- ---------- ----------

        1111111111 1111111111 1111111111 1111111111 1111111111 1111111111 1111111111 1111111111 1111111111 1111111112
        0000000001 1111111112 2222222223 3333333334 4444444445 5555555556 6666666667 7777777778 8888888889 9999999990
        1234567890 1234567890 1234567890 1234567890 1234567890 1234567890 1234567890 1234567890 1234567890 1234567890
7877-1  aaacatttgg tttcatttat gactc-tttg t----gtttt atattacagg gacgggggat atttgcaagt ggaagtccct ttgatgtgat aacccttccc
7877-3  aaacatttgg tttcatttat gactc-tttg t----gtttt atattacagg gacgggggat atttgcaagt ggaagtccct ttgatgtgat aacccttccc
7877-2  aaacatttgg tatcatttat gactc-tttg t----gtttt atattacagg gacgggggat atttgcaagt ggaagtccct ttgatgtgat aacccttccc
7877-5  aagcatttgg tttcatttat atttcattta tattggtttt atattacagg gacgggggat atttgcaagt ggaagtccct ttgatgtgat aacccttccc
7877-4  aatcattt-- ---cattttt gc----tttg t----gtttt atattgcagg gacgggggat atttgcaagc ggcagtccct ttgatccagt gacccttccc
7877-0  ---------- ---------- ---------- ---------- ---------g gacgggggat atttgcaagt ggaagtccct ttgatgtgat aacccttccc

        2222222222 2222222222 2222222222 2222222222 2222222222 2222222
        0000000001 1111111112 2222222223 3333333334 4444444445 5555555
        1234567890 1234567890 1234567890 1234567890 1234567890 1234567
7877-1  gatgggcgga agttctaccc tggtcagggc aacaacgctt atgtgttccc tggagtg
7877-3  gatgggcgga agttctaccc tggtcagggc aacaacgctt atgtgttccc tggagtg
7877-2  gatgggcgga agttctaccc tggtcagggc aacaacgctt atgtgttccc tggagtg
7877-5  gatgggcgga agttctaccc tggtcagggc aacaacgctt atgtgttccc tggagtg
7877-4  gatgggcgaa agttctaccc tggtcagggc aacaacgctt atgtgttccc tggagtg
7877-0  gatgggcgaa agttctaccc tggtcagggc aacaacgctt atgtgttccc tggagtg
```

```
SNP LOCATIONS: 209G/A

4.
                 1 1111111112 2222222223 3333333334 4444444445 5555555556 6666666667 7777777778 8888888889 9999999990
        1234567890 1234567890 1234567890 1234567890 1234567890 1234567890 1234567890 1234567890 1234567890 1234567890
7885-1  gtatgctgcc cgccaactat gtggaagcca tctgaataca cctactgtcc tctcaaacac cctttggatg agggagtttg agatccactc aaatggaaaa
7885-5  gtatgctgcc cgccaactat gtggaagcca tctgaataca cctactgtcc tctcaagcac cctttggatg agggagttcg agatccactc aaatggataa
7885-2  gtatgctgcc cgccaactat gtggaagcca tctgaataca cctactgtcc tctcaaacac cctttggatg agggagtttg agatccactc aaatggaaaa
7885-3  gtatgctgcc cgccaactat gtggaagcca tctgaataca cctactgtcc tctcaaacac cctt-ggatg agggagtttg agatccactc aaatggaaaa
7885-0  gtatgctgcc cgccaactat gtggaagcca tctgaataca cctagtgtcc tctcaaacac cctttggatg agggagcttg agatccactc aaatggaaaa
7885-4  gtatgctgcc cgccaactat gtggaagcca tctaaataca actactgcgc tctcaaactc cctgtggatg acggagtttg atatccactc aga-----ac

        1111111111 1111111111 1111111111 1111111111 1111111111 1111111111 1111111111 1111111111 1111111111 1111111112
        0000000001 1111111112 2222222223 3333333334 4444444445 5555555556 6666666667 7777777778 8888888889 9999999990
        1234567890 1234567890 1234567890 1234567890 1234567890 1234567890 1234567890 1234567890 1234567890 1234567890
7885-1  aaacgattta atgtgtact- --gttggtca aagtagttat gcttctgttt tatcctcatt actcttagga aatttccttt gtgcccatgt cacaaagtct
7885-5  aa-ctattta atgtgta--- --gttagtca aagtagttat gcttctgttt tatcctcatt actcttagga aatttccttt gtgcccatgt cacaaagtct
7885-2  aa-cgattta atgtgtact- --gttagtca aagtagttat gcttctgttt tatcctcatt actcttagga aatttccttt gtgcccatgt cacaaagtct
7885-3  aa-cgattta atgtgtact- --gttagtca aagtagttat gcttctgttt tatcctcatt actcttagga aatttccttt gtgcccatgt cacaaagtct
7885-0  aa-cgattta atgtgtacta atgttagtca aagtagttat gcttctgttt tatcctcatt actcttagga aatttccttt gtgcccatgt cacaaagtct
7885-4  aagctatttt atgtgtacta atgttagtca a-gtagttcc gctactgttt -gtcctcatt actgttggga aatttatttt gtgcccatgt cacaaagtct

        2222222222 222222
        0000000001 111111
        1234567890 123456
7885-1  attgtggttg tggcat
7885-5  attgtggttg tggcat
7885-2  attgtggttg tggcat
7885-3  attgtggttg tggcat
7885-0  attgtggttg tggcat
```

```
7885-4 attgtggttg tggcat

SNP LOCATIONS: 105G/T

5.
                1 1111111112 2222222223 3333333334 4444444445 5555555556 6666666667 7777777778 8888888889 9999999990
       1234567890 1234567890 1234567890 1234567890 1234567890 1234567890 1234567890 1234567890 1234567890 1234567890
7889-0 tggcaagcct ctggatgtag cgctgaaagc tgctcttaaa ggtgaactgg aagaagtggt tctgagcttg ttgatgactc cagctcaata tgattctttt
7889-1 tggcaagcct ctggatgtag cgctgaaagc tgctcttaaa ggtgaactgg aagaagtggt tctgagcttg ttgatgactc cagctcaata tgattctttt
7889-5 tggcaagcct ctggatgtag cgctgaaagc tgctcttaaa ggtgaactgg aagaagtggt tctgagcttg ttgatgactc cagctcaata tgattctttt
7889-3 tggcaagcct ctggatgtag cgctgaaagc tgctcttaaa ggtgaactgg aagaagtggt tctgggcttg ttgatgactc cagctcaata tgattctttt
7889-4 tggcaagcct ctggatgtag cgctgaaagc tgctcttaaa ggtgaactgg aagaagtggt tctgagcttg ttgatgactc cagctcaata tgattctttt
7889-2 tggcaagcct ctggatgtag cgctgaaagc tgctcttaaa ggtgaactgg aagaagtggt tctgagcttg ttgatgactc cagctcaata tgattctttt

       1111111111 1111111111 1111111111 1111111111 1111111111 1111111111 1111111111 1111111111 1111111111 1111111112
       0000000001 1111111112 2222222223 3333333334 4444444445 5555555556 6666666667 7777777778 8888888889 9999999990
       1234567890 1234567890 1234567890 1234567890 1234567890 1234567890 1234567890 1234567890 1234567890 1234567890
7889-0 ctgctcaaaa gtgccatgaa gg---gtttg ggcacagatg aagatacgct tgtcgagatt ttggcctcca ggaccaataa agagatccaa gagattaaac
7889-1 ctgctcaaaa gtgccatgaa ggagggtttg ggcacagatg aagatacgct tgtcgagatt ttggcctcca ggaccaataa agagatccaa gagattaaac
7889-5 ctgctcaaaa gtgccatgaa ggagggtttg ggcacagatg aagatacgct tgtcgagatt ttggcctcca ggaccaataa agagatccaa gagattaaac
7889-3 ctgctcaaaa gtgccatgaa ggagggtttg ggcacagatg aagatacgct tgtcgagatt ttggcctcca ggaccaataa agagatccaa gagattaaac
7889-4 ctgctcaaaa gtgccatgaa ggagggtttg ggcacagatg aagatacgct tgtcgagatt ttggcctcca ggaccaataa agagatccaa gaggttaaac
7889-2 ctgctcaaaa gtgccatgaa ggagggtttg ggcacagatg aagatacgct tgtcgagatt ttggcctcca agaccaataa agagatccaa gagattaaac

       2222222222 2222222222 2222222222 2222222222 2222222222 2222222222 2222222222 2222222222 2222222222 2222222223
       0000000001 1111111112 2222222223 3333333334 4444444445 5555555556 6666666667 7777777778 8888888889 9999999990
       1234567890 1234567890 1234567890 1234567890 1234567890 1234567890 1234567890 1234567890 1234567890 1234567890
7889-0 aggttttcaa acaag--aat ataagaagga tctagaggcg gagatcaagt ctgagacaag tggagatttc aggaatgccc tgctttctct ctgcaagg--
7889-1 aggttttcaa acaagggaat ataagaagga tctagaggcg gagatcaagt ctgagacaag tggagatttc aggaatgccc tgctttctct ctgcaaggtt
7889-5 aggttttcaa acaagggaat ataagaagga tctagaggcg gagatcaagt ctgagacaag tggagatttc aggaatgccc tgctttctct ctgcaaggtt
```

```
7889-3 aggttttcaa acaagggaat ataagaagga tctagaggcg gagatcaagt ctgagacaag tggagatttc aggaatgccc tgctttctct ctgcaaggtt
7889-4 aggttttcaa acaagggaat ataagaagga tctagaggcg gagatcaagt ctgagacaag tggagatttc aggaatgccc tgctttctct ctgcaaggtt
7889-2 aggttttcaa acaagggaat ataagaagga tctagaggcg gagatcaagt ctgagacaag tggagatttc aggaatgccc tgctttctct ctgcaaggtt

       3333333333 3333333333 333333333
       0000000001 1111111112 222222222
       1234567890 1234567890 123456789
7889-0 ---ctaccag aagtgaagac agttttgtg
7889-1 aggctaccag aagtgaagac agttttgtg
7889-5 aggctaccag aagtgaagac agttttgtg
7889-3 aggctaccag aagtgaagac agttttgtg
7889-4 aggctaccag aagtgaagac agttttgtg
7889-2 aggctaccag aagtgaagac agttttgtg
SNP LOCATIONS: NULL
```

6.

```
                 1 1111111112 2222222223 3333333334 4444444445 5555555556 6666666667 7777777778 8888888889 9999999990
        1234567890 1234567890 1234567890 1234567890 1234567890 1234567890 1234567890 1234567890 1234567890 1234567890
7892-3  tggaggaaga agcagtcgga cgtgatgagg tttctgctgc gcgtgcggtg ctggcagtac cgtcagctgc cggcccttca ccgggccccg agacccacca
7892-4  tggaggaaga agcagtcgga cgtgatgagg tttctgctgc gcgtgcggtg ctggcagtac cgtcagctgt cggcccttca ccgggccccg agacccacca
7892-5  tggaggaaga agcagtcgga cgtgatgagg tttctgctgc gcgtgcggtg ctggcagtac cgtcagctgt cggcccttca ccgggccccg agacccacca
7892-2  tggaggaaga agcagtcgga cgtgatgagg tttctgctgc gcgtgcggtg ctggcagtac cgtcagctgt cggcccttca ccgggccccg agacccacca
7892-0  tggaggaaga agcagtcgga cgtgatgagg tttctgctgc gtgtgcggtg ctggcagtac cgtcagctgt cggcccttca ccgggccccg agacccacca
7892-1  tggaggaaga agcagtcgga cgtgatgagg tttctgctgc gcgtgcggtg ctggcagtac cgtcagctgt cggcccttca ccgggccccg agacccaccc

        1111111111 1111111111 1111111111 1111111111 1111111111 1111111111 1111111111 1111111111 1111111111 1111111112
        0000000001 1111111112 2222222223 3333333334 4444444445 5555555556 6666666667 7777777778 8888888889 9999999990
        1234567890 1234567890 1234567890 1234567890 1234567890 1234567890 1234567890 1234567890 1234567890 1234567890
7892-3  gacccgataa agcccgcagg ctgggataca aggccaagca gggcaggcta cgtcatctac cgcatacgtg tgcgccgtgg aggccgcaag cgcccccgtgc
7892-4  gacccgataa agcccgcagg ctgggataca aggccaagca gggcaggcta cgtcatctac cgcatacgtg tgcgccgtgg aggccgcaag cgccccatgc
7892-5  gacccgataa agcccgcagg ctgggataca aggccaagca gggcaggcta cgtcatctac cgcatacgtg tgcgccgtgg aggccgcaag cgcccccgtgc
7892-2  gacccgataa agcccgcagg ctgggataca aggccaagca gggcaggcta cgtcatctac cgcatacgtg tgcgccgtgg aggccgcaag cgcccccgtgc
7892-0  gacccgataa agcccgcaga ctgggataca aggccaagca gggc----ta cgtcatctac cgcatacgtg tgcgccgtgg aggccgcaag cgcccccgtgc
7892-1  gacccgataa agcccgcaga ctgggataca aggccaagca gggcaggcta cgtcatctac cgcatacgtg tgcgccgtgg aggccgcaag cgcccccgtgc

        2222222222 2222222222 2222222222 2222222222 2222222222 2222222
        0000000001 1111111112 2222222223 3333333334 4444444445 5555555
        1234567890 1234567890 1234567890 1234567890 1234567890 1234567
7892-3  ctaaaggtgc cacctacggc aaacccgtgc accacggcgt caaccagatc aagtttg
7892-4  ctaaaggtgc cacctacggc aaacccgtgc accacggcgt caaccagatc aagtttg
7892-5  ctaaaggtgc cacctacggc aaacccgtgc accacggcgt caaccagatc aagtttg
7892-2  ctaaaggtgc cacctacggc aaacccgtgc accacggcgt caaccagatc aagtttg
7892-0  ctaaaggtgc cacctacggc aaacccgtgc accacggcgt caaccagatc aagtttg
7892-1  ctaaaggtgc cacctacggc aaacccgtgc accatggcgt caaccagatc aagtttg
```

SNP LOCATIONS: 120G/A

7.

```
                   1 1111111112 2222222223 3333333334 4444444445 5555555556 6666666667 7777777778 8888888889 9999999990
          1234567890 1234567890 1234567890 1234567890 1234567890 1234567890 1234567890 1234567890 1234567890 1234567890
7895-2-3  tccccgttct catggacatc atcgaa-gga gaactggtga gctggacact tccatctctt tccttttagc taattcagta gtataattcc ccttttagtt
7895-2-4  tccccgttct catggacatc atcgaa-gga gaactggtga gctggacact tccatctctt tccttttagc taattcagta gtataattcc ccttttagtt
7895-2-2  tccccgttct catggacatc atcgaa-gga gaactggtga gctggacact tccatctctt tccttttagc taattcagta gtataattcc ccttttagtt
7895-2-1  tccccgttct catggacatc atcgaa-gga gaactggtga gctggacact tccatctctt tccttttagc taattcagta atataattcc ccttttagtt
7895-2-5  tccccgttct catggacatc atcgaa-gga gaactggtga gctggacact tcattctctt tccttttagc taattcagta gtataattcc ccttttagtt
7895-2-6  tccccgttct catggacatc atcgaaagga gaactggtga gctggacact tccatctctt tccttttagc taattcagta gtataattcc ccttttagtt

          1111111111 1111111111 1111111111 1111111111 1111111111 1111111111 1111111111 1111111111 1111111111 1111111112
          0000000001 1111111112 2222222223 3333333334 4444444445 5555555556 6666666667 7777777778 8888888889 9999999990
          1234567890 1234567890 1234567890 1234567890 1234567890 1234567890 1234567890 1234567890 1234567890 1234567890
7895-2-3  ggtggtagtt tgtgatttga aaacggtgtt gaacacccac cacgtaccaa acacaagctc ttttggagca gaaaggcaaa catgctgttt aagacttaat
7895-2-4  ggtggtagtt tgtgatttga aaacggtgtt gaacacccac cacgtaccaa acacaagctc ttttggagca gaaaggcaaa catgctgttt aaaacttaat
7895-2-2  ggtggtagtt tgtgatttga aaacggtgtt gaacacccac cacgtaccaa acacaagctc ttttggagca gaaaggcaaa catgctgttt aaaacttaat
7895-2-1  ggtggtagtt tgtgatttga aaacagtgtt gaacacccac cacgtaccaa acacaagctc ttttggagca gaaaggcaaa catgctgttt aaaacttaat
7895-2-5  ggtggtagtt tgtgatttgt aaacggtgtt gaacacccac cacgtaccaa tcacgagctc ttttggagca gaaaggcaaa catgctgttt aaaacttaat
7895-2-6  ggtggtagtt tgtgatttga aaacggtgtt gaacacccac cacgtaccaa acacaagctc ttttggagca gaaaggcaaa catgctgttt aaaacttaat

          2222222222 2222222222 2222222222 2222222222 2222222222 2222222222 2222222222 2222222222 2222222222 2222222223
          0000000001 1111111112 2222222223 3333333334 4444444445 5555555556 6666666667 7777777778 8888888889 9999999990
          1234567890 1234567890 1234567890 1234567890 1234567890 1234567890 1234567890 1234567890 1234567890 1234567890
7895-2-3  gaacagtctg catggttcca atccaaaact aattaaattg tgtatgtgta ta--tgt--- -gtgtaattt tacggtttaa aaaaaa-ttg tttttaat--
7895-2-4  gaacagtctg catggttcca atccaaaact aattaaattg tgtatgtgta ta--tgt--- -gtgtaattt tacggtttaa aaaaaaattg tttttaat--
7895-2-2  gaacagtctg catggttcca atccaaaacg aattaaattg tgtatgtgta ta--tgt--- -gtgtaattt tactgtttaa aaaaaaattg tttttaat--
7895-2-1  gaacagtctg catggttcca atccaaaact aattaaattg tgtatgtgta ta--tgt--- -gtgtaattt tactgtttaa aaaaaaattg tttttaat--
7895-2-5  gaacagtctg catggttcca atccaaaact tattaaattg tgtatgtata ta--tgtatg tgtgtaattt tactgtttaa aaat---ttt tttttaacaa
```

```
7895-2-6 gaacagtctg catggttcca atccaaaact aattaaattg tatatgtgta tacatgtatg tgtgtaagtt tactgtttaa a------ttt tttttaacaa

         3333333333 3333333333 3333333333 3333333333 3333333333 3333333333 3333333333 3333333333 3333333333 3333333334
         0000000001 1111111112 2222222223 3333333334 4444444445 5555555556 6666666667 7777777778 8888888889 9999999990
         1234567890 1234567890 1234567890 1234567890 1234567890 1234567890 1234567890 1234567890 1234567890 1234567890
7895-2-3 gaatcataaa tacgaatgta cttatgaatg tattt---ga tgctgacaat taaaaaa-tg acaaataaca gtcatggcat gactgttact ctaactatag
7895-2-4 gaatcataaa tacgaatgta cttatgaatg tattt---ga tgctgacaat taaaaaa-tg acaaataaca gtcatggcat gactgttact ctaactatag
7895-2-2 gaatcataaa tacgaatgta cttatgaacg tattt---ga tgctgacaat taaaaaa-tg acaaataaca gtcatggcat gactgttact ctaactatag
7895-2-1 gaatcataaa tacgaatgta cttatgaatg tattt---ga tgctgacaat taaaaaa-tg acaaataaca gtcatggcat gactgttact ctaactatag
7895-2-5 gaatcataaa tacgaatgta cttatgaacg tatttcttga tgctgaaaat taaaaaaatg acaaataa-- gtcatggcat gactgttact ctaactatag
7895-2-6 gaatcataaa tacgaatgta cttatgaatg tatttcttga tgctgaaaat taaaaaaatg acaaataaca gtcatggca- ------tact ctaactatag

         4444444444 4444444444 4444444444 4444444444 4444444444 4444444444 4444444444 4444444444 4444444444 4444444445
         0000000001 1111111112 2222222223 3333333334 4444444445 5555555556 6666666667 7777777778 8888888889 9999999990
         1234567890 1234567890 1234567890 1234567890 1234567890 1234567890 1234567890 1234567890 1234567890 1234567890
7895-2-3 agtgatacag tagagaatat tcattgcttc ccaccaacta gtggattttt agcaatttgt gcttttctaa aaagccacaa atcagtgaca ttcaaacatg
7895-2-4 agtgatacag tagagaatat tcattgcttc ccaccaacta gtggattttt ggcaatttgt gcttttctaa aaagccacaa atcagtgaca ttcaaacatg
7895-2-2 agtgatacag tagagaatat tcattgcttc ccaccaacta gtggattttt agcaatttgt gcttttctaa aaagccacaa atcagtgaca ttcaaacatg
7895-2-1 agtgatacag tagagaatat tcattgcttc ccaccaacta gtggattttt agcaatttgt gcttttctaa aaagccacaa atcagtgaca ttcaaacatg
7895-2-5 agtgatatag tagagaatat tcattgcttc ccaccaacta gtggattttt agcaatttgt gcttttctaa aaagccacaa atcactgaca ttcaaacatg
7895-2-6 agtgatacag tagagaatat tcattgcttc ccaccaacta gtggattttt agcaatttgt gctttcctaa aaagccacaa atcagtgaca ttcaaacatg

         5555555555 5555555555 5555555555 5555555555 5555555555 5555555555 5555555555 5555555555 5555555555 5555555556
         0000000001 1111111112 2222222223 3333333334 4444444445 5555555556 6666666667 7777777778 8888888889 9999999990
         1234567890 1234567890 1234567890 1234567890 1234567890 1234567890 1234567890 1234567890 1234567890 1234567890
7895-2-3 ctggcggaaa aatttatcaa accacaattt agagaagtta ctcttaccat caaagtcatg aaattatctt agtgggtaaa atctgaaacc agggtcttta
7895-2-4 ctggcggaaa agtttatcaa accacaattt agagaagtta ctcttaccat caaagtcatg aaattatctt agtgggtaaa atctgaaacc agggtcttta
7895-2-2 ctggcggaaa aatttatcaa accacaattt agagaagata ctcttaccat caaagtcatg aaattatttt agtgggtaaa atctgaaacc agggtcttta
7895-2-1 ctggcggaaa aatttatcaa accacaattt agagaagtta ctcttaccat caaagtcatg aaattatctt agtgggtaaa atctgaaacc agggtcttta
7895-2-5 ctgttggaaa aattgatcaa accacaattt agagaagtta ctcttaccat caaagtcatg aaatgatctt agtgggtaaa atctgaaacc agggtcttta
```

```
7895-2-6 ctggtggaaa atttgatcaa accacaa--- agagaagtta ctcttaccat caaagtcatg aagtgatctt agtgggtaaa agctgaaacc aggctcttta

         6666666666 6666666666 6666666666 6666666666 6666666666 6666666666 6666666666 6666666666 6666666666 6666666667
         0000000001 1111111112 2222222223 3333333334 4444444445 5555555556 6666666667 7777777778 8888888889 9999999990
         1234567890 1234567890 1234567890 1234567890 1234567890 1234567890 1234567890 1234567890 1234567890 1234567890
7895-2-3 atttttatta ctccaactaa atgctttggt ctgactagac tggtctagta ccgctaagat ggttagctat ccacaaagca gcctttgtgt aataggctct
7895-2-4 atttttatta ctccaactaa atgctttggt ctgactagac tggtctagta ccgctaagat ggttagctat ccacaaagca gcctttgtgt aataggctct
7895-2-2 atttttatta ctccaactaa atgctttggt ctgactagac tggtctagta ccgctaagat ggttagctat ccacaaagca gcctttgtgt aataggctct
7895-2-1 atttttatta ctccaactaa atgctttggt ctgactagac tggtctagta ccgctaagat ggttagctat ccacaaagca gcctttgtgt aataggctct
7895-2-5 atttttatta ctccaacgaa atgctttggt ctgactagac tggtctagtg ccgctgagat ggttagccat ccacaaagca gcctttgtgt aataggctct
7895-2-6 atttttatta ctccaactaa atgctttggt ttgactagac tggtctagtg ctgataagat ggttagctat ccacaaagca gcctttgtgt aataggccct

         7777777777 7777777777 7777777777 7777777777 7777777777 7777777777 7777777777 7777777777 7777777777 7777777778
         0000000001 1111111112 2222222223 3333333334 4444444445 5555555556 6666666667 7777777778 8888888889 9999999990
         1234567890 1234567890 1234567890 1234567890 1234567890 1234567890 1234567890 1234567890 1234567890 1234567890
7895-2-3 tcctgcccag agaccctcat ctgtcattca cagcatcagc cagtcgtctt ttaaagaact gaagcaaggg gaaatgtatt cagctgctga tcctattggt
7895-2-4 tcctgcccag agaccctcat ctgtcattca cagcatcagc cagtcgtctt ttaaagaact gaagcaaggg gaaatgtatt cagctgctga tcctattggt
7895-2-2 tcctgcccag agaccctcat ctgtcattca cagcatcagc cagtcgtctt ttaaagaact gaagcaaggg gaaatgtatt cagctgctga tcctattggt
7895-2-1 tcctgcccag agaccctcat ctgtcattca cagcatcagc cagtcgtctt ttaaagaact gaagcaaggg gaaatgtatt cagctgctga tcctattggt
7895-2-5 tccttcccag agagcctcat ctgtcattca cagcatcagc cagtcgtctt t-aaagaact gaagcaaggg gaaatgtatt cagctgctga tcctattggt
7895-2-6 tccttcccag agaacctcat ctgtcattca cagcatcagc cagtcgtctt tgaatacatt tccccttgct tcagt-tctt tagctgctga tcctattggt

         8888888888 8888888888 8888888888 8888888888 8888888888 8888888888 8888888888 8888888888 8888888888 8888888889
         0000000001 1111111112 2222222223 3333333334 4444444445 5555555556 6666666667 7777777778 8888888889 9999999990
         1234567890 1234567890 1234567890 1234567890 1234567890 1234567890 1234567890 1234567890 1234567890 1234567890
7895-2-3 ttatataatg ctttacaatg cttctgtgtt cccatcatcc cgcaggatga ccccggtgtg gtgtgcagtg ctatgggtct gtgtgtgtcc cagcaggcag
7895-2-4 ttatataatg ctttacaatg cttctgtgtt cccatcatcc cgcacgatga ccccggtgtg gtgtgcagtg ctatgggtct gtgtgtgtcc cagcaggcag
7895-2-2 ttatataatg ctttacaatg cttctgtgtt cccatcatcc cgcaggatga ccccggtgtg gtgtgcagtg ctatgggtct gtgtgtgtcc cagcaggcag
7895-2-1 ttatataatg ctttacaatg cttctgtgtt cccatcatcc cacaggatga ccccggtgtg gcgtgcagtg ctatgggcct gtgtgtgtcc cagcaggcag
7895-2-5 ttatataatg ctttacaatg cttctgtgtt cccatcatcc cgca-gatga ccccggtgtg gtgtgcagtg ctatgggcct gtgtgtgtcc cagcaggcag
```

```
7895-2-6 ttatataatg ctttacaatg cttctgtgtt cctatcatcc cacaggatga ccccggtgtg gtgtgcagtg ctatgggcct gtgtgtgtcc cagcaggcag

         9999999999 9999999999 9999999999 9999999999 9999999999 9999999999 99999999
         0000000001 1111111112 2222222223 3333333334 4444444445 5555555556 66666666
         1234567890 1234567890 1234567890 1234567890 1234567890 1234567890 12345678
7895-2-3 ctctggctaa agctcagctc atgtccaacg agatccctaa -ggtggacct gaaccagcgt gtcaaccc
7895-2-4 ctctggctaa agctcagctc atgtccaacg agatccctaa aggtggacct gaaccagcgt gtcaaccc
7895-2-2 ctctggctaa agctcagctc atgtccaacg agatccctaa -ggtggacct gaaccagcgt gtcaaccc
7895-2-1 ctctggctaa agctcagctc atgtccaatg agatccctaa -ggtggacct gaaccagcgt gtcaaccc
7895-2-5 ctctggctaa agctcagctc atgtccaatg agatccctaa -ggtgaacct gaaccagcgt gtcaaccc
7895-2-6 ctctggctaa agctcagctc atgtccaacg agatccctaa -ggtggacct gaaccagcgt gtcaaccc
```

SNP LOCATIONS: 274T/G, 290G/T, 298T/C, 329T/C, 347C/A, 505C/T, 515T/G, 565T/G, 650A/G, 705G/T, 842G/A, 878T/C, 929C/T

8.

```
                1 1111111112 2222222223 3333333334 4444444445 5555555556 6666666667 7777777778 8888888889 9999999990
       1234567890 1234567890 1234567890 1234567890 1234567890 1234567890 1234567890 1234567890 1234567890 1234567890
7896-2 tcagcatcat tactccagtc ttcagtgtca catgatcctt cagaaatcat tctaatatgc tgatttgctg ttcatgttcc tgttattatc tatgttcaga
7896-3 tcagcatcat tactccagtc ttcagtgtca catgatcctt cagaaatcat tctaatatgc tgatttgctg ttcatgttcc tgttattatc tatgttcaga
7896-1 tcagcatcat tactccagtc ttcagtgtca catgatcctt cagaaatcat tctaatatgc tgatttgctg ttcatgttcc tgttattatc tatgttcaga
7896-0 tcagcatcat tactccagtc ttcagtgtca catgatcctt cagaaatcat tctaatatgc tgatttgctg ttcatgttcc tgttattatc tatgttcaga
7896-5 tcagcatcat tactccagtc ttcagtgtca catgatcctt cagaaatcat tctaatatgc tgatttgctg ttcatgttcc tgttattatc tatgttcaga
7896-4 tcagcatcat tactccagtc ttcagtgcca c-tgatcctt cagaaatcat tctaatatgc tgatttgctg ttcatgttcc tgttattatc tatgttcaga

       1111111111 1111111111 1111111111 1111111111 1111111111 1111111111 1111111111 1111111111 1111111111 1111111112
       0000000001 1111111112 2222222223 3333333334 4444444445 5555555556 6666666667 7777777778 8888888889 9999999990
       1234567890 1234567890 1234567890 1234567890 1234567890 1234567890 1234567890 1234567890 1234567890 1234567890
7896-2 cactttgtgt catgtcttag aacatgtttg atccctgtaa aaatcaccat tatacatttt tgcacatttc cattcaagtt ttatacagta gaataggagc
7896-3 cactttgtgt catgtcttag aacatgtttg atccctgtaa aaatcaccat tatacatttt tgcacatttc cattcaagtt ttatacagta gaataggagc
7896-1 cactttgtgt catgtcttag aacatgtttg atccctgtaa aaatcaccat tatacatttt tgcacatttc cattcaagtt ttatacagta gaataggagc
```

```
7896-0 cactttgtgt catgtcttag aacatgtttg atccctgtaa aaatcaccat tatacatttt tgcacatttc cattcaagtt ttatacagta gaataggagc
7896-5 cactttgtgt catgtcttag aacatgtttg atccctgtaa aaatcaccat tatacatttt tgcacatttc cattcaagtt ttatacagta gaataggagc
7896-4 cactttgtgt catgtcttag aacatgtttg atccctgtaa aagtcaacat tatacatttt tgcacatttc cattcaagtt ttatacagta gaataggagc

       2222222222 2222222222 2222222222 2222222222 2222222222 2222222222 2222222222 2222222222 2222222222 2222222223
       0000000001 1111111112 2222222223 3333333334 4444444445 5555555556 6666666667 7777777778 8888888889 9999999990
       1234567890 1234567890 1234567890 1234567890 1234567890 1234567890 1234567890 1234567890 1234567890 1234567890
7896-2 ttttaagtac tatttataac actcactcat tacattttgt aattaagaaa gaacaaattt aaacaatact gcaaatgagc aatctgttag ggctaacctc
7896-3 ttttaagtac tatttataac actcactcat tacattttgt aattaagaaa gaacaaattt aaataatact gcaaatgagc agtctgttat ggccaacctc
7896-1 ttttaagtac tatttataac actcactcat tacattttgt aattaagaaa gaacaaattt aaataatact gcaaatgagc agtctgttat ggctaacctc
7896-0 ttttaagtac tatttataac gctcactcat tacattttgt aattaagaaa gaacaaattt aaataatact gcaaatgagc agtctgttat ggctaacctc
7896-5 ttttaagtac tatttataac actcactcat tacattttgt aattaagaaa gaacaaattt aaataatact gcaaatgagc agtctgttat ggctaacctc
7896-4 ttttaagtac tatttataac actcactcat tacattttgt aattaagaaa gaacaaattt aaataatact gcaaatgagc agtctgttat ggctaacctc

       3333333333 3333333333 3333333333 3333333333 3333333333 3333333333 3333333333 3333333333 3333333333 3333333334
       0000000001 1111111112 2222222223 3333333334 4444444445 5555555556 6666666667 7777777778 8888888889 9999999990
       1234567890 1234567890 1234567890 1234567890 1234567890 1234567890 1234567890 1234567890 1234567890 1234567890
7896-2 cttcagtacc aggacacagt tcccaggtcc cacacactgc ggcagctctg caatacggcc cttattgagg tagggccctg aaaagcagcg atggttaaaa
7896-3 cttcagtacc aggacacagt tcccaggtcc cacacactgc ggcagctctg caatacggcc cttattgagg tagggccctg aaaagcagcg atggttaaaa
7896-1 cttcagtacc aggacacagt tcccaggtcc cacacactgc ggcagctctg caatacggcc cttattgagg tagggccctg aaaagcagcg atggttaaaa
7896-0 cttcagcacc aggacacagt tcccaggtcc cacacactgc ggcagctctg caatacggcc cttattgagg tagggccttg aaaagcagcg atggttaaaa
7896-5 cttcagcacc aggacacagt tcccaggtcc cacacactgc ggcagctctg caatacggcc cttattgagg tagggccctg aaaagcagcg atggttaaaa
7896-4 cttcagcacc aggacacagt tcccaggtcc cacacactgc ggcaactctg caatacggcc cttattgagg tagggccctg aaaagcagcg atggttaaaa

       4444444444 4444444444 4444444444 4444444444 44444444
       0000000001 1111111112 2222222223 3333333334 44444444
       1234567890 1234567890 1234567890 1234567890 12345678
7896-2 tagatcttgg ggcaacagta cttcccattc gcttgacctg ttggtgac
7896-3 tagatcttgg ggcaacagta cttcccattc gcttgacctg ttggtgac
7896-1 tagatcttgg ggcaacagta cttcccattt gcttgacctg ttggtgac
```

```
7896-0 tagatcttgg ggcaacagta cttcccattc gcttgacctg ttggtgac
7896-5 tagatcttgg ggcaacagta cttcccattc gcttgacctg ttggtgac
7896-4 tagatcttgg ggcaacagta cttcccattc gcttgacctg ttggtgac
```

SNP LOCATIONS: 307C/T

9.

```
                1 1111111112 2222222223 3333333334 4444444445 5555555556 6666666667 7777777778 8888888889 9999999990
       1234567890 1234567890 1234567890 1234567890 1234567890 1234567890 1234567890 1234567890 1234567890 1234567890
7898-1 gggtggagtg tctcttggaa aagtcaggat ggcatgactg cttaaaagaa caaataaaaa aagaaaagca cttttgccat tttacttctg ggcagggatc
7898-6 gggtggagtg tctcttggaa aagtcaggat ggcatgactg cttaaaagaa caaataaaaa aagaaaagca cttttgccat tttacttctg ggcagggatc
7898-4 gggtggagtg tctcttggaa aagtcaggat ggcatgactg cttaaaagaa caaataaaaa aagaaaagca cttttgccat tttacttctg ggcagggatc
7898-2 gggtggagtg tctcttggaa aagtcaggat ggcatgactg cttaaaagaa caaataaaaa aagaaaagca cttttgccat tttacttctg ggcagggatc
7898-3 gggtggagtg tctcttggaa aagtcaatat ggcatgactg catgaaagaa caaataaaaa a-gaaaagaa cttttactat tttacttctg ggcagggatc
7898-5 gggtggagtg tctcttggaa aagtcaatat ggcatgactg catgaaagaa caaataaaaa a-gaaaagaa cttttactat tttacttctg ggcagggatc

       1111111111 1111111111 1111111111 1111111111 1111111111 1111111111 1111111111 1111111111 1111111111 1111111112
       0000000001 1111111112 2222222223 3333333334 4444444445 5555555556 6666666667 7777777778 8888888889 9999999990
       1234567890 1234567890 1234567890 1234567890 1234567890 1234567890 1234567890 1234567890 1234567890 1234567890
7898-1 atgtcatcga tggactcgcc cttctccagt ttcttttcca tttcaatcat gagcttcact ccatcgacca ccagctgcac ctgctgcacc tcggaggagc
7898-6 atgtcatcga tggactcgcc cttttccagt ttcttttcca tttcaatcat gagcttcaca ccatcgacca ccagctgcac ctgctgcacc tcggaggagc
7898-4 atgtcatcga tggactcgcc cttctccagt ttcttttcca tttcaatcat gagcttcaca ccatcgacca ccagctgcac ctgctgcacc tcggaggagc
7898-2 atgtcatcaa tggactcgcc cttctccagt ttcttttcca tttcagtcat gagcttcaca ccatcgacca ccagctgcac ctgctgcacc tcggaggagc
7898-3 atgtcatcaa tggactcgcc cttctctagt ttcttttcca tttcaaccat gagcttcaca ccatcaacca ccagctgtac ctgctgcacc tcggaggagc
7898-5 atgtcatcaa tggactcgcc cttctctagt ttcttttcca tttcaaccat gagcttcaca ccatcaacca ccagctgtac ctgctgcacc tcggaggagc

       2222222222 2222222222 2222222222 22222222
       0000000001 1111111112 2222222223 33333333
       1234567890 1234567890 1234567890 12345678
7898-1 ccagacgatc agcgttggag atgtcgaaca caccacca
```

```
7898-6 ccagacgatc agcgttggag atgtcgaaca caccacca
7898-4 ccagacggtc agcgttggag atgtcgaaca caccacca
7898-2 ccagacgatc agcgttggag atgtcgaaca caccacca
7898-3 ccagacgatc agcgttggag atgtcgaaca caccacca
7898-5 ccagacggtc agcgttggag atgtcgaaca caccacca
```

SNP LOCATIONS: 27G/A, 28G/T, 42T/A, 44A/G, 69C/A, 76G/A, 78C/T, 109G/A, 127C/T, 147T/C, 166G/A, 178C/T, 208A/G

10.

```
                1 1111111112 2222222223 3333333334 4444444445 5555555556 6666666667 7777777778 8888888889 9999999990
       1234567890 1234567890 1234567890 1234567890 1234567890 1234567890 1234567890 1234567890 1234567890 1234567890
7909-2 tcgctttagt ctccaccatg tcaccaaata attcatactt aaagacttaa gttatttatt ttaatctaac ctaaaacatg gcctacctat atattaactg
7909-5 tcgctttagt ctccaccatg tcaccaaata attcatactt aaagacttaa gttatttatt tttatctaac ctaaaacatg gcctacctat atattaactg
7909-0 tcgctttagt ctccaccatg tcaccaaata attcatactt aaaggcttaa gttatttatt ttaatctaac ctaaaacatg gcctacctat atattaactg
7909-1 tcgctttagt ctccaccatg tcaccaaata attcatactt aaagacttaa gttatttatt ttaatctaac ctaaaacatg gcctacctat atattgactg
7909-3 tcgctttagt ctccaccatg tcaccaaata attcatactt aaagacttaa gttatttatt ttaatctaac ctaaaacatg gcctacctat atattgactg
7909-4 tcgctttagt ctccaccatg tcaccaaata attcatactt aaagacttaa gttatttatt ttaatctaac ctaaaacatg gcctacctaa atattgactg

       1111111111 1111111111 1111111111 1111111111 1111111111 1111111111 1111111111 1111111111 1111111111 1111111112
       0000000001 1111111112 2222222223 3333333334 4444444445 5555555556 6666666667 7777777778 8888888889 9999999990
       1234567890 1234567890 1234567890 1234567890 1234567890 1234567890 1234567890 1234567890 1234567890 1234567890
7909-2 attcaacatt atttttcggt aacatttaac aggttacgtt agttaacatg aaataatagt gtctggattt attaatctta gttcatgtta atttcaactt
7909-5 attcaatatt atttttcggt a-catttaac aggttacata agttaacatg aaataatagt gtctggattt attaatctta gttcatgtta atttcaactt
7909-0 attcaatatt atttttcggt aacatttaac aggttacgta agttaacatg aaataatagt gtctggattt attaatctta gttcatgtta atttcaactt
7909-1 attcaatatt attttttggt aacatttaac aggttacata agttaacatg aaataatagt gtctggattt attaatctta gttcatgtta atttcaactt
7909-3 attcaatatt atttttcggt aacatttaac aggttacata agttaacatg aaataacagt gtctgcattt attaatctta gttcatgtta atttcaactt
7909-4 attcaacatt atttttcggt aacatttaac aggttacatt agttaacatg aaataacagt gtctgcattt atta-tctta gttcatgtta atttcaactt

       2222222222 2222222222 2222222222 2222222222 2222222222 2222222222 2222222222 2222222222 2222222222 2222222223
       0000000001 1111111112 2222222223 3333333334 4444444445 5555555556 6666666667 7777777778 8888888889 9999999990
```

```
       1234567890 1234567890 1234567890 1234567890 1234567890 1234567890 1234567890 1234567890 1234567890 1234567890
7909-2 ttaataatcc tttataaaat caacagttgt gtctgctaat atattattta atggagctaa catgaactaa ca-----tag ttgtctttgt attaacaaac
7909-5 ttaataatcc tttataaaat caacagttgt gtctgctaat atattattta atggagctaa catgaactaa caataaatag ttgtctttgc attaacaaac
7909-0 ttaataatcc tttataaaat caacagttgt gtctgctaat atattattta atggagctaa catgaactaa caataaatag ttgtctttgt attaacaaac
7909-1 ttaataatcc tttataaaat caacagttgt gtctgctaat atattattta atggagctaa catgaactaa caataaatag ttgtctatgt attaacaaac
7909-3 ttaataatcc tttataaaat caacagttgt gtctgctaat atattattta atggagctaa catgaactaa caataaatag ttgtctatgt attaacaaac
7909-4 ttaataatcc tttataaaat caacagttgt gtctgctaat at--tattta atggagctaa catgaactaa caataaatag ttgtctttgt attaacaaac

       3333333333 3333333333 3333333333 3333333333 3333333333 3333333333 3333333333 3333333333 3333333333 3333333334
       0000000001 1111111112 2222222223 3333333334 4444444445 5555555556 6666666667 7777777778 8888888889 9999999990
       1234567890 1234567890 1234567890 1234567890 1234567890 1234567890 1234567890 1234567890 1234567890 1234567890
7909-2 attaacagat aatattaact actgcaccaa ttagcaattc cacactgtta gttaatgtaa acctgcttta actaagggtg tattcacacc tgtcttgttt
7909-5 gttaacaaat aatattaact actgcaccaa ttgacaattc cacactgtta gttaatgtaa acctgcttta actaagggtg tattcacacc tgtcttgttt
7909-0 attaacaaat aatattaact actgcaccaa ttagcaattc cacactgtta gttaatgtta acctgcttta actaagggtg tattcacacc tgtcttgttt
7909-1 gttaacaaat aatattaact actgcaccaa ttagcaattc cacactgtta gttaatgtta acctgcttta actaagggtg tattcacacc tgtcttgatt
7909-3 attaacaaat aatattaact actgcaccaa ttagcaattc cacactgtta gttaatgtta acctgcttca actaagggtg tattcacacc tgtcttgttt
7909-4 attaacaaat aatattaact aatgcaccaa ttagcaattg tacactgtta cttaatgtta acctgcttta actaagggtg tattcacacc tgtcttgttt

       4444444444 4444444444 4444444444 4444444444 4444444444 4444444444 4444444444 4444444444 4444444444 4444444445
       0000000001 1111111112 2222222223 3333333334 4444444445 5555555556 6666666667 7777777778 8888888889 9999999990
       1234567890 1234567890 1234567890 1234567890 1234567890 1234567890 1234567890 1234567890 1234567890 1234567890
7909-2 ggtaagactg aatcaaactt aagttaattt cccctcttgc tgcggatctt ttgggtaggt gtgaataaaa caaa-tcgca tttgggtgcg gaccaaacaa
7909-5 ggtaagactg aatcaaattt aagttaattt cccctcttgc tgcggatctt ttgggtaggt gtgaataaaa caaa-tcgca tttgggtgcg gaccaaacaa
7909-0 ggtaagactg aatcaaattt aagttaatct cccctcttgc tgcggatctt ttgggtaggt gtgaatataa caaa-tcgca tttgggtgcg gaccaaacaa
7909-1 ggtaagactg aatcaaattt aagttaatct cccctcttgc tgcggatctt ttgggtaggt ttgaatataa caaa-tcgca tttgggtgcg gaccaaacaa
7909-3 ggtaagactg aatcaaattt aagttaattt cccctcttgc tgtggatctt ttgggtaggt gtgaatataa caaa-tcgca cttgggtgcg gaccaaacaa
7909-4 ggtcagac-- aatcaaactc aagttaattt cccctcttgc tgcggatctt ttgggtaggt gtgaatgtaa caaaatcgca ctcgggtgca gaccaaacaa

       5555555555 5555555555 5555555555 5555
       0000000001 1111111112 2222222223 3333
```

```
       1234567890 1234567890 1234567890 1234
7909-2 ccgtactgag acccggctga agaggtggtc tcgg
7909-5 ccgtactgag acccggctga agaggtggtc tcgg
7909-0 ccgtactgag acccggctga agaggtggtc tcgg
7909-1 ccgtactgag acccggctga agaggtggtc tcgg
7909-3 ccgtactgag acccggctga agaggtggtc tcgg
7909-4 ccgtactgag acccggctga agaggtggtc tcgg
```

SNP LOCATIONS: 96A/G, 10T/C, 138A/G, 140A/T, 157T/C, 166G/C, 287T/A, 301A/G, 359T/A, 418T/C, 429T/C, 468T/A, 481T/C

11.

```
                1 1111111112 2222222223 3333333334 4444444445 5555555556 6666666667 7777777778 8888888889 9999999990
       1234567890 1234567890 1234567890 1234567890 1234567890 1234567890 1234567890 1234567890 1234567890 1234567890
7918-0 caagaagaag attgagcacc ataatgcagg aaaccacaag ttttcaatgg gactgaatcg gttttcagac atgaccttta ctgaatttaa aaagtcctac
7918-3 caagaagaag attgagcacc ataatgcagg aaaccacaag ttttcaa-gg gactgaatcg gttttcagac atgaccttta ctgaatttaa aaagtcctac
7918-1 caagaagaag attgagcacc ataatgcagg aaaccacaag tttgcaa-gg gactgaatcg gttttcagac atgaccttta ctgaatttaa aaagtcctac
7918-2 caagaagaag attgagcacc ataatgcagg gaaccacaag ttttcaa-gg gactgaatcg gttttcagac atgaccttta ctgaatttaa aaagtcctac
7918-4 caagaagaag attgagcacc ataatgcagg aaaccacaag ttttcaa-gg gactgaatcg gttttcagac atgaccttta ctgaatttaa aaagtcctac
7918-5 caagaagaag attgagcacc ataatgcagg aaaccataag ttttcaa-gg gactgtatca gttttcagac atgacatttg ctgaatttaa aaagtcctac

       1111111111 1111111111 1111111111 1111111111 1111111111 1111111111 1111111111 1111111111 1111111111 1111111112
       0000000001 1111111112 2222222223 3333333334 4444444445 5555555556 6666666667 7777777778 8888888889 9999999990
       1234567890 1234567890 1234567890 1234567890 1234567890 1234567890 1234567890 1234567890 1234567890 1234567890
7918-0 ctcctgacag aacctcag-a actgctccgc cactagaggg aaccatgtga gcagtaatgg gccttatcct gatatgattg actggagaac gaaaggacac
7918-3 ctcctgacag aacctcagga actgctccgc cactagaggg aaccatgtga gcagtaatgg gccttatcct gatatgattg actggagaac gaaaggaaac
7918-1 ctcctgacag aacctcagga actgctccgc cactagaggg aaccatgtga gcagtaatgg gccttatcct gatacgattg actggagaac gaaaggacac
7918-2 ctcctgacag aacctcagga actgctccgc cactagaggg aaccatgtga gcagtaatgg gccttatcct gatacgattg actggagaac gaaaggacac
7918-4 ctcctgacag aacctcagga actgctccgc cactagaggg aaccatgtga gcagtaatgg gccttatcct gatacgattg actggagaac gaaaggacac
7918-5 ctcctgacag aacctcagga actgctccgc cactagaggg aaccatctga gcagtaatgg gccttatcct gatacgattg actggagaac gaaaggaaac
```

```
       2222222222 2222222222 2222222222 2222222222 2222222222 2222222222 2222222222 222222222
       0000000001 1111111112 2222222223 3333333334 4444444445 5555555556 6666666667 777777777
       1234567890 1234567890 1234567890 1234567890 1234567890 1234567890 1234567890 123456789
7918-0 tatgtaactg atgtcaagaa ccagggagct tgtggtagct gctggacttt ttccaccaca ggctgtctgg agtctgtca
7918-3 tatgtaactg atgtcaagaa ccagggagct tgtggtagct gctggacttt ttccaccaca ggctgtctgg agtctgtca
7918-1 tatgtaactg atgtcaagaa ccagggagct tgtggtagct gctggacttt ttccaccaca ggctgtctgg agtctgtca
7918-2 tatgtaactg atgtcaagaa ccagggagct tgtggtagct gctggacttt ttccaccaca ggctgtctgg agtctgtca
7918-4 tatgtaactg atgtcaagaa ccagggagct tgtggtagct gctggacttt ttccaccaca ggctgtctgg agtctgtca
7918-5 tatgtaacta atgtcaagaa ccagggacct tgtggtagct gctggacttt ttccaccaca ggctgtctgg agtctgtca
```

SNP LOCATIONS: 175C/T, 198C/A

12.

```
                 1 1111111112 2222222223 3333333334 4444444445 5555555556 6666666667 7777777778 8888888889 9999999990
        1234567890 1234567890 1234567890 1234567890 1234567890 1234567890 1234567890 1234567890 1234567890 1234567890
7921-1  ctggcttctg ctcacgctgg gactagttgc ggctcaagca ggtaaagtct gaacagatgt ttggtttttg gagggaaatc tagaaatttg gaaaacagca
7921-7  ctggcttctg ctcacgctgg gactagttgc ggctcaagca ggtaaagtct gaacagatgt ttggtttttg gagggaaatc tagaaatttg gaaaacagca
7921-3  ctggcttctg ctcacgctgg gactagttgc ggctcaagca ggtaaagtct gaacagatgt ttggtttttg gagggaaatc tagaaatttg gaaaacagca
7921-4  ctggcttctg ctcacgctgg gactagttgc ggctcaagca ggtaaagtct gaacagatgt ttggtttttg gagggaaatc tagaaatttg gaaaacagca
7921-5  ctggcttctg ctcacgctgg gactagttgc ggctcaagca ggtaaagtct gaacagatgt ttggtttttg gagggaaatc tagaagtttg gaaaacagca
7921-2  ctggcttctg ctcacgctgg gactagttgc ggctcaagca ggtaaagtct gaacagatgt ttggtttttg gagggaaatc tagaagtttg gaaaacagca
7921-6  ctggcttctg ctcacgctgg gactagttgc ggctcaagca ggtaaagtct gaacagatgt ttggtttttg gagggaaatt tagaaatttg gaaaacagca
7921-0  ctggcttctg ctcacgctgg gactagttgc ggctcaagca gagaaa---- ---------- ---------- ---------- ---------- ----------

        1111111111 1111111111 1111111111 1111111111 1111111111 1111111111 1111111111 1111111111 1111111111 1111111112
        0000000001 1111111112 2222222223 3333333334 4444444445 5555555556 6666666667 7777777778 8888888889 9999999990
        1234567890 1234567890 1234567890 1234567890 1234567890 1234567890 1234567890 1234567890 1234567890 1234567890
7921-1  taacttgagt atattatagt aagagccagt gctcaattgc aatagtttta tatattctaa cgtacagttt ttgtattatg gcgcatgttt tt-gtagaga
7921-7  taacttgagt atattatagt aagagccagt gctcaattgc aatagtttta tatattctaa cgtacagttt ttgtattatg gcgcatgttt tt-gtagaga
7921-3  taacttgagt atattatagt aagacccagt gctcaattgc aatagtttta tatattctaa cgtacagttt ttgtattatg gcgcatgttt tt-gtagaga
7921-4  taacttgagt atattatagt aagacccagt gctcaattgc aatagtttta tatattctaa cgtacagttt ttgtattatg gcgcatgttt tttgtagaga
7921-5  taacttgagt atattatagt aagacccagt gctcaattgc aatagtttta tatattctaa cgtacagttt ttgtattatg gcgcatgttt tt-gtagaga
7921-2  taacttgagt atattatagt aagagccagt gctcaattgc aatagtttta tatattctaa cgtacacttt ttgtattatg gcgcatgttt tt-gtagaga
7921-6  taacttgagt atattatagt aagagccagt gctcaattgc aatagtttta tatattctaa cgtacagttt ttgtattatg gcgcatgttt tt-gtagaga
7921-0  ---------- ---------- ---------- ---------- ---------- ---------- ---------- ---------- ---------- ----------

        2222222222 2222222222 2222222222 2222222222 2222222222 2222222222 2222222222 2222222222 2222222222 2222222223
        0000000001 1111111112 2222222223 3333333334 4444444445 5555555556 6666666667 7777777778 8888888889 9999999990
        1234567890 1234567890 1234567890 1234567890 1234567890 1234567890 1234567890 1234567890 1234567890 1234567890
7921-1  aagcagagca agtgctgcca aactggctta cgggaatcat tgccgttggt gtgttcctct ttctgatctt cgtcaccttc cttgtaaaca aagcctggtg
7921-7  aagcagagca agtgctgcca aactggctta cgggaatcat tgccgttggt gtgttcctct ttctgatctt cgtcaccttc cttgtaaaca aagcctggtg
7921-3  aagcagagca agtgctgcca aactggctta cgggaatcat tgccgttggt gtgttcttct ttctgatctt cgtcaccttc cttgtaaaca aagcctggtg
```

```
7921-4 aagcagagca agtgctgcca aactggctta cgggaatcat tgccgttggt gtgttcttct ttctgatctt cgtcaccttc cttgtaaaca aagcctggtg
7921-5 aagcagagca agtgctgcca aactggctta cgggaatcat tgccgttggt gtgttcttct ttctgatctt cgtcaccttc cttgtaaaca aagcctggtg
7921-2 aagcagagca agtgctgcca aactggctta cgggaatcat tgccgttggt gtgttcctct ttctgatctt cgtcgccttc cttgtaaaca aagcctggtg
7921-6 aagcagagca agtgctgcca aactggctta cgggaatcat tgccgttggt gtgttcctct ttctgatctt cgtcaccttc cttgtaaaca aagcctggtg
7921-0 ---cagagca agtgctgcca aactggctta cgggaatcat tgccgttggt gtgttcctct ttctgatctt cgtcaccttc cttgtaaaca aagcctggtg

       3333333333 3333333333
       0000000001 1111111112
       1234567890 1234567890
7921-1 tgaaactcca agcaaaccag
7921-7 tgaaactcca agcaaaccag
7921-3 tgaaactcca agcaaaccag
7921-4 tgaaactcca aacaaaccag
7921-5 tgaaactcca agcaaaccag
7921-2 tgaaactcca agcaaaccag
7921-6 tgaaactcca agcaaaccag
7921-0 tgaaactcca agcaaaccag
```

SNP LOCATIONS: 86A/G, 125G/C, 257C/T

13.

```
                1 1111111112 2222222223 3333333334 4444444445 5555555556 6666666667 7777777778 8888888889 9999999990
       1234567890 1234567890 1234567890 1234567890 1234567890 1234567890 1234567890 1234567890 1234567890 1234567890
7927-1 aagccgtggg acaaatctca gtcgacccgt acctagtgcc caccttccag ctcacactcg tgctgaggct cgactccagg ggcttttggc caaaaatcca
7927-4 aagccgtggg acaaatctca gtcgacccgt acctggtgcc caccttccag ctcacactcg tcctgaggct cgactccagg ggcttttggc caaaaatcca
7927-2 aagccgtggg acaaatctca ggcgacccgt acctagtgcc caccttccag ctcacactcg tgctgaggct cgactccagg ggcttttggc caaaaatcca
7927-3 aagccgtggg acaaatctca gtcgacccgt acctagtgcc caccttccag ctcacactcg tgctgaggct cgactccagg ggcttttggc caaaaatcca
7927-0 aagccgtggg acaaatctca gtcgacccgt acctagtgcc caccttccag ctcacactcg tgctgaggct cgactccagg ggcttttggc caaaaatcca
7927-5 aagccgtggg acaaatctca gtcgacccgt acctagtgcc caccttccag ctcacactcg tgctgaggct cgactccagg ggcttttggc caaaaatcca

       1111111111 1111111111 1111111111 1111111111 1111111111 1111111111 1111111111 1111111111 1111111111 1111111112
       0000000001 1111111112 2222222223 3333333334 4444444445 5555555556 6666666667 7777777778 8888888889 9999999990
       1234567890 1234567890 1234567890 1234567890 1234567890 1234567890 1234567890 1234567890 1234567890 1234567890
7927-1 ggggcttttc gccggcagat cgccttcgtc tccagttgtt agacgtgcac ttaaactgag cactggattc agagttatta agagaaaact gtacagctct
7927-4 ggggcttttc gccggtagat cgccttcgtc tccagttgtt agacgtgcac ttaaactgag cactggattc agagttatta agagaaaact gtacagctct
7927-2 ggggcttttc gccggcagat cgccttcgtc tccagttgtt ggacgtgcac ttaaactgag cactggattc agagttatta agagaaaact gtacagctct
7927-3 ggggcttttc gccggcagat cgccttcgtc tccagttgtt agacgtgcac ttaaactgag cactggattt agagttatta agagaaaact gtacagctct
7927-0 ggggcttttc gccggcagat cgccttcgtc tccggttgtt agacgtgcac ttaaactgag cactggattc agagttatta agagaaaact gtacagctct
7927-5 ggggcttttc gccggcagat caccttcgtc tccggttgtt agacgtgcac ttaaactgag cactggattc agagttatta agagaaaact gtacagctct

       2222222222 2222222222 2222222222 2222222222 2222222222 2222222222 2222222222 2222222222 2222222222 2222222223
       0000000001 1111111112 2222222223 3333333334 4444444445 5555555556 6666666667 7777777778 8888888889 9999999990
       1234567890 1234567890 1234567890 1234567890 1234567890 1234567890 1234567890 1234567890 1234567890 1234567890
7927-1 gaagagcttt tgattgaaga atgctgagct ggactgattg aatgtccatg tcatggattt taccaggttt ttaaatgaag ccgttcggtc tcacaagagg
7927-4 gaagagcttt tgattgaaga atgctgagct cgactgattg aatgtccatg tcatggattt taccaggttt ttaaatgaag ccgttcggtc tcacaagagg
7927-2 gaagagcttt tgattgaaga atgctgagct cgactgattg aatgtccatg tcatggattt taccaggttt ttaaatgaag ccgttcggtc tcacaggagg
7927-3 gaagagcttt tgattgaaga atgctgagct cgactgattg aatgtccatg tcatggattt taccaggttt ttaaatgaag ccgttcggtc tcacaagagg
7927-0 gaagagcttt tgattgaaga atgctgagct cgactgattg aatgtccatg tcatggattt taccaggttt ttaaatgaag ctgttcggtc tcacaagagg
7927-5 gaagagcttt tgattgaaga atgctgagct cgactgattg aatgtccatg tcatggattt taccaggttt ttaaatgaag ctgttcggtc tcacaagagg
```

```
       3333333333 3333333333 33
       0000000001 1111111112 22
       1234567890 1234567890 12
7927-1 cctgaagtgt tgtatgactg tt
7927-4 cctgaagtgt tgtatgactg tt
7927-2 cctgaagtgt tgtatgactg tt
7927-3 cctgaagtgt tgtatgactg tt
7927-0 cctgaagtgt tgtatgactg tt
7927-5 cctgaagtgt tgtatgactg tt
```

SNP LOCATIONS: 134A/G, 282C/T

14.

```
                1 1111111112 2222222223 3333333334 4444444445 5555555556 6666666667 7777777778 8888888889 9999999990
       1234567890 1234567890 1234567890 1234567890 1234567890 1234567890 1234567890 1234567890 1234567890 1234567890
7929-2 accaggctct tcatgtcaac actgacaagt ggattgctga aaatgagaat tcatttctac caccggtatg caacaaatta atgtaagtga gttaaactca
7929-6 accaggctct tcatgtcaac actgacaagt ggattgctga aaatgagaat tcatttctac caccggtatg caacaaatta atgtaagtga gttaaactta
7929-3 accaggctct tcatgtcaac actgacaagt ggattgctga aaatgagaat tcatttctac cacctgtatg caacaaatta atgtaagtga gttaaactta
7929-4 accaggctct tcatgtcaac actgacaagt ggattgctga aaatgagaat tcatttctac cacctgtatg caacaaatta atgtaagtga gttaaactta
7929-1 accaggctct tcatgtcaac actgacaagt ggattgctga aaatgagaat tcatttctac caccagtatg caacaaatta atgtaagtga gttaaactta
7929-5 accaggctct tcatgtcaac actgacaagt ggattgctga aaatgagaat tcatttctac cacctgtatg caacaaatta atgtaagtga gttaaactta

       1111111111 1111111111 1111111111 1111111111 1111111111 1111111111 1111111111 1111111111 1111111111 1111111112
       0000000001 1111111112 2222222223 3333333334 4444444445 5555555556 6666666667 7777777778 8888888889 9999999990
       1234567890 1234567890 1234567890 1234567890 1234567890 1234567890 1234567890 1234567890 1234567890 1234567890
7929-2 tctttcataa acaaacaaaa acatgcctta tataatcaca ttgataaaaa tacaaaatat cataagcctt atctttacat tgtgttttag gttcttttat
7929-6 tctttcatga acaaacaaaa acatgcctta tataatcaca ttgataaaaa tacaaaatat cataagcctt atctttacat tgtgttttag gttcttttat
7929-3 tctttcataa acaaacaaaa acatgcctta tataatcaca ttgataaaaa tacaaaatat cataagcctt atctttacat tgtgttttag gttcttttat
7929-4 tctttcataa acaaacaaaa acatgcctta tataatcaca ttgataaaaa tacaaaatat cataagcctt atctttacat tgtgttttag gttcttttat
7929-1 tctttcataa acaaacaaaa acatgcctta tataatcaca ttgataaaaa tacaaaatat cataggcctt atctttacat tgtgttttag gttcttttat
```

```
7929-5 tctttcataa acaaacaaaa acatgcctta taaaatcaca ttgataaaaa tacaaaatat cataagcctt atctttacat tgtgttttag gttcttttat

       2222222222 2222222222 2222222222 2222222222 2222222222 2222222222 2222222222 2222222222 2222222222 2222222223
       0000000001 1111111112 2222222223 3333333334 4444444445 5555555556 6666666667 7777777778 8888888889 9999999990
       1234567890 1234567890 1234567890 1234567890 1234567890 1234567890 1234567890 1234567890 1234567890 1234567890
7929-2 caactgaata ttatgtatgt tggaggtcca aatgcaagga aggattatca cattgaggaa ggggaagagg tgaggtgcta ttcatgtaac aaattagata
7929-6 caactgaata ttatgtatgt tggaggtcca aatgcaagga aggattatca cattgaggaa ggggaagagg tgaggtgcta ttcatgtaac aaatgacata
7929-3 caactgaata ttatgtatgt tggaggtcca aatgcaagga aggattatca cattgaggaa ggggaagagg tgaggtgcta ttcatgtaac aaatgagata
7929-4 caactgaata ttatgtatgt tggaggtcca aatgcaagga aggattatca cattgaggaa ggggaagagg tgaggtgcta ttcatgtaac aaatgagata
7929-1 caactgaata ttatgtatgt tggaggtcca aatgcaagga aggattatca cattgaggaa ggggaagagg tgaggtgcta ttcatgtaac aaattagata
7929-5 caactgaata ttatgtatgt tggaggtcca aatgcaagga aggattatca cattgaggaa ggggaagagg tgaggtgcta ttcatgtaac aaattagata

       3333333333 3333333333 3333333333 3333333333 3333333333 3333333333 3333333333 3333333333 3333333333 3333333334
       0000000001 1111111112 2222222223 3333333334 4444444445 5555555556 6666666667 7777777778 8888888889 9999999990
       1234567890 1234567890 1234567890 1234567890 1234567890 1234567890 1234567890 1234567890 1234567890 1234567890
7929-2 atattacaaa ttaacatgac atgttcattt aggaatagct gacttgcttt tttattttga ttaactgcac tgtattctgg gaattagacc cgagaaggct
7929-6 atattacaaa ttaacatgac atgttcattt aggaatagct gacttgcttt tttattttga ttaactgcac tgtattctgg gaattagacc cgagaaggct
7929-3 atattacaaa ttaacatgac atgttcattt aggaatagct gacttgcttt tttactttga ttaactgcac tgtattctgg gaattagacc cgagaaggct
7929-4 atattacaaa ttaacacgac atgttcattt aggaatagct gacttgcttt tttattttga ttaactgcac tgtattctgg gaattagacc cgagaaggct
7929-1 atattacaaa ttaacatgac atgttcattt aggaatagct gacttgcttt tttattttga ttaactgcac tgtattctgg gaattagacc cgagaaggct
7929-5 atattacaaa ttaacatgac atgttcattt aggaatagct gacttgcttt tttattttga ttaactgcac tgtattctgg gaattagacc cgagaaggct

       4444444444 4444444444 4444444444 4444444444 4444444444 4444444444 4444444444 4444444444 4444444444 4444444445
       0000000001 1111111112 2222222223 3333333334 4444444445 5555555556 6666666667 7777777778 8888888889 9999999990
       1234567890 1234567890 1234567890 1234567890 1234567890 1234567890 1234567890 1234567890 1234567890 1234567890
7929-2 aacaggtgat aagaacagaw ttagccatga tcttcgcaaa aggttagtgc tttagactca gcagctgtgg gattagattt accaggaaat gaagaggaaa
7929-6 aacaggtgat aagaacagaa ttagccatga tcttcacaaa aggttagtgc tt-agactca gcagctgtgg gattagattt accaggaaac gaagaggaaa
7929-3 aacaggtgat aagaacaga- ttagccatga tcttcacaaa aggttagtgc tttagactca gcagctgtgg gattacattt accaggaaac gaagaggaaa
7929-4 aacaggtgat aagaacagaa ttagccatga tcttcacaaa aggttagtgc tttagactca gcagctgtgg gattacattt accaggaaac gaagaggaaa
7929-1 aacaggtgat aagaacagaa ttagccatga tcttcacaaa aggttagtgc tttagactca gcagctgtgg gattagattt accaggaaat gaagaggaaa
```

```
7929-5 aacaggtgat aagaacagaa ttagccatga tcttcacaaa aggttagtgc tttagactca gcagctgtgg gattagattt accaggaaat gaagaggaaa

       5555555555 5555555555 5555555555 5555555555 5555555555 5555555555 5555555555 5555555555 5555555555 5555555556
       0000000001 1111111112 2222222223 3333333334 4444444445 5555555556 6666666667 7777777778 8888888889 9999999990
       1234567890 1234567890 1234567890 1234567890 1234567890 1234567890 1234567890 1234567890 1234567890 1234567890
7929-2 aggacaagag tgcataacta attattaaag atgatctgat attgacagca tgattttcta cacstgcatt gaactttgaa atttgcacca cacaccgttc
7929-6 aggacaagag tgcataacta attattaaag atgatctgat attgacagca tgattttcta cacstgcatt gaactttggg atttgcacca cacaccgttc
7929-3 agtacaagag tgcataacta attattaaag atgatctgat attgacagca tgattttcta cacctgcatt gaactttggg atttgcacca cacaccgttc
7929-4 aggacaagag tgcataacta attattaaag atgatctgat attgacagca tgattttcta cacctgcatt gaactttggg atttgcacca cacaccgttc
7929-1 aggacaagag tgcataacta attattaaag atgatctgat attgacagca tgattttcta cacctgcatt gaactttggg atttgcacca caaaccgaat
7929-5 aggacaagag tgcataacta attattaaag atgatccgat attgacagca tgattttcta cacctgcatt gaactttggg atttgcacca caaaccgaat

       6666666666 6666666666 6666666666 6666666666 6666666666 6666666666 6666666666 6666666666 6666666666 6666666667
       0000000001 1111111112 2222222223 3333333334 4444444445 5555555556 6666666667 7777777778 8888888889 9999999990
       1234567890 1234567890 1234567890 1234567890 1234567890 1234567890 1234567890 1234567890 1234567890 1234567890
7929-2 a--ctacatt tcactacatg gactgcatag ttttccaaat gcactttgca tttttaatgg ggacaaagtc tttgtgaaaa gtttttgat tttcaattta
7929-6 a--ctacatt tcactacatg gactgcatag ttttccaaat gcactttgca tttttaatgg ggacaaagtc tttgtgaaaa gtttttgat tttcaattta
7929-3 a--ctacatt tcactacatg gactgcatag ttttccaaat gcactttgca tttttaatgg ggacaaagtc tttgtgaaaa gttttktgat tttcaattta
7929-4 a--ctacatt tcactacatg gactgcatag ttttccaaat gcactttgca tttttaatgg ggacaaagtc tttgtgaaaa gtttttgat tttcaattta
7929-1 ggcttacatt tctctacatg gactgcatag ttttccaaat gcactttgca tttttaatgg ggacaaagtc tttgtgaaaa gtttttgat tttcaattta
7929-5 ggcttacatt tctctacatg gactgcatag ttttccaaat gcactttgca tttttaatgg ggacaaagtc tttgtgaaaa gtttttgat tttcaattta

       7777777777 7777777777 7777777777 7777777777 7777777777 7777777777 7777777777 7777777777 7777777777 7777777778
       0000000001 1111111112 2222222223 3333333334 4444444445 5555555556 6666666667 7777777778 8888888889 9999999990
       1234567890 1234567890 1234567890 1234567890 1234567890 1234567890 1234567890 1234567890 1234567890 1234567890
7929-2 attaaatttt ttagctgcat tttattttt t-----agct gcatttctgc atggcattaa aaatcattta taaagcacct ttacagtggc aaatttccaa
7929-6 attaaatttt ttagctgcat tttattttt ------agct gcatttctgc atggcattaa aaatcattta taaagcacct ttacagtggc aaattcccaa
7929-3 attaaatttt ttagctgcat tttattttt ------agct gcatttctgc atggcattaa aaatcattta taaagcacct ttacagtggc aaatttccaa
7929-4 attaaatttt ttagctgcat tttattttt ------agct gcatttctgc atggcattaa aaatcattta taaagcacct ttacagtggc aaatttccaa
7929-1 attacatttt t-agctgcat tttattttt tcaaatagct gcatttctgc atggcattaa aaagcattta taaagcacct ttacagtggc aaatttccaa
```

```
7929-5 attacatttt t-agctgcat ttcatttttt -caaatagct gcatttctgc atggcattaa aaagcattta taaagcacct ttacagtggc aaatttccaa

       8888888888 8888888888 8888888888 8888888888 8888888888 8888888888 8888888888 8888888888 8888888888 8888888889
       0000000001 1111111112 2222222223 3333333334 4444444445 5555555556 6666666667 7777777778 8888888889 9999999990
       1234567890 1234567890 1234567890 1234567890 1234567890 1234567890 1234567890 1234567890 1234567890 1234567890
7929-2 atcaaacaac agcaggaaat taatagaatt gactcttgct gagcatttca caataataca cctttaaaaa aaaa---tga cctagtttgg tttagtgagc
7929-6 atcaaacaac agcaggaaat taatagaatt gactcttgct gagcatttca caataataca cctttaaaaa aaaa---tga cctagtttgg tttagtgagc
7929-3 atcaaacaac agcaggaaat taatagaatt gactcttgct gagcatttca caataataca cctttaaaaa aaaa---tga cctagtttgg tttagtgagc
7929-4 atcaaacaac agcaggaaat taatagaatt gactcttgct gagcatttca caataataca cctttaaaaa aaaa---tga cctagtttgg tttagtga-c
7929-1 atcaaacaac agcaggaaat taatagaatt gactcttgct gagcatttca caataataca ccttaaaaaa aaaaaaacga cctagtttgg tttagtgagc
7929-5 atcaaacaac agcaggaaat taatagaatt gactcttgct gagcatttca caataataca cctttaaaaa aaaa---tga cctagtttgg tttagtga-c

                                                                                                                1
       9999999999 9999999999 9999999999 9999999999 9999999999 9999999999 9999999999 9999999999 9999999999 9999999990
       0000000001 1111111112 2222222223 3333333334 4444444445 5555555556 6666666667 7777777778 8888888889 9999999990
       1234567890 1234567890 1234567890 1234567890 1234567890 1234567890 1234567890 1234567890 1234567890 1234567890
7929-2 cggtcaggac tgtttattgg ttaatgtttt aatcttggtc gctagaggtc agtgcaaacc atccgagtcg tggcttt-tt tgctctagat attccacaca
7929-6 cggtcaggac tgtttattgg ttaatgtttt aatcttggtc gctagaggtc agtgcaaacc atccgagtcg tggctttatt tgctctagat attccacaca
7929-3 cggtcaggac tgtttattgg ttaatgtttt aatcttggtc gctagaggtc agtgcaaacc atccgagtcg tggcttt-tt tgctctagat attccacaca
7929-4 cggtcaggac tgtttattgg ttaatgtttt aatcttggtc gctatagatc agtgcaaacc atccgagtcg tggcttt-tt tgctctagat attccacaca
7929-1 cggtcaggac tgtttattgg ttaatgtttt aatcttggtc gctagaggtc agtgcaaacc atccgagtcg tggcttt-tt tgctctagat attccacaca
7929-5 cggtcaggac tgtttattgg ttaatgtttt aatcttggtc gctatagatc agtgcaaacc atccgagtcg tggcttt-tt tgctctagat attccacaca

       1111111111 1111111111 1111111111 1111111111 1111111111 1111111111 1111111111 1111111111 1111111111 1111111111
       0000000000 0000000000 0000000000 0000000000 0000000000 0000000000 0000000000 0000000000 0000000000 0000000001
       0000000001 1111111112 2222222223 3333333334 4444444445 5555555556 6666666667 7777777778 8888888889 9999999990
       1234567890 1234567890 1234567890 1234567890 1234567890 1234567890 1234567890 1234567890 1234567890 1234567890
7929-2 tgatgagcat ttacttttga ggcacaatat cttacataag tcttctgttg agtgtcattt aggctttgtg aaggtttaac agttaaactg ttaaattaga
7929-6 tgatgagcat ttacttttga ggcacaatat cttacataag tcttctgttg agtgtaattt aggctttgtg aaggtttaac agttaaactg ttaaatcaga
7929-3 tgatgagcat ttacttttga ggcacaatat cttacataag tcttctgttg agtgtcattt aggctttgtg aaggtttaac agttaaactg ttaaatcaga
```

```
7929-4 tgatgagcat ttacttttga ggcacaatat cttacataag tcttctgttg agtgtcattt aggctttgtg aaggtttaac agttaaactg ttaaatcaga
7929-1 tgatgagcat ttacttttga ggcacaatat cttacataag tcttctgttg agtgtccttt aggctttgtg aaggtttaac agttaaactg ttaaatcaga
7929-5 tgatgagcat ttacttttga ggcacaatat cttacataag tcttctgttg agtgtcattt aggctttgtg aaggtttaac agttaaactg ttaaatcaga

       1111111111 1111111111 1111111111 1111111111 1111111111 1111111111 1111111111 1111111111 1111111111 1111111111
       1111111111 1111111111 1111111111 1111111111 1111111111 1111111111 1111111111 1111111111 1111111111 1111111112
       0000000001 1111111112 2222222223 3333333334 4444444445 5555555556 6666666667 7777777778 8888888889 9999999990
       1234567890 1234567890 1234567890 1234567890 1234567890 1234567890 1234567890 1234567890 1234567890 1234567890
7929-2 tacagttcac agggttgaat gatatagatt ttctctggaa gaagaaatgg ccccatctag catgaagttt cattcctagg ctataaataa aagcacactt
7929-6 tatagttcac agggttgaat gatatagatt ttctctggaa gaagaaatgg ccccatctag catgaagttt cattcctagg ctataaataa aagcacactc
7929-3 tacagttcac agggttgaat gatatagatt ttctctggaa gaagaaatgg ccccatctag catgaagttt cattcctagg ctataaataa aagcacactc
7929-4 tacagttcac agcgttgaat gatatagatt ttctctggaa gaagaaatgg ccccatctag catgaagctt cattcctagg ctataaataa aagcacactc
7929-1 tacagttcac agggttgaat gatatagatt ttctctggaa gaagaaatgg ccccatctag catgaagttt cattcctagg ctataaataa aagcacactc
7929-5 tacagttcac agggttgaat gatatagatt ttctctggaa gaagaaatgg ccccatctag catgaagttt cattcctagg ctataaataa aagcacactc

       1111111111 1111111111 1111111111 1111111111 1111111111 1111111111 1111111111 1111111111 1111111111 1111111111
       2222222222 2222222222 2222222222 2222222222 2222222222 2222222222 2222222222 2222222222 2222222222 2222222223
       0000000001 1111111112 2222222223 3333333334 4444444445 5555555556 6666666667 7777777778 8888888889 9999999990
       1234567890 1234567890 1234567890 1234567890 1234567890 1234567890 1234567890 1234567890 1234567890 1234567890
7929-2 gcagaaaaag agtttctgac acaacagcac tacatttgaa atacaggtca aagttacaag gctgatgatt tcagacaaag gcaaacaagg aaagctaaaa
7929-6 gcagaaaaag agtttctgac acagcagcac tacatttgaa atacaggtca aagttacaag gctgatgatt tcagacaaag gcaaacaagg aaagctaaaa
7929-3 gcagaaaaag agtttctgac acagcagcac tacatttgaa atacaggtca aagttacaag gctgatgatt tcagacaaag ---------g aaagctaaaa
7929-4 gcagaaaaag agtttctgac acagcagcac tacatttgaa atacaggtca aagttacaag gctgatgatt tcagacaaag ---------g aaagctaaaa
7929-1 gcagaaaaag agtttctgac acagcagcac tacatttgaa atacaggtca aagttacaag gctgatgact tcagacaaag gcaaacaagg aaagctaaaa
7929-5 gcagaaaaag agtttctgac acagcagcac tacatttgaa atacaggtca aagttacaag gctgatgatt tcagacaaa- --------gg aaagctaaaa

       1111111111 1111111111 1111111111 1111111111 1111111111 1111111111 1111111111 1111111111 1111111111 1111111111
       3333333333 3333333333 3333333333 3333333333 3333333333 3333333333 3333333333 3333333333 3333333333 3333333334
       0000000001 1111111112 2222222223 3333333334 4444444445 5555555556 6666666667 7777777778 8888888889 9999999990
       1234567890 1234567890 1234567890 1234567890 1234567890 1234567890 1234567890 1234567890 1234567890 1234567890
```

```
7929-2 aatgtgtgat ccagctcaca gaacactaat attcctctca ctgacttctt gttttatttg cctttgtgtc gcagctcttt taccaggtga ggggcgacat
7929-6 aatgtgtgat ccagctcaca gaacactaat attcctctca ctgacttcct gtcttatttg cctttgtgtc gcagctcttt taccaggtga ggggcgacat
7929-3 aatgtgtgat ccagctcaca gaacactaat attcctctca ctgacttcct gttttatttg cctttgtgtc gcagctcttt taccaggtga ggggcgacat
7929-4 aatgtgtgat ccagctcaca gaacactaat attcctctca ctgacttcct gttttatttg cctttgtgtc gcagctcttt taccaggtga ggggcgacat
7929-1 aatgtgtgat gcagctcaca gaacactaat attcctctca ctgacttcct gttttatttg cctttgtgtc gcagctcttt taccaggtga ggggcgacat
7929-5 aatgtgtgat ccagctcaca gaacactaat attcctctca ctgacttcct gttttatttg cctttgtgtc gcagctcttt taccaggtga ggggcgacat

       1111111111 1111111111 1111111111 11111
       4444444444 4444444444 4444444444 44444
       0000000001 1111111112 2222222223 33333
       1234567890 1234567890 1234567890 12345
7929-2 ggttttgaaa gtgatagagg acggcaagca taaag
7929-6 ggttttgaaa gtgatagagg acggcaagca taaag
7929-3 ggttttgaaa gtgatagagg acggcaagca taaag
7929-4 ggttttgaaa gtgatagagg acggcaagca taaag
7929-1 ggttttgaaa gtgatagagg acggcaagca taaag
7929-5 ggttttgaaa gtgatagagg acggcaagca taaag
```

SNP LOCATIONS: 65T/G, 295T/G, 476G/C, 490C/T, 593C/A, 598T/A, 599T/A, 600C/T, 601A/G, 613A/T, 705A/C, 764T/G, 945G/T, 948G/A

15.

```
                1 1111111112 2222222223 3333333334 4444444445 5555555556 6666666667 7777777778 8888888889 9999999990
       1234567890 1234567890 1234567890 1234567890 1234567890 1234567890 1234567890 1234567890 1234567890 1234567890
7933-3 tgcctttt-gg tggaaataga cgggatgcag ctcgtgcagt caaaggcc-a tcttgaatta cattgctgga aaatacaatc tatatggaaa agaccttt-aa
7933-5 tgcctttt-gg tggaaataga cgggatgcag ctcgtgcagt caaaggccca tcttgaatta cattgctgga aaatacaatc tatatggaaa agacctttaa
7933-4 tgccttttgg tggaaataga cgggatgcag ctcgtgcagt caaaggcc-a tcttgaacta catcgctgga aaatacagtc tttacggaaa agaccttt-aa
7933-1 tgcctttt-gg tggaaataga cgggatgcag ctcgtgcagt caaaggcc-a tcttgaacta catcgctgga aaatacagtc tttacggaaa agatcttt-aa
7933-2 tgcctttt-gg tggaaataga cgggatgcag ctcgtgcagt caaaggcc-a tcttgaacta catcgctgga aaatacagtc tttacggaaa agaccttt-aa

       1111111111 1111111111 1111111111 1111111111 1111111111 1111111111 1111111111 1111111111 1111111111 1111111112
```

```
        0000000001 1111111112 2222222223 3333333334 4444444445 5555555556 6666666667 7777777778 8888888889 9999999990
        1234567890 1234567890 1234567890 1234567890 1234567890 1234567890 1234567890 1234567890 1234567890 1234567890
7933-3  agaacgggct atgtacgtgt caacttcgtt tatgaaaagt gttttgtaat tgatatgatt ccaagttgcc ttatcttcca ccacacccaa gcaattatca
7933-5  agaacgggct atgtacatgt caacttcgtt tatgaagagt gttttgtaat tgatatgatt ccaagttgcc ttatcttctt ccacacccaa gcaattatca
7933-4  agaacgggct atgtaaaagt caccttt--tt tattaacagt gttctg---- -------agt tcaagtg--- --ttttttca ccactgctaa gcaattctct
7933-1  agaacgggct atgtaaaagt caccttt--tt tatgaacagt gttctg---- -------agt ccaagtg--- --ttttttca ccactgctaa gcaattctct
7933-2  agaacgggct atgtaaaagt caccttt--tt tatgaacagt gttctg---- -------agt ccaagtg--- --ttttttca ccactgctaa gcaattctgt

        2222222222 2222222222 2222222222 2222222222 2222222222 2222222222 2222222222 2222222222 2222222222 2222222223
        0000000001 1111111112 2222222223 3333333334 4444444445 5555555556 6666666667 7777777778 8888888889 9999999990
        1234567890 1234567890 1234567890 1234567890 1234567890 1234567890 1234567890 1234567890 1234567890 1234567890
7933-3  ctgaatgcat gtgtttttgt cctgaaggat cgacatgtat tcagagggtg ccagtgatct aacggatttg attattatgt atcctttcaa accagctgaa
7933-5  ctgaatgcat gtgtttttgt cctgaaggat cgacatgtat tcagagggtg ccagcgatct aatggatttg attatgatgt atcctttcaa accagctgaa
7933-4  ctgaatgcat gtgtttttgt cctgcaggat cgacatgtat tcagagggag tcagtgatct catggatttg gttataatgt atcctttgac accacctgaa
7933-1  ctgaatgcat gt--ttttgt cctgcaggat cgacatgtat tcagagggag tcagtgatct catggatttg gttataatgt atcctttgac accacctgaa
7933-2  ctgaatgcat gtgtttttgt cctgcaggat cgacatgtat tcagagggag tcagtgatct catggatttg gttataatgt atcctttgac accacctgaa

        3333333333 3333333333 3333333333 3333333333 3333333333 3333333333 3333333333 3333333333 3333333333 3333333334
        0000000001 1111111112 2222222223 3333333334 4444444445 5555555556 6666666667 7777777778 8888888889 9999999990
        1234567890 1234567890 1234567890 1234567890 1234567890 1234567890 1234567890 1234567890 1234567890 1234567890
7933-3  cacaaacaga cacacctcag tactatagag caaaaggcca aagatcgctt tcttcctgtg tttgaaaagg tactgacaac tttttattct at-ttatccc
7933-5  cacaaacaga cacacctcag tactatagag caaaaggcca aagatcgctt tcttcctgtg tttgaaaagg tactgac--- tttttattct gt-ttatccc
7933-4  aacaaacaga aacacctgag taatatagag caaaaggcca aagatcgctt tcttcccgtg tttgaaaagg tacagacagt gaaacactat tcattctccc
7933-1  aacaaacaga aatacctcag taatatagag caaaaggcca aagatcgctt tcttcccgtg tttgaaaagg tacagacagt gaaacactat tcattctccc
7933-2  aacaaacaga aatacctcag taatatagag caaaaggcca aagatcgctt tcttcccgtg tttgaaaagg tacagacagt gaaacactat tcattctccc

        4444444444 4444444444 4444444444 4444444444 4444444444 4444444444 4444444444 4444444444 4444444444 4444444445
        0000000001 1111111112 2222222223 3333333334 4444444445 5555555556 6666666667 7777777778 8888888889 9999999990
        1234567890 1234567890 1234567890 1234567890 1234567890 1234567890 1234567890 1234567890 1234567890 1234567890
7933-3  actttgaagc ataattatcc acactggaga catgaaacta tggtttgttt ct-gtttttt agggtcttgc agactctcat ttcctggtgg gaaaccagct
```

```
7933-5 actttgaagc atgattatcc acactggaga catgaaacta tggtttgttt ct-gtttttt agggtcttgc agactctcat ttcctggtgg gaaaccagct
7933-4 agtttgaggc atgattatcc acgctggatg catgaaacta tggattgttt tttgtgtttt agggtctggg gaactctcag ttccttgtgg gaaaccagtt
7933-1 agtttgaggc atgattatcc acgctggatg catgaaacta tggattgttt tttgtgtttt agggtctggg gaactctcag ttccttgtgg gaaaccagtt
7933-2 agtttgaggc atgattatcc acgctggatg catgaaacta tggattgttt tttgtgtttt agggtctggg gaactctcag ttccttgtgg gaaaccagtt

       5555555555 5555555555 5555555555 5555555555 5555555555 5555555555 5555555555 5555555555 5555555555 5555555556
       0000000001 1111111112 2222222223 3333333334 4444444445 5555555556 6666666667 7777777778 8888888889 9999999990
       1234567890 1234567890 1234567890 1234567890 1234567890 1234567890 1234567890 1234567890 1234567890 1234567890
7933-3 gagccgagcc gacgttcacc ttcttgaagt cactctgatg ttgcaggagt tgctccctac catactttca acctttccca aaatccaggt gcttctgttt
7933-5 gagccgagcc gacgttcacc ttcttgaagt cactctgatg ttgcaggagt tgctccctac catactttca acctttccca aaatccaggt gcttctgttt
7933-4 gagccgtgct gacgttcacc ttcttgaagt cactctgatg ctgcaggagt tactccctac aatactttca acctttccca aaatccaggt gcttctgttt
7933-1 gagccgtgct gacgttcacc ttcttgaagt cactctgatg ctgcaggagt tactccctac aatactttca cactttccca aaatccaggt gcttca----
7933-2 gagccgtgct gacgttcacc ttcttgaagt cactctgatg ctgcaggagt tactccctac aatactctca cgctttccca aaatccaggt gcttca----

       6666666666 6666666666 6666666666 6666666666 6666666666 6666666666 6666666666 6666666666 6666666666 6666666667
       0000000001 1111111112 2222222223 3333333334 4444444445 5555555556 6666666667 7777777778 8888888889 9999999990
       1234567890 1234567890 1234567890 1234567890 1234567890 1234567890 1234567890 1234567890 1234567890 1234567890
7933-3 cagtttccca cacagacacc caaccccaaa agtac----- ------attt tttttaatat --tttgatgt tttagagtt- ---------- -------g--
7933-5 cagtttccca cacagacacc caaccccaaa aatac----- ------catt tcttcaatat gttttgatgt tttagagtt- ---------- -------g--
7933-4 cagtttccca cacagacacc caaccccaaa aatac----- ------attt ttttcaatat --tttgatgt tttagagtt- ---------- -------g--
7933-1 cagcttctca cacagacacc caaccccaga atcactaaac gaaaccatta ttttcaacat ---atgatgt tgtagagtaa gtttattaca ataacatcct
7933-2 cagtttctca cacagacacc caaccccaga atcactaaac gaaaccatta ttttcaacat ---atgatgt tgtagagtaa gtttattac- ---------t

       7777777777 7777777777 7777777777 7777777777 7777777777 7777777777 7777777777 7777777777 7777777777 7777777778
       0000000001 1111111112 2222222223 3333333334 4444444445 5555555556 6666666667 7777777778 8888888889 9999999990
       1234567890 1234567890 1234567890 1234567890 1234567890 1234567890 1234567890 1234567890 1234567890 1234567890
7933-3 ----cagt-- acattacaag aa-----cat tg-------a ---------- -----aaaac agccagaatt atgtcaagtt tca------- ----------
7933-5 ----cagt-- acattacaag aa-----cat tggcatatga ---------- -----aaaac agacagaatt acgtcaagtt tca------- ----------
7933-4 ----cagt-- acattacaag aa-----cat tg-------a ---------- -----aaaac agccagaatt atgtcaagtt tca------- ----------
7933-1 tgtgctatga atatgaaaaa aaacagctat agtttggtta gtgcatttga tgattaacat aattaaacct gtgctggttg tcagatatac agtgatagag
```

```
7933-2 tatgctatga atatgaaaaa aa-cagctat agtttagtta gtgcatttga tgattaacat aattaaacct gtgctggttg tcagatatac agtgatagag

       8888888888 8888888888 8888888888 8888888888 8888888888 8888888888 8888888888 8888888888 8888888888 8888888889
       0000000001 1111111112 2222222223 3333333334 4444444445 5555555556 6666666667 7777777778 8888888889 9999999990
       1234567890 1234567890 1234567890 1234567890 1234567890 1234567890 1234567890 1234567890 1234567890 1234567890
7933-3 ---------- -ctggccata taaa------ ---------- ----ttcaaa g--aacacta ctgtcatcta aaata----- ---gcggtac a---------
7933-5 ---------- -ctggccaaa taaa------ ---------- ----ttcaaa a--aacacta ctgtcatcta aaata----- ---gcggtac a---------
7933-4 ---------- -ctggccata taaaa----- ---------- --attcaaaa a--aacacta ctgtcatcta aaata----- ---gcggtac a---------
7933-1 agaagtgtgg tctggtttag tgaagcagtg agttcagccc acgattcaga gccataattc ctgtgatcca aaaaacaaat caagcggtac actttgcttt
7933-2 agaagtgtgg tctggtttag tgaagcagtg agttcagccc acgattcaga gccataattc ctgtgatcca aaaaacaaa- caagcggtac actttgcttt

                                                                                                                1
       9999999999 9999999999 9999999999 9999999999 9999999999 9999999999 9999999999 9999999999 9999999999 9999999990
       0000000001 1111111112 2222222223 3333333334 4444444445 5555555556 6666666667 7777777778 8888888889 9999999990
       1234567890 1234567890 1234567890 1234567890 1234567890 1234567890 1234567890 1234567890 1234567890 1234567890
7933-3 gcaaacactt tgcatgtatc tgtaatttat ctgatacaaa agactgccct tcttc-aagt actatggtta tgaattttgt ataacc-ttt catttgtgtt
7933-5 gcaaacactt tgcatgtatc tgtaatttat ctgatacaaa agactgccct tcttc-aagt actatggtta tgaattttgt ataacc-ttt catttgtgtt
7933-4 gcaaacactt tgcatgtatc tgtaatttat ctgatacaaa agactgccct tcttc-aagt actatggtta tgaattttgt ataacc-ttt catttgtttt
7933-1 gctgttttat tgcctgtatc tgttatttat ccattaaaga gaa-tgctct ttttccaagc attgtgattt tgaatcttgt ataacccttt catttgtttt
7933-2 gctgttgtat tacctgtatc tgt-atttat tcattaaaga gaagctcttt ttttccaagc attgtgattt tgaatcttgt ataacc-ttt catttgtttt

       1111111111 1111111111 1111111111 1111111111 111111111
       0000000000 0000000000 0000000000 0000000000 000000000
       0000000001 1111111112 2222222223 3333333334 444444444
       1234567890 1234567890 1234567890 1234567890 123456789
7933-3 -gcaggcgtt tcaagagaaa atgaaaggcc ttaccaacaa tcagcaagt
7933-5 -gcaggcgtt tcaagagaaa atgaa-ggcc ttaccaacaa tcagcaagt
7933-4 -gcaggcgtt tcaagagaaa atgaa-ggcc ttaccaacaa tcagcaagt
7933-1 -gcaggcgtt tcaagagaag atgaa-ggcc ttaccaacga tcagcaagt
7933-2 tgcaggcgtt tcaagagaag atgaaaggcc ttaccaacga tcag-----
```

SNP LOCATIONS: 58C/T, 64C/T, 78G/A, 82T/A, 85C/T, 116A/C, 118A/T, 123C/A, 144C/T, 159G/T, 185T/A, 186G/C, 188T/C, 197C/A, 200T/A, 225C/A, 249A/T, 251T/C, 261C/A, 271G/A, 288G/C, 290C/A, 295C/G, 301A/C, 311A/C, 313C/T, 323A/C, 357C/T, 374A/T, 381G/T, 382A/T, 383A/T, 384A/T, 385C/T, 387C/T, 389A/C, 392C/T, 396C/A, 402G/C, 408G/A, 423G/A, 429T/G, 430G/A, 444A/T, 451T/C, 456G/T, 468G/T, 470G/C, 471G/A, 472A/G, 480G/T, 486T/G, 500T/C, 508A/T, 511T/C, 541C/T, 552A/G, 561A/C, 571A/C, 596T/A, 608C/T, 962T/C, 964C/T, 966A/G, 969G/A, 972A/T, 978T/C, 998T/G, 1020A/G, 1039A/G

16.

```
                 1 1111111112 2222222223 3333333334 4444444445 5555555556 6666666667 7777777778 8888888889 9999999990
        1234567890 1234567890 1234567890 1234567890 1234567890 1234567890 1234567890 1234567890 1234567890 1234567890
7943-0  gcagcacacg ctttatcagt actaacaagt acatcttatt gaacataatt tgaatgcctt cggcagaatt caaatgagcc attttaatct agattaatct
7943-5  gcagcacacg ctttatcagt actaacaagt acatcttatt gaacataatt tgaatgcctt cggcagaatt caaatgagcc attttaatct agattaatct
7943-1  gcagcacacg ctttatcagt actaacaagt acatcttatt gaacataatt tgaatgcctt cggcagaatt caaatgagcc attttaatct agattaatct
7943-2  gcagcacacg ctttatcagt actaacaagt acatcttatt gaacataatt tgaatgcctt cggcagaatt caaatgagcc attttaatct agattaat--
7943-4  gcagcacacg ctttatcagt actaacaagt acatcttatt gaacataatt tgaatgcctt cggcagaatt caaatgagcc attttaatct agattaatct
7943-3  gcagcacacg ctttatcagt actaacaagt acatcttatt gaacataatt tgaatgcctt cggcagaact caaatgagcc attttaatct agattaatct

        1111111111 1111111111 1111111111 1111111111 1111111111 1111111111 1111111111 1111111111 1111111111 1111111112
        0000000001 1111111112 2222222223 3333333334 4444444445 5555555556 6666666667 7777777778 8888888889 9999999990
        1234567890 1234567890 1234567890 1234567890 1234567890 1234567890 1234567890 1234567890 1234567890 1234567890
7943-0  agattaattc caagattaca gtgagattaa tctagattaa aaaaaataat ctatgcccac cactaaaaaa aa--tacatt taaatttata ataataacca
7943-5  agattaattc caagattaca gtgagattaa tctagattaa aaaaattaat ctatgcccac cactaaaaaa aa--tacatt taaatttata ataataacca
7943-1  agattaattc caagattaca gtgagattaa tctagattaa aaaaattaat ctatgcccac cactaaaaaa aaaatacatt taaacttata ataataacca
7943-2  --------tc caagattaca gtgagattaa tctagattaa aaaaattaat ctatgcccac cactaaaaaa aa--tacatt taaatttata ataataacca
7943-4  agattaattc caagattaca gtgagattaa tctagattaa aaaaattaat ctatgcccac cactaaaaaa aa--tacatt taaatttata ataataacca
7943-3  agattaattc caagattaca gtgagattaa tctagattaa aaaaatgtat ctatgcctac cactaaaaaa aaaatacatt taaatttata ataataacca

        2222222222 2222222222 2222222222 2222222222 2222222222 2222222222 2222222222 2222222222 2222222222 2222222223
        0000000001 1111111112 2222222223 3333333334 4444444445 5555555556 6666666667 7777777778 8888888889 9999999990
        1234567890 1234567890 1234567890 1234567890 1234567890 1234567890 1234567890 1234567890 1234567890 1234567890
7943-0  taaaaatata gtaaaactat attacataat tgtaatctaa aattatatat atattagtgg tgggccgtta tcggcgttaa cgtgctgcgt taacgtgaga
7943-5  taaaaatata gtaaaactat attacataat tgtaatctga aattatatat atattagttg tgggccgtta tcggcgttaa cgtg------ -------aga
7943-1  taaaaatata gtaaaactat attacataat tgtaatctga aattatatat atattagtgg tgggccgcta tcagcgttaa cgtgctgcgt taacgtgaga
7943-2  taaaaatata gtaaaactat attacataat tgtaatctga aattatatat atattagtgg tgggccgtta tcggcgttaa cgtgctgcgt taacgtgaga
7943-4  taaaaatata gtaaaactat attacataat tgtaatctga aattatatat atattagtgg tgggccgtta tcggcgttaa cgtgctgcgt taacgtgaga
7943-3  taaaaatata gtaaaactat attacataat tgtaatctga aattatatat atattagtgg tgggccgtta tcggcgttaa cgtgctgcgt taacgtgaga
```

```
        3333333333 3333333333 3333333333 3333333333 3333333333 3333333333 3333333333 3333333333 3333333333 3333333334
        0000000001 1111111112 2222222223 3333333334 4444444445 5555555556 6666666667 7777777778 8888888889 9999999990
        1234567890 1234567890 1234567890 1234567890 1234567890 1234567890 1234567890 1234567890 1234567890 1234567890
7943-0  ctcttatcgg gcgataaaaa aaa-tatcac cgttaatcta ttctcaaagt tgggtt-ggg agctgggtct atactacgca agctatgatg actttcgccg
7943-5  ctcttatcgg gcgataaaaa aaa-tatcgc cgttaatcta ttctcaaagt tgggtt-ggg agctgggtct atactacgca agctatgatg actttcgccg
7943-1  ctcttatcgg gcgataaaaa aaaatatcgc cattaatcta ttctcaaagt tgggtt-ggg agctgggtct atactacgca agctatgatg actttcgccg
7943-2  ctcttatcgg gcgataaaaa aaa-tatcgc ccttaatcta ttctcaaagt tgggtt-ggg agctgggtct atactacgca agctatgatg actttcgccg
7943-4  ctcttatcgg gcgataaaaa aaa-tatcgc cattaatcta ttctcaaagt tgggtt-ggg agctgggtct atactacgca agctatgatg actttcgccg
7943-3  ctcttatcgg gcgataaaaa aaaatatcgc cattaatcta ttctcaaagt tgggtt-ggg agctgggtct atactacgca agctacgatg actttcgccg

        444444
        000000
        123456
7943-0  tgatag
7943-5  tgatag
7943-1  tgatag
7943-2  tgatag
7943-4  tgatag
7943-3  tgatag
```

SNP LOCATIONS: 332A/G

17.

```
                 1 1111111112 2222222223 3333333334 4444444445 5555555556 6666666667 7777777778 8888888889 9999999990
        1234567890 1234567890 1234567890 1234567890 1234567890 1234567890 1234567890 1234567890 1234567890 1234567890
7944-2  tggtgacact ggctctgggt tcacctctct cctaatggaa cggctctctg tcgactacgg gaagaagtca aaacttgagt ttgctgtcta tccagctcct
7944-3  tggtgacact ggctctgggt tcacctctct cctaatggaa cggctctctg tcgactatgg gaagaagtca aaacttgagt tcgctgtcta tccagctcct
7944-1  tggtgacact ggctctgggt tcacctctct cctaatggaa cggctctctg tcgactacgg gaagaagtca aaacttgagt ttgctgtcta tccagctcct
7944-0  tggtgacact ggctctgggt tcacctctct cctaatggaa cggctctctg tcgactacgg gaagaagtca aaacttgagt tcgctgtcta tccagctcct
7944-4  tggtgacact ggctctgggt tcacctctct cctaatggaa cggctctctg tcgactacgg gaagaagtca aaattggaat ttgccattta tccagctcct
```

```
        1111111111 1111111111 1111111111 1111111111 1111111111 1111111111 1111111111 1111111111 1111111111 1111111112
        0000000001 1111111112 2222222223 3333333334 4444444445 5555555556 6666666667 7777777778 8888888889 9999999990
        1234567890 1234567890 1234567890 1234567890 1234567890 1234567890 1234567890 1234567890 1234567890 1234567890
7944-2  caagtatcca ctgcagtggt ggagccctac aactccatcc ttaccaccca caccaccctc gagcactccg actgtgcctt catggtggac aacgaggcca
7944-3  caagtatcca ctgcagtggt ggagccctac aactccatcc ttaccaccca caccaccctc gagcactccg actgtgcctt catggtggac aacgaggcca
7944-1  caagtgtcca ctgcagtggt ggagccctac aactccatcc ttaccaccca caccaccctc gagcactccg actgtgcctt catggtggac aacgaggcca
7944-0  caagtgtcca ctgcagtggt ggagccctac aactccattc ttaccaccca caccaccctc gagcactccg actgtgcctt catggtagac aatgaggcca
7944-4  caagtgtcca ctgcagtggt ggagccctac aactccatcc ttaccaccca caccaccctc gagcactccg actgtgcctt catggtggac aatgaggcca

        2222222222 2222222222 2222222222 2222222222 2222222222 2222222222 2222222222 2222222222 2222222222 22222222
        0000000001 1111111112 2222222223 3333333334 4444444445 5555555556 6666666667 7777777778 8888888889 99999999
        1234567890 1234567890 1234567890 1234567890 1234567890 1234567890 1234567890 1234567890 1234567890 12345678
7944-2  tctacgatat ctgccgtaga aacctcgaca tcgagcgccc cacctacaca aacctcaaca ggttgattgg gcagatcgtt tcctccatca cagcatcc
7944-3  tctacgatat ctgccgtaga aacctcgaca tcgagcgccc cacctacaca aacctcaaca ggttgattgg gcagatcgtt tcctccatca cagcatcc
7944-1  tctacgatat ctgccgtaga aacctcgaca tcgagcgccc cacctacaca aacctcaaca ggttgattgg gcagatcgtt tcctccatca cagcatcc
7944-0  tctacgatat ctgccgtaga aacctcgaca tcgagcgtcc cacctacaca aaccccaaca ggctgatcgg gcagattgtt tcctccatca cagcatcc
7944-4  tctacgatat ctgccgtaga aacctcgaca tcgagcgccc cacctacaca aacctcagca ggctgatcgg gcagatcgtt tcctccatca cagcatcc
```

SNP LOCATIONS: 82T/C, 106G/A, 193C/T, 263T/C, 268T/C

18.

```
                 1 1111111112 2222222223 3333333334 4444444445 5555555556 6666666667 7777777778 8888888889 9999999990
        1234567890 1234567890 1234567890 1234567890 1234567890 1234567890 1234567890 1234567890 1234567890 1234567890
7947-2  gggtggagaa atacaagaac atgatcacta aagttggcaa agcaaagaag atggatccgg ctgtgattgc tgctatgata tccagagagt ccagagctgg
7947-3  gggtggagaa atacaagaac atgatcacta aagttggcaa agcaaagaag atggatccgg ctgtgattgc tgctatgata tccagagagt ccagagctgg
7947-6  gggtggagaa atacaagaac atgatcacta aagttggcaa agcaaagaag atggatccgg ctgtgattgc tgctatgata tccagagagt ccagagctgg
7947-7  gggtggagaa atacaagaac atgatcacta aagttggcaa agcaaagaag atggatccgg ctgtgattgc tgctatgata tccagagagt ccagagctgg
7947-4  gggtggagaa atacaagaac atgatcacta aagttggcaa agcaaagaag atggatccgg ctgtgattgc tgctatgata tccagagagt ccagagctgg
7947-5  gggtggagaa atacaagaac atgatcacta aagttggcaa agcaaagaag atggatctgg ctgtgattgc tgctatgata tccagagagt ccagagctgg
```

```
7947-1 gggtggagaa atacaagaac atgatcacta aagttggcaa agcaaaaaag atggatccgg ctgtgattgc tgctatgata tccagagagt ccagagctgg
7947-0 gggtggagaa atacaagaac atgatcacta aagttggcaa agcaaaaaag atggatccgg ctgtgattgc tgctatgata tccagagagt ccagagctgg

       1111111111 1111111111 1111111111 1111111111 1111111111 1111111111 1111111111 1111111111 1111111111 1111111112
       0000000001 1111111112 2222222223 3333333334 4444444445 5555555556 6666666667 7777777778 8888888889 9999999990
       1234567890 1234567890 1234567890 1234567890 1234567890 1234567890 1234567890 1234567890 1234567890 1234567890
7947-2 agccgtcctg aagaatggat gggaacccgc aggcaatggc tttggcctta tgcaggttca ttacaactat gaatctacgc tatttaaaat tgaattagac
7947-3 agccgtcctg aagaatggat gggaacccgc aggcaatggc tttggcctta tgcaggttca ttacaactat gaatctactc tatttaaaat tgaattagac
7947-6 aaccgtcctg aagaacggat gggaacccgc aggcaatggc tttggcctta tgcaggttca ttacaactat gaatctactc tatttaaaat ttaattagac
7947-7 aaccgtcctg aagaacggat gggaacccgc aggcaatggc tttggcctta tgcaggttca ttacaactat gaatctactc tatttaaaat ttaattagac
7947-4 agccgtcctg aagaatggat gggaacccgc aggcaatggt tttggcctta tgcaggttca ttacaactat gaatctactc tatttaaaat ttaattagac
7947-5 agccgtcctg aagaatggat gggaacccgc aggcaatggc tttggcctta tgcaggttca ttacaactat gaatctactc tatttaaaat ttaattagac
7947-1 agccgtcctg aagaatggat gggaacccgc aggcaatggc tttggcctta tgcaggttca ttacaactat gaatctactc tatttaaaat ttaattagac
7947-0 agccgtcctg aagaatggat gggaacccgc aggcaatggc tttggcctta tgcag----- ---------- ---------- ---------- ----------
       2222222222 2222222222 2222222222 2222222222 2222222222 2222222222 2222222222 2222222222 2222222222 2222222223
       0000000001 1111111112 2222222223 3333333334 4444444445 5555555556 6666666667 7777777778 8888888889 9999999990
       1234567890 1234567890 1234567890 1234567890 1234567890 1234567890 1234567890 1234567890 1234567890 1234567890
7947-2 ctaagatga ggcttataca tg-tatgagt gtgcacatta aataaaattc aaggtgttta atttgtcaca ataatcatcc atgcaaagtt ttactaaata
7947-3 ctaagatga ggcttataca tg-tatgagt gtgcacatta aataaaattc aaggtgttta atttgtcaca ataatcatcc atgcaaagtt ttactaaata
7947-6 ctaagatga ggcttataca tg-tatgagt gtgcacatta aatacaattc aaggtgttta atttgtcaca ataatcatcc atgcaaagta ttactaaata
7947-7 ctaagatga ggcttataca tg-tatgagt gtgcacatta aatacaattc aaggtgttta atttgtcaca ataatcatcc atgcaaagta ttactaaata
7947-4 ctaagatga ggcttataca tg-tatgagt gtgcacatta aataaaattc aaggtgttta atttgtcaca ataatcatcc atgcaaagtg ttactaaata
7947-5 ctaagatga ggcttataca tg-tatgagt gtgcacatta aataaaattc aaggtgttta atttgtcaca ataatcatcc atgcaaagtg ttactaaata
7947-1 ctaagatga ggcttataca tg-tatgagt gtgcacatta aataaaattc aaggtgttta atttgtcaca ataatcatcc atgcaaagtg ttactaaata
7947-0 --------- ---------- ---------- ---------- ---------- ---------- ---------- ---------- ---------- ----------

       3333333333 3333333333 3333333333 3333333333 3333333333 3333333333 3333333333 3333333333 3333333333 3333333334
       0000000001 1111111112 2222222223 3333333334 4444444445 5555555556 6666666667 7777777778 8888888889 9999999990
       1234567890 1234567890 1234567890 1234567890 1234567890 1234567890 1234567890 1234567890 1234567890 1234567890
7947-2 tttctgctgt ttcaaagctt gatgattttt tttt-ctcca ggttgacaaa cgctaccaca ctccggttgg tgcatgggac agtgagcagc atgtcacaca
```

```
7947-3 tttctgctgt ttcaaagctt gatgattttt tttt-ctcca ggttgacaaa cgctaccaca ctccggttgg tgcatgggac agtgagcagc atgtcacaca
7947-6 tttctgctgt ttcaaagctt gatgattttt tttt-ctcca ggttgacaaa cgctaccaca ctccggttgg tgcatgggac agtgagcagc atgtcacaca
7947-7 tttctgctgt ttcaaagctt gatgattttt tttt-ctcca ggttgacaaa cgctaccaca ctccggttgg tgcatgggac agtgagcagc atgtcacaca
7947-4 tttctgctgt ttcaaagctt gatgattttt tttt-ctcca ggttgacaaa cgctaccaca ctccggttgg tgcatgggac agtgagcagc atgtcacaca
7947-5 tttctgctgt ttcaaagctt gatgattttt tttt-ctcca ggttgacaaa cgctaccaca ctccggttgg tgcatgggac agtgagcagc atgtcacaca
7947-1 tttctgctgt ttcaaagctt gatgattttt tttttctcca ggttgacaaa cgctaccaca ctccggttgg tgcatgg-ac agtgagcagc atgtcacaca
7947-0 ---------- ---------- ---------- ---------- -gttgacaaa cgctaccaca ctccgggtgg tgcatgggac agtgagcagc atgtcacaca

       4444444444 4444444444 44
       0000000001 1111111112 22
       1234567890 1234567890 12
7947-2 agctacagag atactcattg gc
7947-3 agctacagag atactcattg gc
7947-6 agctacagag atactcattg gc
7947-7 agctacagag atactcattg gc
7947-4 agctacagag atactcattg gc
7947-5 agctacagag atactcattg gc
7947-1 agctacagag atactcattg gc
7947-0 agctacagag atactcattg gc
```

SNP LOCATIONS: 47G/A, 102G/A, 116T/C, 192T/G, 244A/C, 290G/A/T

19.

```
                1 1111111112 2222222223 3333333334 4444444445 5555555556 6666666667 7777777778 8888888889 9999999990
       1234567890 1234567890 1234567890 1234567890 1234567890 1234567890 1234567890 1234567890 1234567890 1234567890
7953-3 tgccagagta tcagtccaag aacatcttct tctctccatt cagtgtgtcc atggcccttt ctgagctgtg tttaggagcc ggtggtgaaa ccaaagagca
7953-5 tgccagagta tcagtccaag aacatcttct tctctccatt cagtgtgtcc atggcccttt ctgagctgtg tttaggagcc ggtggtgaaa ccaaagagca
7953-2 tgccagagta tcagtccaag aacatcttct tctctccatt cagtgtgtcc atggcccttt ctgagctgtg tttaggagcc ggtggtgaaa ccaaagagca
7953-7 tgccagagta tcagtccaag aacatcttct tctctccatt cagtgtgtcc atggcccttt ctgagctgtg tttaggagcc ggtggtgaaa ccaaagagca
7953-1 tgccagagta tcagtccaag aacatcttct tctctccctt cagtgtgtcc atggcccttt ctgagctgtc tttaggagcc ggtggtgaaa ccaaagagca
```

```
7953-6 tgccagagta tcagtccaag aacatcttat tctctccctt cagtgtgtcc atggcccttt ctgagctgtc tttaggagcc ggtggtgaaa ccaaagagca
7953-0 tgccagagta tcagtccaag aacatcttct tctctccctt cagtgtgtcc atggcccttt ctgagctgtc tttaggagcc ggtggtgaaa ccaaagagca
7953-4 tgccagagta tcagtccaag aacatcttct tctctccatt cagtgtgtcc atggcccttt ctgagctgtc tttaggagcc ggtggtgaaa ccgaagagca

       1111111111 1111111111 1111111111 1111111111 1111111111 1111111111 1111111111 1111111111 1111111111 1111111112
       0000000001 1111111112 2222222223 3333333334 4444444445 5555555556 6666666667 7777777778 8888888889 9999999990
       1234567890 1234567890 1234567890 1234567890 1234567890 1234567890 1234567890 1234567890 1234567890 1234567890
7953-3 gcttctcagt ggcatcggcc ataacagctc ggtcttcagc actgaagaaa tgcaccagat gttccacagt ctcctggaag aaatcgacca gaggacaggg
7953-5 gcttctcagt ggcatcggcc ataacagctc ggtcttcagc actgaagaaa tgcaccagat gttccacagt ctcctggaag aaatcgacca gaggacaggg
7953-2 gcttctcagt ggcatcggcc ataacagctc ggtcttcagc actgtagaaa tgcaccagat gttccacagt ctcctggaag aaatcgacca gaggacaggg
7953-7 gcttctcact ggcatcggcc ataacagctc ggtcttcagc actgaagaaa tgcaccagat gttccacagt ctcctggaag aaatcgacca gaggacaggg
7953-1 gcttctcagt ggcatcggcc ataacagctc ggtcttcagc actgaagaaa tgcaccagat gttccacagt ctcctggaag aaatcgacca gaggacaggg
7953-6 gcttctcagt ggcatcggcc ataacagctc ggtcttcagc actgaagaaa tgcaccagat gttccacagt ctcctggaag aaatcgacca gaggacaggg
7953-0 gcttctcagt ggcatcggcc ataacagctc ggtcttcagc actgaagaaa tgcaccagat gttccacagt ctcctggaag aaatcgacca gaggacaggg
7953-4 gcttctcact ggcatcggcc ataacagctc ggtcttcagc actgaagaaa tgcaccagat gttccacagt ctcctggaag aaatcgacca gaggacaggg

       2222222222 2222222222 2222222222 2222222222 2222222222 2222222222 2222222222 2222222222 2222222222 2222222223
       0000000001 1111111112 2222222223 3333333334 4444444445 5555555556 6666666667 7777777778 8888888889 9999999990
       1234567890 1234567890 1234567890 1234567890 1234567890 1234567890 1234567890 1234567890 1234567890 1234567890
7953-3 gtggacattg atgtcggcag tgctttatat gcaagcgaca aattaaaggt ccttcctgag ttcttaaagg agataaagga gttttaccac tctgatggct
7953-5 gtggacattg atgtcggcag tgctttatat gcaagcgaca aattaaaggt ccttcctgag ttcttaaagg agataaagga gttttaccac tctgatggct
7953-2 gtggacattg atgtcggcag tgctttatat gcaagcgaca aattaaaggt ccttcctgag ttcttaaagg agataaagga gttttaccac tctgatggct
7953-7 gtggacattg acgtcggcag tgctttatat gcaagcgaca aattaaaggt ccttcctgag ttcttaaaag agatgaagga gttttaccac tctgatggct
7953-1 gtggacatta atgtcggcag tgctctatat gcaagcaaca aattaaagct ccttcctgag ttcttaaagg agataaagga gttttaccac tctgacggct
7953-6 gtggacatta atgtcggcag tgctctatat gcaagcaaca aattaaagct ccttcctgag ttcttaaagg agataaagga gttttaccac tctgacggct
7953-0 gtggacatta atgtcggcag tgctctatat gcaagtgaca aattaaagct ccttcctgag ttcttaaagg agataaagga gttttaccac tctgacggct
7953-4 gtggacatta atgtcggcag tgctctatat gcaagcaaca aattaaagct ccttcctgag ttcttaaagg agataaagga gttttaccac tctgacggct

       3333333333 3333333333 3333333333 3333333333 3333333
       0000000001 1111111112 2222222223 3333333334 4444444
```

```
       1234567890 1234567890 1234567890 1234567890 1234567
7953-3 tcactgtgga tttcagcgtc aaagaaacac tggataaaat caacacg
7953-5 tcactgtgga tttcagcgtc aaagaaacac tggataaaat caacacg
7953-2 tcactgtgga tttcagcgtc aaagaaacac tggataaaat caacacg
7953-7 tcactgtgga tttcagcgtc aaagaaacac tggataaaat caacacg
7953-1 tcactgtgga tttcagcgtc aaagaaacac tggataaaat caacacg
7953-6 tcactgtgga tttcagcgtc aaagaaacac tggataaaat caacacg
7953-0 tcactgtgga tttcagcgtc aaagaaacac tggataaaat caacacg
7953-4 tcactgtgga tttcagcgtc aaagaaacac tggataaaat caacacg
```

SNP LOCATIONS: 38A/C, 70G/C, 109G/C, 210G/A, 225C/T, 237G/A, 249G/C, 296T/C

20.

```
                1 1111111112 2222222223 3333333334 4444444445 5555555556 6666666667 7777777778 8888888889 9999999990
       1234567890 1234567890 1234567890 1234567890 1234567890 1234567890 1234567890 1234567890 1234567890 1234567890
7955-2 taaaagaaca caacgggcac atcaccggta aaaactcctc gtaaactgaa tttcaaacat gaatgttaca tttcttaaat tcctcacatt atgttgaaat
7955-6 taaaagaaca caacgggcac atcaccggta aaaactcctc gtaaactgaa tttcaaacat gaatgttaca tttcttaaat tcctcacatt atgttgaaat
7955-3 taaaagaaca caacgggcac atcaccggta aaaactcctc gtaaactgaa tttcaaacat gaatgttaca tttcttaaat tcctcacatt atgttgaaat
7955-5 taaaagaaca caacgggcac atcaccggta aaaactcctc gtaaactgaa tttcaaacat gaatgttaca tttcttaaat tcctcacatt atgttgaaat
7955-4 taaaagaaca caacgggcac atcaccggta aaaactcctc gtaaactgaa tttcaaacac gaatgttaca tttcttaaat tcctcacatt atgttgaaat
7955-7 taaaagaaca caacgggcac atcaccggta aaaactcctc gtaaactgaa tttcaaacat gaatgttaca tttcttaaat tcctcacatt atgttgaaat
7955-1 taaaagaaca caacgggcac atcaccggta aaaactcctc gtaaactgaa tttcaaacat gagtgttaca tttcttaaat tcctcacatt atgttgaaat
```

```
       1111111111 1111111111 1111111111 1111111111 1111111111 1111111111 1111111111 1111111111 1111111111 1111111112
       0000000001 1111111112 2222222223 3333333334 4444444445 5555555556 6666666667 7777777778 8888888889 9999999990
       1234567890 1234567890 1234567890 1234567890 1234567890 1234567890 1234567890 1234567890 1234567890 1234567890
7955-2 atacagttga tcgttatttg tttgttcttg ttgttgtttt ccaggcattg attgggctcc taaaagtgat cgtattgtga cctgtggagc tgaccgtaat
7955-6 atacagttga tcgttatttg tttgttcctg ttgttgtttt ccaggcattg attgggctcc taaaagtgat cgtattgtga cctgtggagc tgaccgtaat
7955-3 atacagttga tcgttatttg tttgttcttg ttgttgtttt ccaggcattg attgggctcc taaaagtgat cgtattgtga cctgtggagc tgaccgtaat
7955-5 atacagttga tcgttatttg tttgttcttg ttgttgtttt ccaggcattg attgggctcc taaaagtgat cgtattgtga cctgtggagc tgaccgtaat
```

```
7955-4 atacagttga tcgttatttg tttgttcttg ttgttgtttt ccaggcattg attgggctcc taaaagtgat cgtattgtga cctgtggagc tgaccgtaat
7955-7 atacagttga tcgttatttg tttgttcttg ttgttgttct ccaggcattg attgggctcc taaaagtgat cgtattgtga cctgtggagc tgaccgtaat
7955-1 atacagttga tcattatttg tttgttcttg ttgttgtttt ccaggcattg attgggctcc taaaagtgat cgtattgtga cctgtggagc tgaccgtaat

       2222222222 2222222222 2222222222 2222222222 2222222222 2222222222 2222222222 2222222222 2222222222 2222222223
       0000000001 1111111112 2222222223 3333333334 4444444445 5555555556 6666666667 7777777778 8888888889 9999999990
       1234567890 1234567890 1234567890 1234567890 1234567890 1234567890 1234567890 1234567890 1234567890 1234567890
7955-2 gcttatgtat ggagtcaaaa ggacggcgtg tggaaaccca cccttgttat tctcaggatc aaccgtgctg ccacgtttgt gaaatggtct ccgctggaga
7955-6 gcttatgtat ggagtcaaaa ggacggcgtg tggaaaccca cccttgttat tctcaggatc aaccgtgctg ccacgtttgt gaaatggtct ccgctg-aga
7955-3 gcttatgtat ggagtcaaaa ggacggcgtg tggaaaccca cccttgttat tctcaggatc aaccgtgctg ccacgtttgt gaaatggtct ccgctggaga
7955-5 gcttatgtat ggagtcaaaa ggacggcgtg tggaaaccca cccttgttat tctcaggatc aaccgtgctg ccacgtttgt gaaatggtct ccgctggaga
7955-4 gcttatgtat ggagtcaaaa ggacggcgtg tggaaaccca cccttgttat tctcaggatc aaccgtgctg ccacgtttgt gaaatggtct ccgctggaga
7955-7 gcttatgtat ggagtcaaaa ggacggcgtg tggaaaccca cccttgttat tctcaggatc aaccgtgctg ccacgtttgt gaaatggtct ccgctggaga
7955-1 gcttatgtat ggagtcaaaa ggacggcgtg tggaaaccca cccttgttat tctcaggatc aaccgtgctg ccacgtttgt gaaatggtct ccgctggaga

       3333333333 3333333333 3333333333 3333333333 3333333333 3333333
       0000000001 1111111112 2222222223 3333333334 4444444445 5555555
       1234567890 1234567890 1234567890 1234567890 1234567890 1234567
7955-2 acaagtttgc agtggggagt ggagcgcgac tcatatccgt ttgctacttt gagtctg
7955-6 acaagtttgc agtggggagt ggagcgcgac tcatatccgt ttgctacttt gagtctg
7955-3 acaagtttgc agtggggagt ggagcgcgac tcatatccgt ttgctacttt gagtctg
7955-5 acaagtttgc agtggggagt ggagcgcgac tcatatccgt ttgctacttt gagtctg
7955-4 acaagtttgc agtggggagt ggagcgcgac tcatatccgt ttgctacttt gagtctg
7955-7 acaagtttgc agtggggagt ggagcgcgac tcatatccgt ttgctacttt gagtctg
7955-1 acaagtttgc agtggggagt ggagcgcgac tcatatccgt ttgctacttt gagtctg
```

SNP LOCATIONS: NULL

21.

```
                1 1111111112 2222222223 3333333334 4444444445 5555555556 6666666667 7777777778 8888888889 9999999990
```

	1234567890	1234567890	1234567890	1234567890	1234567890	1234567890	1234567890	1234567890	1234567890	1234567890
7957-4	cttccacgag	accctttacc	agaaagctga	tgatggcaca	ccctttgcca	aaatgatcaa	ggacaggggc	attgttgtgg	gaatcaaggt	acaagattta
7957-7	cttccacgag	accctttacc	agaaagctga	tgatggcaca	ccctttgcca	aaatgatcaa	ggacaggggc	attgttgtgg	gaatcaaggt	acaagattta
7957-2	cttccacgag	accctttacc	agaaagctga	tgatggcaca	ccctttgcca	aaatgatcaa	ggacaggggc	attgttgtgg	gaatcaaggt	acaagattta
7957-3	cttccacgag	accctttacc	agaaagctga	tgatggcaca	ccctttgcca	aaatgatcaa	ggacaggggc	attgttgtgg	gaatcaaggt	acaagatttc
7957-5	cttccacaag	accctttacc	agaaagctga	tgatggcaca	ccctttgcca	aaatgatcaa	ggacaggggc	attgttgtgg	gaatcaaggt	acaagatttc
7957-6	cttccacgag	accctttacc	agaaagctga	tgatggcaca	ccctttgcca	aaatgatcaa	ggacaggggc	attgttgtgg	gaatcaaggt	acaagattta
7957-1	----------	----------	----------	----------	----------	----------	----------	----------	----------	----------

	1111111111	1111111111	1111111111	1111111111	1111111111	1111111111	1111111111	1111111111	1111111111	1111111112
	0000000001	1111111112	2222222223	3333333334	4444444445	5555555556	6666666667	7777777778	8888888889	9999999990
	1234567890	1234567890	1234567890	1234567890	1234567890	1234567890	1234567890	1234567890	1234567890	1234567890
7957-4	atttaaatta	ataattttat	tcaactagga	tgcattacac	tgatcaaaag	ttacagtagt	catttaaaat	gtttctaaag	atttctattt	cagataaatc
7957-7	atttaaatta	ataattttat	tcaactagga	tgcattacac	tgatcaaaag	ttacagtagt	catttaaaat	gtttctaaag	atttctattt	cagataaatc
7957-2	atttaaatta	ataattttat	tcaactagga	tgcattacac	tgatcaaaag	ttacagtagt	catttaaaat	gtttctaaag	atttctattt	cagataaatc
7957-3	atttaaatta	acaatttcat	tcaactagga	tgcattacac	tgatcaaaag	ttacagtagc	catttaaaat	gtttctaaag	atttctattt	cagataaatc
7957-5	atttaaatta	ataattttat	tcaactagga	tgcattacac	tgatcaaaag	ttacagtagt	catttaaaat	gtttctaaag	atctctattt	cagataaatc
7957-6	atttaaatta	ataattttat	tcaactagga	tgcattacac	tgatcaaaag	ttacagtagt	catttaaaat	gtttctaaag	atttctattt	cagataaatc
7957-1	----------	----------	----------	----------	----------	----------	----------	----------	----------	----------

	2222222222	2222222222	2222222222	2222222222	2222222222	2222222222	2222222222	2222222222	2222222222	2222222223
	0000000001	1111111112	2222222223	3333333334	4444444445	5555555556	6666666667	7777777778	8888888889	9999999990
	1234567890	1234567890	1234567890	1234567890	1234567890	1234567890	1234567890	1234567890	1234567890	1234567890
7957-4	ctgaagagat	catggtttcc	acaaaattat	taagcagcac	aattgttttt	aacgctgata	ataataataa	atgtttcttg	agcattaaat	cagcatatta
7957-7	ctgaagagat	catggtttcc	acaaaattat	taagcagcac	aattgttttt	aacgctgata	ataataataa	atgtttcttg	agcattaaat	cagcatatta
7957-2	ctgaagagat	catggtttcc	acaaaattat	taagcagcac	aattgttttt	aacgctgata	ataataataa	atgtttcttg	agcattaaat	cagcatatta
7957-3	ctgaagagat	catggtttcc	acaaaattat	taagcagcac	aattgttttt	aacgctgata	ataataataa	atgtttattg	agcattaaat	cagcatatta
7957-5	ctgaagagat	catggtttcc	acaaaattat	taagcagcac	aattgttttt	aacgctgata	ataataataa	atgtttcttg	agcattaaat	cagcatatta
7957-6	ctgaagagat	catggtttcc	acaaaattat	taagcagcac	aattgttttt	aacgctgata	ataataataa	atgtttcttg	agcattaaat	cagcatatta
7957-1	----------	----------	----------	----------	----------	----------	----------	----------	----------	----------

```
        3333333333 3333333333 3333333333 3333333333 3333333333 3333333333 3333333333 3333333333 3333333333 3333333334
        0000000001 1111111112 2222222223 3333333334 4444444445 5555555556 6666666667 7777777778 8888888889 9999999990
        1234567890 1234567890 1234567890 1234567890 1234567890 1234567890 1234567890 1234567890 1234567890 1234567890
7957-4 aatttctgaa ggatcatgtg acacttaaga ctggagtaat gatgctgaaa attcagcttt acattacaag aataaactac attttaataa agtttttatt
7957-7 aattcctgaa ggatcatgtg acacttaaga ctggagtaat gatgctgaaa attcagcttt acattacaag aataaactac attttaataa agtttttatt
7957-2 aatttctgaa ggatcatgtg acacttaaga ctggagtaat gatgctgaaa attctgcttt acattacaag aataaactac attttaataa agtttttatt
7957-3 aatttctgaa ggatcatgtg acgcttaaga ctggagtaat gatgctaaaa attcagcttt acattacaag aataaattac attttaataa agtttttatt
7957-5 aatttctgaa ggatcatgag acacttaaga ctggagtaat gatgctgaaa attcagcttt acattacaag aataaactac attttaataa agtttttatt
7957-6 aatttctgaa ggatcatgtg acacttagga ctggagtaat gatgctgaaa attcagcttt acattacaag aataaactac attttaataa agtttttatt
7957-1 ---------- ---------- ---------- ---------- ---------- ---------- ---------- ---tagctac attttaataa agtttttatt

        4444444444 4444444444 4444444444 4444444444 4444444444 4444444444 4444444444 4444444444 4444444444 4444444445
        0000000001 1111111112 2222222223 3333333334 4444444445 5555555556 6666666667 7777777778 8888888889 9999999990
        1234567890 1234567890 1234567890 1234567890 1234567890 1234567890 1234567890 1234567890 1234567890 1234567890
7957-4 attgtaataa tatttcacag tattactgtt tt--aatgta tctttgatca aataaatgca gccttgaggt tgataagagc agaacc-aac cacacctgga
7957-7 attgtaataa tatttcacag tattactgtt ttt-agtgta tctttgatca aataaatgca gccttgaggt tgataagagc agaacc-aac cacacctgga
7957-2 attgtaataa tatttcacag tattactgtt ttt-agtgta tctttgatca aataaatgca gccttgaggt tgataagagc agaacc-aac cacacctaga
7957-3 attgtaataa tatttcatag tattactgtt ttt-agtgta tctttgatca aataaatgca gccttgaggt tgataagagc agaacc-aac cacacctaga
7957-5 attgtaataa tatttcacag tattaatgtt ttt-agtgta tctttgatca aataaatgca gccttgaggt tgataagagc agaacc-aac cacacctgga
7957-6 attgtaataa tatttcacag tattactgtt ttt-agtgta tctttgatca aataaatgca gccttgaggt tgataagagc agaacc-aac cacacctgga
7957-1 at-gtaataa tatttcacag tattactgtt ttttagtgta tctt-gatca aataaatgca gcgt-gaggt -gataagagc agaacccaac cacacctgga

        5555555555 5555555555 5555555555 5555555555 5555555555 5555555555 5555555555 5555555555 5555555555 5555555556
        0000000001 1111111112 2222222223 3333333334 4444444445 5555555556 6666666667 7777777778 8888888889 9999999990
        1234567890 1234567890 1234567890 1234567890 1234567890 1234567890 1234567890 1234567890 1234567890 1234567890
7957-4 ctattagcaa tgctgccatg actgtgtgat ctaaagcagg ttgtctttgt ctcttttaca ggttgacaaa ggtgtcgtcc ctctggcagg aacaaatgga
7957-7 ctattagcaa tgctgccatg acagtgtgat ctaaagcagg ttgtctttgt ctcttttaca ggttgacaaa ggtgtcgtcc ctctggcagg aacaaatgga
7957-2 ctattagcaa tgctgccatg acagtgtgat ctaaagcagg ttgtctttgt ctcttttaca ggttgacaaa ggtgtcgtcc ctctggcagg aacaaatgga
7957-3 ctattagcaa tgctgccatg acagtgtgat ctaaagcagg ttgtctttgt ctcttttaca ggttgacaaa ggtgtcgtcc ctctggcagg aacaaatgga
```

```
7957-5 ctattagcaa tgctgccatg acagtgtgat ctaaagcagg ttgtctttgt ctcttttaca ggttgacata ggtgtcgtcc ctctggcagg aacaaatgga
7957-6 ctattagcaa tgctgccatg acagtgtgat ctaaagcagg ttgtctttgt cgcttttaca ggttgacaaa ggtgtcgtcc ctctggcagg aacaaatgga
7957-1 ctattagcaa tgctgccatg acagtgtgat ctaaagcagg ttgtc----- ctcttttaca ggttgacaaa ggtgtcgtcc ctctggcagg aacaaatgga

       6666666666 6666666666 6666666666 6666666666 6666666666 6666666666 6666666666 6666666666 6666666666 6666666667
       0000000001 1111111112 2222222223 3333333334 4444444445 5555555556 6666666667 7777777778 8888888889 9999999990
       1234567890 1234567890 1234567890 1234567890 1234567890 1234567890 1234567890 1234567890 1234567890 1234567890
7957-4 gagaccacaa cacagggtaa gcttagtcac tgatacagta tttgctaaaa ttgatggcaa tgatgttttg ttaacggggt gacctgtttg ttttaagggc
7957-7 gagaccacaa cacagggtaa gcttagtcac tgatacagta tttgctaaaa ttgatggcaa tgatgttttg ttaatggggt gacctgtttg ttttaagggc
7957-2 gagaccacaa cacagggtaa gcttagtcac tgatacagta tttgctaaaa ttgatggcaa tgatgttttg ttaacggggt gacctgtttg ttttaagggc
7957-3 gagaccacaa cacagggtaa gcttagtcac tgatactgta tttgctaaaa ttgatggcaa tgatgttttg ttaacggggt gacctgtttg ttttaagggc
7957-5 gagaccacaa cacagggtaa gcttagtcac tgatacagta tttgctaaaa ttgatggcaa tgatgttttg ttaatggggt gacctgtttg ttttaagggc
7957-6 gagaccacaa cacagggtaa gcttagtcac tgatacagta tttgctaaaa ttgatggcaa tgatgttttg ttaactgggt gacctgtttg ttttaagggc
7957-1 gagaccacaa cacagggtaa gcttagtcac tgatacagta tttgctaaaa ttgatggcaa tgatgttttg ttaatggggt gacctgtttg ttttaagggc

       7777777777 7777777777 7777777777 7777777777 7777777777 7777777777 7777777777 7777777777 7777777777 7777777778
       0000000001 1111111112 2222222223 3333333334 4444444445 5555555556 6666666667 7777777778 8888888889 9999999990
       1234567890 1234567890 1234567890 1234567890 1234567890 1234567890 1234567890 1234567890 1234567890 1234567890
7957-4 ttgatggcct gtcagaacgc tgtgctcagt ataagaaaga tggtgcagac tttgccaaat ggcggtctgt actgaagatc agtgagacca ctccttcaga
7957-7 ttgatggcct gtcagaacgc tgtgctcagt ataagaaaga tggtgcagac tttgccaaat ggcggtctgt actgaagatc agtgagacca ctccttcaga
7957-2 ttgatggcct gtcagaacgc tgtgctcagt ataagaaaga tggtgcagac tttgccaagt ggcggtctgt actgaagatc agtgagacca ctccttcaga
7957-3 ttgatggcct gtcagaacgc tgtgctcagt ataagaaaga tggtgcagac tttgccaagt ggtggtctgt actgaagatc agtgagacca ctccttcaga
7957-5 ttgatggcct gtcagaacgc tgtgctcagt ataagaaaga tggtgcagac tttgccaagt ggcggtctgt actgaagatc agcgagacca ctccttcaga
7957-6 ttgatggcct gtcagaacgc tgtgctcagt ataagaaaga tggtgcagac tttgccaaat ggcggtctgt actgaagatc agtgagacca ctccttcaga
7957-1 ttgatggcct gtcagaacgc tgtgctcagt ataggaaaga tggtgcagac tttgccaagt ggcggtctgt actgaagatc agcgagacca ctccttcaga

       8888888888 8888888888 8888888888 8888888888 8888888888 8888888888 8888888888 8888888888 8888888888 8888888889
       0000000001 1111111112 2222222223 3333333334 4444444445 5555555556 6666666667 7777777778 8888888889 9999999990
       1234567890 1234567890 1234567890 1234567890 1234567890 1234567890 1234567890 1234567890 1234567890 1234567890
7957-4 gttagccatc atggagaacg ctaacgtcct ggctcgctac gccagcatct gccagcaggt gagctcatta a-tcttgcgc ggtctttgtg tttgccttaa
```

```
7957-7 gttagccatc atggagaatg ctaacgtcct ggctcgctac gccagcatct gccagcaggt gagctcatca agtcttgcgc ggtctttgtg tttgccttaa
7957-2 gatagccatc atggagaatg ctaacgtcct ggctcgctac gccagcatct gccagcaggt gagctcatta a-tcttgcgc ggtctttgtg tttgccttaa
7957-3 gttagccatc atggagaatg ctaacgtcct ggctcgctac gccagcatct gccagcaggt gagctcatta agtcttgggc ggtctttgcg tttgtcttaa
7957-5 gttagccatc atggagaatg ctaacgtcct ggctcgctac gccagcatct gccagcasgt gaactcatta agtcttgggc ggtctttgcg tttgtcttaa
7957-6 gttagccatc atggagaatg ctaacgtcct ggctcgctac gccagcatct gccagcaggt aagctcatta agtct-aggt ggtctttgcg tttgtcttaa
7957-1 gttagccatc atggagaatg ctaacgtcct ggctcgctac gccagcatct gccagcaggt gagctcatta agtct-aggt ggtccttgcg tttgtcttaa

                                                                                                                  1
       9999999999 9999999999 9999999999 9999999999 9999999999 9999999999 9999999999 9999999999 9999999999 9999999990
       0000000001 1111111112 2222222223 3333333334 4444444445 5555555556 6666666667 7777777778 8888888889 9999999990
       1234567890 1234567890 1234567890 1234567890 1234567890 1234567890 1234567890 1234567890 1234567890 1234567890
7957-4 aggcaagcag tgttgttatt gttaactaaa actaaaacta ttaaaagtta t---ctgaat tgttaattga agcagaaatt aaatcgaaat attacatgaa
7957-7 aggcaagcag tgttgttatt gttaactaaa actaaaacta ttataagtta t---ctgaat tgttaattga agcagaaatt aaatcgaaat attacatgaa
7957-2 aggcaagcag tgtcgttatt gttaactaaa actaaaacta ttaaaagtta ttatctgaat tgttaattga agcagaaatt aaatcgaaat attacataaa
7957-3 aggcaagcag tgttgttatt gttaactaaa actaaaacta ttaaaagttg atatctgaat tgttaattga agcagaaatt aaatcgaaat attacatgaa
7957-5 aggcaagcag tgttgttatt gttaactaaa actaaaacta ttaaaagttg ttatctgaat tgttaattga agcagaaatt aaatcgaaat attacatgaa
7957-6 aggcaagcaa tgttgttatt gttaactaaa ------acta ttaaaagttg ttatctcaat tgttaattga agcagaaatt aaatcgaaat attacatgaa
7957-1 aggcaagcaa tgttgttatt gttaactaaa ------acta ttaaaagttg ttatcttaat tgttaattga agcagaaatt aaatcgaaat attacatgaa

       1111111111 1111111111 1111111111 1111111111 1111111111 1111111111 1111111111 1111111111 1111111111 1111111111
       0000000000 0000000000 0000000000 0000000000 0000000000 0000000000 0000000000 0000000000 0000000000 0000000001
       0000000001 1111111112 2222222223 3333333334 4444444445 5555555556 6666666667 7777777778 8888888889 9999999990
       1234567890 1234567890 1234567890 1234567890 1234567890 1234567890 1234567890 1234567890 1234567890 1234567890
7957-4 aaa-cctata aaaaacaaaa ttgcaacgtt gcaatgaaaa ctagctgaat tcaaataagc tgaagtacta aactgacaaa ctaaaactga aataaaaata
7957-7 aaa-cctata aaaaacaaaa ttgcaacgtt gcaatgaaaa ctagctgaat tcaaataagc tgaagtacta aactgacaaa ctaaaactga aataaaaata
7957-2 aaa-cctata aaaaacaaaa ttgcaacgtt gcaatgaaaa ctagctgaat tcaaataagc tgaagtacta aactgacaaa ctaaaactga aataaaaata
7957-3 aaa-cctata aaaaacaaaa ttgcaacatt gcaatgaaaa ctagctgaat tcaaataagc tgaagtacta aactgacaaa ctaaaactga aataaaaata
7957-5 aaa-cctata aaaaacaaaa ttgcaacgtt acaatgaaaa ctagctgaat tcaaataagc tgaagtacta aactgacaaa ctaaaactga aataaaaata
7957-6 aaa-cctata aaaaacaaaa ttgcaacgtt gcaatgaaaa ctagctgaat tcaaataagt tgaagtacta aactgacaaa ctaaaactga aataaaaata
7957-1 aaaacctata aaaaacaaaa ttgcaacgtt gcaatgaaaa ctagctgaat tcaaataagc tgaagtacta aactgacaaa ctaaaactga aataaaaata
```

```
        1111111111 1111111111 1111111111 1111111111 1111111111 1111111111 1111111111 1111111111 1111111111 1111111111
        1111111111 1111111111 1111111111 1111111111 1111111111 1111111111 1111111111 1111111111 1111111111 1111111112
        0000000001 1111111112 2222222223 3333333334 4444444445 5555555556 6666666667 7777777778 8888888889 9999999990
        1234567890 1234567890 1234567890 1234567890 1234567890 1234567890 1234567890 1234567890 1234567890 1234567890
7957-4  ataaagctaa atagaagtat ttaaaaaaaa a-tggcaaaa gcacatatga ctaaaactta aactaaaatt taaaattgaa aaataaaagt aatagtacat
7957-7  ataaagctaa atagaaatat taaaaaaaaa aatggcaaaa gcacatatga ctaaaactta aactaaaatt gaaaattgaa aaataaaagt aatagtacat
7957-2  atgaagctaa atagaaatat -----aaaaa aatggcaaaa gcacatatga ctaaaactta aactaaaa-- -----ttgaa aaataaaagt aatagtacat
7957-3  atgaagctaa atagaaatat -----aaaaa aatggcaaaa gcacatatga ctaaaactta aactaaaa-- -----ttgaa aaataaaagt aatagtacat
7957-5  atgaagctaa atagaaatat -----aagaa aatggcaaaa gcacatatga ctaaaactta aactaaaatt taaaattgaa aaataaaagt aatagtacat
7957-6  ataaagctaa atagaaatat ttaa-aaaaa aatggcaaaa gcacatatga ctaaaactta aactaaaatt taaaattgaa aaataaaagt aatagtacat
7957-1  ataaagctaa atagaaatat tttt-taaaa aatggcaaaa gcacatatga ctaaaactta aactaaaatt taaaattgaa aaataaaagt aatagtacat

        1111111111 1111111111 1111111111 1111111111 1111111111 1111111111 1111111111 1111111111 1111111111 1111111111
        2222222222 2222222222 2222222222 2222222222 2222222222 2222222222 2222222222 2222222222 2222222222 2222222223
        0000000001 1111111112 2222222223 3333333334 4444444445 5555555556 6666666667 7777777778 8888888889 9999999990
        1234567890 1234567890 1234567890 1234567890 1234567890 1234567890 1234567890 1234567890 1234567890 1234567890
7957-4  tgaaattatg catgaatact ataatggtat ataaataata ccaataaaac tgaacactga aatcacaaag gtcttgatgt gtatttagat attttagaga
7957-7  tgaaattatg catgaatact ataatggtat ataaa---ta tcaataaaac tgaacactga aatcacaaag gtcttgatgt gtatttagat attttagaga
7957-2  tgaaattatg catgaatact ataatggtat ataaataata tcaataaaac tgaacactga aatcacaaag gtcttgatgt gtatttagat attttagaga
7957-3  tgaaattatg catgaatact ataatggtat ataaataata ccaataaaac tgaacactga aatcacaaag gtcttgatgt gtatttagat attttagaga
7957-5  tgaaattatg catgaatact ataatggtat ataaataaca tcaataaaac tgaacactga aatcacaaag gtcttgatgt gtatttagat attttagaga
7957-6  tgaaattatg catgaatact ataatggtat ataaataata tcaataaaac tgaacactga aatcacaaag gtcttgatgt gtatttagat attttagaga
7957-1  tgaaattatg catgaatact ataatggtat ataaataata ccaataaaac tgaacactga aatcacaaag gtcttgatgt gtatttagat attttagaga

        1111111111 1111111111 1111111111 1111111111 1111111111 1111111111 1111111111 1111111111 1111111111 1111111111
        3333333333 3333333333 3333333333 3333333333 3333333333 3333333333 3333333333 3333333333 3333333333 3333333334
        0000000001 1111111112 2222222223 3333333334 4444444445 5555555556 6666666667 7777777778 8888888889 9999999990
        1234567890 1234567890 1234567890 1234567890 1234567890 1234567890 1234567890 1234567890 1234567890 1234567890
7957-4  attgttggat ggagttgaag ctaatgcata gatatcagag ataagatgga tgcagtgtga cttttttgga cacaatattg tctgtttggt ttttcagaat
```

```
7957-7 attgttggtt ggaattgaag ctaatgcata gatatcagag ataagatgga tgcagtgtga cttttttga cacaatattg tctgtttggt ttttcagaat
7957-2 attgttggtt ggaattgaag ctaatgcata catatcagag ataagatgga tgcagtgtga ctttttt-ga cacaatattg tctgtttggt ttttcagaat
7957-3 attgttggat ggagttgaag ctaatgcata gatatcagag ataagatgga tgcagtgtga cttttttga cacaatattg tctgtttggt ttttcagaat
7957-5 attgttggtt ggaattgaag ctaatgcata catatcagag ataagatgga tgcagtgtga cttttttga cacaatattg tctgtttggt ttttcagaat
7957-6 attgttggtt ggaattgaag ctaatgcata gatatcagag ataagatgga tgcagtgtga cttttttga cacaatattg tctgtttggt ttttcagaat
7957-1 attgttggtt ggaattgaag ctaatgcata gatatcagag ataagatgga tgcagtgtga cttttttcga cacaatattg tctgtttggt ttttcagaat

       1111111111 1111111111 1111111111 1111111111 11111
       4444444444 4444444444 4444444444 4444444444 44444
       0000000001 1111111112 2222222223 3333333334 44444
       1234567890 1234567890 1234567890 1234567890 12345
7957-4 gggattgtgc ccattgttga gc-agagatt ctacctgatg gggac
7957-7 gggattgtgc ccattgttga gccagagatt ctacctgatg gggac
7957-2 gggattgtgc ccattgttga gccagagatt ctacctgatg gggac
7957-3 ggaattgtgc ccattgttga gccagagatt ctacctgatg gggac
7957-5 gggattgtgc ccattgttga gccagagatt ctacctgatg gggac
7957-6 gggattgtgc ccattgttga gccagagatt ctacctgatg gggac
7957-1 gggattgtgc ccattgttga gccagagatt ctacctgatg gggac
```

SNP LOCATIONS: 100A/C, 498G/A, 675C/T, 759G/A, 783T/C, 877G/A, 878G/C, 880C/T, 889C/T, 895C/T, 910G/A, 950G/A, 1103G/A, 1241T/C, 1309T/A, 1314A/G, 1331G/C

22.

```
                    1 1111111112 2222222223 3333333334 4444444445 5555555556 6666666667 7777777778 8888888889 9999999990
           1234567890 1234567890 1234567890 1234567890 1234567890 1234567890 1234567890 1234567890 1234567890 1234567890
    7961-3 actttccaga agacgagcac catcagagtt tcacctgatg agctgaagaa taaccagttc agccgtgtgg tggagcatca ggccgaaacc atcagaatca
    7961-5 actttccaga agacgagcac catcagagtt tcacctgatg agctgaagga taaccagttc agctgtgtgg tggagcatca gggcgaaacc atcagaatca
    7961-4 actttccaga agacgagcac catcagggtt acaccagagg agtggaacaa caacaagttc agctgtgtgg tggagcatca gggcaaaacc ttcagaggga
    7961-1 actttccaga agacgagcac catcacaatt acacctgaag agttgaagaa caaccagtac agctgtgtgg tggagcacca gggcaaaacc atcagaga--
    7961-2 actttccaga agacgagcac catcacaatt acagctgatg accgaaagaa gaacgaatac acctgtgtgg tggagcacca gggtaaaacc atcagagaga

           1111111111 1111111111 1111111111 1111111111 1111111111 1111111111 1111111111 1111111111 1111111111 1111111112
           0000000001 1111111112 2222222223 3333333334 4444444445 5555555556 6666666667 7777777778 8888888889 9999999990
           1234567890 1234567890 1234567890 1234567890 1234567890 1234567890 1234567890 1234567890 1234567890 1234567890
    7961-3 ttctgacaga tggtaagatt cacagattca ctattgtaag cgtgattgtc t--tgagtag atcttgaacc taaaaaatgt taatggaaat catttgctat
    7961-5 ttctgacaga cggtaagatt cacagattca ctattgtaag cgtgattgtc t--tgagtag atcttgaacc ttataaatgt taatggaaat catttgctat
    7961-4 ttctgacaga cggtaagatt cacggattca cttttgtaag cgtgattgtc t--tgagtag atcttgaacc taaaaaatgt taatggaaat catttgctat
    7961-1 tcctgaaaga cggtaagatc --cgaattta -tatgttaag aatgatgtct tgataaa-aa atctta---- ---------- --atggaaat catgtgctat
    7961-2 tcctgacaga cagtaagatc --ctaattca -tatgttaag agtgatgtct tgatgagtag atcatggaac taataaatgt t-attgaaat catgtgctat

           2222222222 2222222222 2222222222 2222222222 2222222222 2222222222 2222222222 2222222222 2222222222 2222222223
           0000000001 1111111112 2222222223 3333333334 4444444445 5555555556 6666666667 7777777778 8888888889 9999999990
           1234567890 1234567890 1234567890 1234567890 1234567890 1234567890 1234567890 1234567890 1234567890 1234567890
    7961-3 tgccagtctc tgattagtct gcactaatgt actgcagata tcacaatcat agatgctgtt tttgtatctg ctgaaatctg aaacctttac cagtttctaa
    7961-5 tgtcagtctc tgattagtct gcactaatgt actgcagata tcacaatcat agatgctgtt tttgtatctg ctgaaatctg aaacctttac cagtttctaa
    7961-4 tgccagtctc tgattagtct gcactaatgt actgcagata tcacaatcat gaatgctgtt tttgtatctg ctgaaatctg aaacctttac cagtttctaa
    7961-1 ttccagtctc tgattagact gcactaatgt actgatgata tcacagtcat atgttgt-tt tttgtatctg ctgaaatttg aaaccttgac cagtttctaa
    7961-2 ttccagtctc tgattagact gcactaatgt actgatggta tcacagtcat atgttgt-tt tttgtatctg ctgaaatttt aaacctgtcc cagtttctaa

           3333333333 3333333333 3333333333 3333333333 3333333333 3333333333 3333333333 3333333333 3333333333 3333333334
           0000000001 1111111112 2222222223 3333333334 4444444445 5555555556 6666666667 7777777778 8888888889 9999999990
           1234567890 1234567890 1234567890 1234567890 1234567890 1234567890 1234567890 1234567890 1234567890 1234567890
```

```
7961-3 gattatcaga gtctttaata accggatgtt ttcatttgat cagattcttc agcttctgtc cccatcggca tcattcttgg tgctgttgct ggtgtcctcc
7961-5 gattatcaga gtctttaata accagatgtt ttcatttgat catattcttc agcttctgtc cccatcggca tcattcttgg tgctgttgct ggtgtcctcc
7961-4 gattatcaga gtctttaata accggatgtt ttcatttga- ----ttcttc agcttctgtc cccatcggca tcattcttgg tgctgttgct ggtgtcctcc
7961-1 gattgtcaga atctttaata agctgatgtt ttcacttgat ctgattcttc agattctgtc cccattggca tcatcattgg tgttgttgct gctgtcctcc
7961-2 gattgtcaga atctttaata agctgatgtt ttcacttgat ctgattcttc agattctgtc cccatcggca tcattgttgg tgttgttgct gctgtcctcc

       4444444444 4444444444 4444444444 4444444444 4
       0000000001 1111111112 2222222223 3333333334 4
       1234567890 1234567890 1234567890 1234567890 1
7961-3 tgttgattgt cactgctgtt gctgggtata aggtctatca g
7961-5 tgttgattgt cactgctgtc gctgggtata aggtctatca g
7961-4 tgttgattgt cactgctgtt gctgggtata aggtctatca g
7961-1 tgttgattgt cagtgctgtt gctgggtata aggtctatca g
7961-2 tgttgattgt cattgctgtc gctgggtata aggtctatca g
```

SNP LOCATIONS: 26G/C, 28G/A, 31A/T, 43C/T, 51T/C, 59T/A, 78T/C, 85A/G, 97G/A, 98A/T, 99C/G, 102T/C, 120T/C, 124A/G, 125A/G, 135T/G, 136G/T, 141C/A, 147T/G, 148G/T, 149T/C, 150C/T, 174A/T, 194T/G, 202G/T, 218T/A, 235C/A, 236A/T, 246A/G, 252G/T, 253A/G, 255G/T, 256C/G, 278C/T, 305A/G, 311G/A, 322C//G, 324T/G, 335T/C, 353C/A, 383C/T, 392G/C, 420T/C

23.

```
                1 1111111112 2222222223 3333333334 4444444445 5555555556 6666666667 7777777778 8888888889 9999999990
       1234567890 1234567890 1234567890 1234567890 1234567890 1234567890 1234567890 1234567890 1234567890 1234567890
7969-3 tggatttaga gaacgaaaca gcaaccttca tgtgtttagc ccaacggttt tcgcctaaat cacacacgtt taagtggttt ctggatggta acgagctgaa
7969-5 tggatttaga gaacgaaaca gcaaccttca tgtgtttagc ccaacggttt tcgcctaaat cacacacgtt taagtggttt ctggatggta acgagctgaa
7969-2 tggatttaga gaacgaaaca gcaaccttca tgtgtttagc ccaacggttt tcgcctaaat cacacacgtt taagtggttt ctggatggta acgagctgaa
7969-4 tggatttaga gaacgaaaca gcaaccttca tatgtttagc ccaacggttt tcgcctaaat cacacacgtt taagtggttt ctggatggta acgagctgaa
7969-0 tggatttaga gaacgaaaca gcaaccttca tgtgtttagc ccaacggttt tcgcctaaat cacacacgtt taagtggttt ctggatggta acgagctgaa
```

```
7969-1 tggatttaga gaacgaaaca gcaaccttca tgtgtttagc ccaacggttt tcgcctaaat tatacacgtt taagtggtct ctggatgata aagaggtgaa
7969-6 tggatttaga gaacgaaaca gcaaccttca tgtgtttagc ccaacggttt tcgcctaaat tatacacgtt taagtggtct ctggatgata aagaggtgaa

       1111111111 1111111111 1111111111 1111111111 1111111111 1111111111 1111111111 1111111111 1111111111 1111111112
       0000000001 1111111112 2222222223 3333333334 4444444445 5555555556 6666666667 7777777778 8888888889 9999999990
       1234567890 1234567890 1234567890 1234567890 1234567890 1234567890 1234567890 1234567890 1234567890 1234567890
7969-3 aaaaacgata gaaaattatg acaaaagtga gaagaaaggc tcagtaaccg aatatagtgc aacaagcatt ttgcaaatca gtgccgaagc ctggaagaag
7969-5 caaaacgata gaaaattatg acaaaagtga gaagaaaggc tcagtaaccg aatatagtgc aacaagcatt ttgcaaatca gtgccgaagc ctggaagaag
7969-2 aaaaacgata gaaaattatg acaaaagtga gaagaaaggc tcagtaaccg aatatagtgc aacaagcatt ttgcaaatca gtgccgaagc ctggaagaag
7969-4 aaaaacgata gaaaattatg acaaaagtga gaagaaaggc tcagtaaccg aatatagtgc aacaagcatt ttgcaaatca gtgccgaagc ctggaagaag
7969-0 aaaaacgata gaaaattatg acaaaagtga gaagaaaggc tcagtaaccg aatatagtgc aacaagcatt ttgcaaatca gtgccgaagc ctggaagaag
7969-1 agaaacgata gaaagttatg acaaaagtga gaagaaaggc tcagtaaccg aatatagtgc cacaagcatt ttgcaaatca aggccgaagc ctggaagaag
7969-6 agaaacgata gaaagttatg acaaaagtga gaagaaaggc tcagtaaccg aatatagtgc cacaagcatt ttgcaaatca aggccgaagc ctggaagaag

       2222222222 222222
       0000000001 111111
       1234567890 123456
7969-3 caaaccagca aagtta
7969-5 caaaccagca aagtta
7969-2 caaaccagca aagtta
7969-4 caaaccagca aagtta
7969-0 caaaccagca aagtta
7969-1 caaaccagca aagtta
7969-6 caaaccagca aagtta
```

SNP LOCATIONS: 61C/T, 63C/T, 79T/C, 88G/A, 92C/A, 96C/G, 102A/G, 115A/G, 161A/C, 181G/A, 182T/G

24.

```
                1 1111111112 2222222223 3333333334 4444444445 5555555556 6666666667 7777777778 8888888889 9999999990
       1234567890 1234567890 1234567890 1234567890 1234567890 1234567890 1234567890 1234567890 1234567890 1234567890
```

7974-4 gagcgtccat cagaaagaat gtaaactcca agagaagcca gtaagtattc actatcagag aagaattgtc ataatgcctg tagaagtcat tgtagaaaaa
7974-5 gagcgtccat cagaaagaat gtaaactcca agagaagcca gtaagtattc actatcagaa aagaattgtc ataatgcctg tagaagtcat tgtagaaaaa
7974-3 gagcgtccat cagaaagaat gtaaactcca agagaagcca gtaagtattc actatcagag aagaattgtc ataatgcctg tagaagtcat tgtagaaaaa
7974-6 gagcgtccat cagaaagaat gtaaactcca agagaagcca gtaagtattc actatcagag aagaattgtc aaaatgcctg tagaagtcat tgtagaaaaa
7974-1 gagcgtccat cagaaagaat gtaaactcca agagaagcca gtaagtattc actatcagag aagaattgtc aaaatgcctg tagaagtcat tgtagaaaaa
7974-2 gagcgtccat cagaaagaat gtaaactcca cgagaaggca gtaagtattc actatcagag aagaattgtc aaaatgcctg tagaagtcat tgtagaaaaa

1111111111 1111111111 1111111111 1111111111 1111111111 1111111111 1111111111 1111111111 1111111111 1111111112
0000000001 1111111112 2222222223 3333333334 4444444445 5555555556 6666666667 7777777778 8888888889 9999999990
1234567890 1234567890 1234567890 1234567890 1234567890 1234567890 1234567890 1234567890 1234567890 1234567890
7974-4 cctgacagat tcacacatct ctatggtgct tttctcagcc tcaggtttcc tgtattgact gtcaaggaat catggaacca tgtttacttc tcagacatat
7974-5 cctgacagat tcacacatct ctatggtgct tttctcagcc tcaggtttcc tgtattgact gtcaaggaat catggaacca tgtttacttc tcagacatat
7974-3 cctgacagat tcacacatct ctatggtgct tttctcagcc tcaggtttcc tgtattgact gtcaaggaat catggaacca tgtttacttc tcagacatat
7974-6 cctgacagat tcacacattt ctatggtgct tttctcagcc tcaggtttcc tgtattgact gtcaaggaat catggaacca tgtttacttc tcagacatat
7974-1 cctgacagtt tcacacattt ctatggtgct tttctcagcc tcaggtttcc tgtattgact gtaaaggaat catggaacca tgtttgcttc tcagacaaaa
7974-2 cctgacagtt tcacacattt ctatggtgct tttctcagcc tcaggtttcc tgtattgact gtaaaggaat catgaagcca tgtttacttc tcagatataa

2222222222 2222222222 2222222222 2222222222 2222222222 2222222222 2222222222 2222222222 2222222222 2222222223
0000000001 1111111112 2222222223 3333333334 4444444445 5555555556 6666666667 7777777778 8888888889 9999999990
1234567890 1234567890 1234567890 1234567890 1234567890 1234567890 1234567890 1234567890 1234567890 1234567890
7974-4 ggacgaggta aatcacatat tactatatta gtccatggag acataaacct cctacattca cagaacacca tcatgatcca tcctgtatgt tgtcaagata
7974-5 ggacgaggta aatcacatat tactatatta gtccatggag acataaacct cctacattca cagaacacca tcatgatcca tcctgtatgt tgtcaagata
7974-3 ggacgaggta aatcacatat tactatatta gtccatggag acataaacct cctacattca cagaacacca tcatgatcca tcctgtatgt tgtcaagata
7974-6 ggacgaggta aatcacatat tactatatta gtccatggag acataaacct cctacattca cagaacacca tcatgatcca tcctgtatgt tgtcaagata
7974-1 ggaagaggta aatcacatat tactatatta gtccatggag acataaacct cctacattca cagaacacca gcatgatcca tcctgtatgt tgtcaagata
7974-2 ggacgaggta aatcacatat tactatatca gtccatgggg acatacacct cctacattca cagaacacca gcatgatcca tcctgaatgt tgtcaagata

3333333333 3333333333 3333333333 3333333333 3333333333 3333333333 3333333333 3333333333 3333333333 3333333334
0000000001 1111111112 2222222223 3333333334 4444444445 5555555556 6666666667 7777777778 8888888889 9999999990
1234567890 1234567890 1234567890 1234567890 1234567890 1234567890 1234567890 1234567890 1234567890 1234567890

```
7974-4 ctcctgcact aaaaggttct tactgttctt attgcagatt aaaaaaagga tggatgaatg taccctgaca gaagataatc atttcactgg gggaggacat
7974-5 ttcctgcact aaaaggttct tactgttctt attgcagatt aaaaaaagga tggatgaatg taccctgaca gaagataatc atttcactgg gggaggacat
7974-3 ttcctgcact aaaaggttct tactgttctt actgcagatt aaaaaaagga tggatgaatg taccctgaca gaagataatc atttcactgg gggaggacat
7974-6 ttcctgcact aaaaggtact tactgttctt attgcagatt aaaaaaagga tggatgaatg taccctgaca gaagataatc atttcactgg gggaggacat
7974-1 ttcctgcact aaaagattct tactgttctt attgcagatt aaaaaaagga tggatgaatg taccctgaca gaagataatc atttcactgg gggagggcat
7974-2 ttcctgcaat aaaaggttct tactgttctt attgcagatt aaaaaaaggg tggatgaatg ccccccgaaa gaagatattc atcgtactgg gtcagcacat

       4444444444 4444444444 4444444444 4444444444 444
       0000000001 1111111112 2222222223 3333333334 444
       1234567890 1234567890 1234567890 1234567890 123
7974-4 tcacggttct aaaaatcagg aaatggacac cagcaacaaa ctg
7974-5 tcacggttct aaaaatcagg aaatggacac cagcaacaaa ctg
7974-3 tcacggttct aaaaatcagg aaatggacac cagcaacaaa ctg
7974-6 tcacggttct aaaaatcagg aaatggacac cagcaacaaa ctg
7974-1 tcacggttct aaaaatcagg aaatggacac cagcaacaaa ctg
7974-2 gcaatattcc aaaaatcagg aaatggacac cagcaacaaa ctg
```

SNP LOCATIONS: 72A/T, 109A/T, 109C/T, 163C/A, 200T/A, 271T/G

25.

```
                    1 1111111112 2222222223 3333333334 4444444445 5555555556 6666666667 7777777778 8888888889 9999999990
         1234567890 1234567890 1234567890 1234567890 1234567890 1234567890 1234567890 1234567890 1234567890 1234567890
7980-2-1 gttgtcatcc ctgctggtgt cccaaggaag cctggtgggt atattttgcc tctgctttat ttttaagtgc attaaagtcg gcatggaatg gcatttgtaa
7980-2-3 gttgtcatcc ctgctggtgt cccaaggaag cctggtgggt gtattttgcc tctgctttat ttttaagtgc attaaagtcg gcatggaatg gcatttgtaa
7980-2-5 gttgtcatcc ctgctggtgt cccaaggaag cctggtgggt atattttgcc tctgctttat ttttaagtgc attaaagtcg gcatggaatg gcatttgtaa
7980-2-7 gttgtcatcc ctgctggtgt cccaaggaag cctggtgggt atattttgcc tctgctttat ttttaagtgc attaaagtcg gcatggaatg gcatttgtaa
7980-2-2 gttgtcatcc ctgctggtgt cccaaggaag cctggtgggt atattttgcc tctgctttat ttttaagtgc attaaagtcg gcatggaatg gcatttgtaa
7980-2-4 gttgtcatcc ctgctggtgt cccaaggaag cctggtgggt atatttaacc tctggtttat ttttaagtgc attaaagtcc gcatggaatg gcatttgtaa
7980-2-6 gttgtcatcc ctgctggtgt cccaaggaag cctggtgggt atatttaacc tctgctttat ttttaagtcc attaaagtcc gcacggaatg gcatttgtaa
```

```
           1111111111 1111111111 1111111111 1111111111 1111111111 1111111111 1111111111 1111111111 1111111111 1111111112
           0000000001 1111111112 2222222223 3333333334 4444444445 5555555556 6666666667 7777777778 8888888889 9999999990
           1234567890 1234567890 1234567890 1234567890 1234567890 1234567890 1234567890 1234567890 1234567890 1234567890
7980-2-1   cccattttat ttgtgcatat gtgcaatatg tgaaaaattc aacaaaaact gtttattgcc ttaaacatgc aagggcttgg agatgtaaat gggctcgatc
7980-2-3   cccattttat ttgtgcatat gtgcaatatg tgaaaaattc aacaaaaact gtttattgcc ttaaacatgc aagggcttgg ggatgtaaat gggctcgatc
7980-2-5   cccaatttat ttgtgcatat gtgcaatatg tgaaaaattc aacaaaaact gtttattgcc ttaaacatgc aagggcttgg agatgtaaat gggctcgatc
7980-2-7   cccattttat ttgtgcatat gtgcaatatg tgaaaaattc aacaaaaact gtttattgcc ttaaacatgc aagggcttag agatgtaaat gggctcgatc
7980-2-2   cccattttat ttgtgcatat gtgcaatatg tgaaaaattc aacaaaaact gtttattgcc ttgaacatac aagggcttgg agatgtaaat gggctcaatc
7980-2-4   cccattttat ttgtgcatat gtgcaatttg tgaaaaattc aacaaaaact gtttattgcc ttgaacatac aagggcttgg agatgtaaat gggctcaatc
7980-2-6   cccattttgt ttgtgcatat gtgcaatttg tgaaaaatta aacaaaaact gtttattgcc ttgaacatac aagggcttgg agatgtaaat gtgctcaatc

           2222222222 2222222222 2222222222 2222222222 2222222222 2222222222 2222222222 2222222222 2222222222 2222222223
           0000000001 1111111112 2222222223 3333333334 4444444445 5555555556 6666666667 7777777778 8888888889 9999999990
           1234567890 1234567890 1234567890 1234567890 1234567890 1234567890 1234567890 1234567890 1234567890 1234567890
7980-2-1   ataaaataaa atttatagaa acccaccaga ttttctcaag atagtgcttt tcgtcttttg tctaggtatg acccgtgatg atctgtttaa caccaatgcc
7980-2-3   ataaaataaa atttatagaa acccaccaga ttttctcaag atagtgcttt tcgtcttttg tctaggtatg acccgtgatg atctgtttaa caccaatgcc
7980-2-5   ataaaataaa atttatagaa acccaccaga ttttctcaag atagtgcttt tcgtcttttg tctaggtatg acccgtgatg atctgtttaa caccaatgcc
7980-2-7   ataaaataaa atttatagaa acccaccaga ttttctcaag atagtgcttt tcgtcttttg tctaggtatg acccgtgatg atctgtttaa caccaatgcc
7980-2-2   ataaaataaa atttatagaa acccaacaga ttttctcaag atagtgcttt tcgtcttttg tctaggtatg acccgtgatg atctgtttaa caccaatgcc
7980-2-4   ataaagtaaa atttagagaa acccaccaga ttttctcaag atagtgcttt tcgtcttttt tctaggtatg acccgtgatg atctgtttaa caccaatgcc
7980-2-6   ataaaataaa atttagagaa acccaccaga ttttctcaa- atagtgcttt tcgtcttttt tctaggtatg acccgtgatg atctgtttaa caccaatgcc

           3333333333 3333333333 3333333333 3333333333 3333333333 3333333333 3333333333 3333333333 3333333333 3333333334
           0000000001 1111111112 2222222223 3333333334 4444444445 5555555556 6666666667 7777777778 8888888889 9999999990
           1234567890 1234567890 1234567890 1234567890 1234567890 1234567890 1234567890 1234567890 1234567890 1234567890
7980-2-1   accattgtgg ccacattagc tgatgcttgc gcccgtcact gtcctcaggc catgatctgc atcatttcaa acccagtaag tataaggtct caggagcctt
7980-2-3   accattgtgg ccacattagc tgatgcttgc gcccgtcact gtcctcaggc catgatctgc atcatttcaa acccagtaag tataaggtct caggagcctt
7980-2-5   accattgtgg ccacattagc tgatgcttgc gcccgtcact gtcctcaggc catgatctgc atcatttcaa acccagtaag tataggctct caggagcctt
7980-2-7   accattgtgg ccacattagc tgatgcttgt gcccgtcact gtcctcaggc catgatctgc atcatttcaa acccagtaag tataagctct caggagcctt
7980-2-2   accattgtgg ccacattagc tgatgcttgc gcccgtcact gtcctcaggc catgatctgc atcatttcga acccagtaag tataaggtct caggag----
```

7980-2-4 accattgtgg ctacattagc tgatgcttgc gcccgtcact gtcctcaggc catgatctgc atcatttcaa acccagtaag tataagctct caggagcctt
7980-2-6 accattgtgg ccacattagc tgatgcttgc gcccgtcact gtcctcaggc catgatctgc atcatttcaa acccagtaag tataagctct caggagcctt

4444444444 4444444444 4444444444 4444444444 4444444444 4444444444 4444444444 4444444444 4444444444 4444444445
0000000001 1111111112 2222222223 3333333334 4444444445 5555555556 6666666667 7777777778 8888888889 9999999990
1234567890 1234567890 1234567890 1234567890 1234567890 1234567890 1234567890 1234567890 1234567890 1234567890
7980-2-1 gcattgctga gatattactt tgtacctgct caaaggtcac aagggtttat agcactagac atgtctgtaa gataattatc tgttatccat taagataatt
7980-2-3 gcattgctga gattttactt tatacctgct caaaggtcac aagggtttat agcactagac atgtctgtaa gataattatc tgttatccat taagataatt
7980-2-5 gcattgctga gatattactt tatacctgct caaaggtcac aagggtttat agcactagac atgtctgtaa gataataat- -gttatccat taagataatt
7980-2-7 gcattgctga gatattactt tatacctgct caaaggtcac aagggtttat agcactagac atgtctgtaa cataattatc tgttatccat taagataatt
7980-2-2 atattgctga gatattactt tatacctgct caaaggtcac aagggtttat agcactagac atgtctgtaa gataataat- -gttatccat taagataatt
7980-2-4 gcattgctga gatattactt tatacctgct caaaggtcac aagggtttat agcactagac atgtctgtaa gataattatc tgttatccat taagataatt
7980-2-6 gcattgctga gatattactt tatacctgct caaaggtcac aagggtttat agcactagac atgtctgtaa gataattatc tgttatccat taagataatt

5555555555 5555555555 5555555555 5555555555 5555555555 5555555555 5555555555 5555555555 5555555555 5555555556
0000000001 1111111112 2222222223 3333333334 4444444445 5555555556 6666666667 7777777778 8888888889 9999999990
1234567890 1234567890 1234567890 1234567890 1234567890 1234567890 1234567890 1234567890 1234567890 1234567890
7980-2-1 gataaatgtt cctataaaaa aaaaa--tac attccatatt gaactttgat actgtgaatt tcaggtgaac tccaccatcc cgatcacatc agaggtgatg
7980-2-3 gataaatgtt cctataaaaa aaaaaaatac attccatata gaactttgat actgtgaatt tcaggtgaac tccaccatcc cgatcacatc agaggtgatg
7980-2-5 gataaatgtt cctatagaaa aa-----tag attccatatt gaactttgat actgtgaatt tcaggtgaac tccaccatcc cgatcacatc agaggtgatg
7980-2-7 gataaatgtt cctatagaaa aaaa---tac attccatatt gaactttgat actgtgaatt tcaggtgaac tccaccatcc cgatcacatc agaggtgatg
7980-2-2 gataaatgtt cctatagaaa aa-----tag attccatatt gaactttgat actgtgaatt tcaggtgaac tccaccatcc cgatcacatc agaggtgatg
7980-2-4 gataaatgtt cctataaaaa aaaa---tac attccatatt gaactttgat actgtgaatt tcaggtgaac tccaccatcc cgatcacatc agaggtgatg
7980-2-6 gataaatgtt cctataaaaa aaaaaa-tac attccatatt gaactttgat actgtgaatt tcaggtgaac tccaccatcc cgatcacatc agaggtgatg

6666666666 6666666666 6666666666 6
0000000001 1111111112 2222222223 3
1234567890 1234567890 1234567890 1
7980-2-1 aagaaacatg gcgtctacaa ccccaacaga g
7980-2-3 aagaaacatg gcgtctacaa ccccaacaga g

```
7980-2-5 aagaaacatg gcgtctacaa ccccaacaga g
7980-2-7 aagaaacatg gcgtctacaa ccccaacaga g
7980-2-2 aagaaacatg gcgtctacaa ccccaacaga g
7980-2-4 aagaaacatg gcgtctacaa ccccaacaga g
7980-2-6 aagaaacatg gcgtctacaa ccccaacaga g
```

SNP LOCATIONS: 47T/A, 48G/A, 80G/C, 129T/A, 163A/G, 169G/A, 197G/A, 216T/G, 260G/T, 387C/G, 477T/A, 517A/G, 530C/G

26.

```
                  1 1111111112 2222222223 3333333334 4444444445 5555555556 6666666667 7777777778 8888888889 9999999990
         1234567890 1234567890 1234567890 1234567890 1234567890 1234567890 1234567890 1234567890 1234567890 1234567890
7986-2-2 tgaaagcatt tgttgaagcg agtaagtaca ctaataatca atacgctagt ctggcgatat gaatagaata gaatcagcct aatatgagaa cgtacacatt
7986-2-5 tgaaagcatt tgttgaagcg agtaagtaca ctaacaatca atacgctagt ctggcgatat gaatagaata gaatcagctt aatatgagaa cgtacacatt
7986-2-6 tgaaagcatt tgttgaagcg agtaagtaca ctaataatca atacgctagt ctggcgatat taatagaata gaatcagctt aatataagaa cgtacacatt
7986-2-1 tgaaagcatt tgttgaagcg agtaagtaca ctaaaaatca atacgcttgc ctgatgatat aaatagaata gaatcagctt gatatgagga cagac-----
7986-2-3 tgaaagcatt tgttgaagcg agtaagtaca ctaaaaatca atacgcttgc ctgatgatat aaatagaata gaatcatctt gatatgagga cagac-----
7986-2-4 tgaaagcatt tgttgaagcg agtaagtaca ctaataatca atacactagt ctggcgatat aaatagaata gaatcatctt gatatgagga cagac-----

         1111111111 1111111111 1111111111 1111111111 1111111111 1111111111 1111111111 1111111111 1111111111 1111111112
         0000000001 1111111112 2222222223 3333333334 4444444445 5555555556 6666666667 7777777778 8888888889 9999999990
         1234567890 1234567890 1234567890 1234567890 1234567890 1234567890 1234567890 1234567890 1234567890 1234567890
7986-2-2 cctctcttta cattgtctaa attgatgagc gtcactgttt ctcacacttg tgcagtggtc aatgagaacg tcagcctggt catatccaga cagctgttga
7986-2-5 cctctcttta cattgtctaa attgatgagc gtcactgttt ctcacacttg tgcagtggtc aatgagaacg tcagcctggt catatccaga cagctgttga
7986-2-6 cctctcttta catcgtctaa actgatgagc ttcgctgttt ctcacacttg cgcagtggtc aatgagaacg tcagcctggt catatccaga cagctgttga
7986-2-1 ---------- --tcatcta- ---------- -----tgttt ctcacgtttg tgcagtggtc aatgagaacg tcagcctagt catatccaga cagctgttga
7986-2-3 ---------- --tcatcta- ---------- -----tgttt ctcacgcttg tgcagtggtc aatgagaacg tcagcctggt catatccaga cagctgttga
7986-2-4 ---------- --tcatcta- ---------- -----tgttt ctcacgcttg tgcagtggtc aatgagaacg tcagcctggt catatccaga cagctgttga

         2222222222 2222222222 2222222222 2222222222 2222222222 2222222222 2222222222 2222222222 2222222222 2222222223
```

```
          0000000001 1111111112 2222222223 3333333334 4444444445 5555555556 6666666667 7777777778 8888888889 9999999990
          1234567890 1234567890 1234567890 1234567890 1234567890 1234567890 1234567890 1234567890 1234567890 1234567890
7986-2-2 cagactattg cacgcacctc ctaaacctgc cggacggcac tgcgaaagcc gtgtgtcact tcaccctgga gaggatccag cccagagtca tctcatttga
7986-2-5 cagactattg cacgcacctc ctaaacctgc cggacggcac tgcgaaagcc gtgtgtcact tcaccctgga gaagatccag cccagagtca tctcatttga
7986-2-6 cagactattg cacgcacctt ctcaacctgc cggacggcac tgcgaaagcc gtgtgtcact tcaccctgga gaagatccag cccagagtca tctcatttga
7986-2-1 cagacttctg cacgcacctc ctaaacctgc cggacggcac tgcgaaagcc gtgtgtcact tcaccctgga gaagatccag cccagagtca tctcatttga
7986-2-3 cagacttctg cacgcacctc ctaaacctgc cggacggcac tgcgaaagcc gtgtgtcact tcaccctgga gaagatccag cccagagtca tctcatttga
7986-2-4 cagacttctg cacgcacctc ctaaacctgc cggacggcac tgcgaaagcc gtgtgtcact tcaccctgga gaagatccag cccagagtca tctcatttga

          3333333333 3333333333 3333333333 3333333333 3333333333 3333333333 3333333333 3333333333 3333333333 3333333334
          0000000001 1111111112 2222222223 3333333334 4444444445 5555555556 6666666667 7777777778 8888888889 9999999990
          1234567890 1234567890 1234567890 1234567890 1234567890 1234567890 1234567890 1234567890 1234567890 1234567890
7986-2-2 agaacaggta tgtgtcgtga acagtactct cagatccgtg ctggaatcat gtggatcacg tttgagccaa tctcatacac ttacaggtgg cttctatcag
7986-2-5 agaacaggta tgtgtcgtga acagtactct cagatccgtg ctggaatcat gtggatcacg tttgagccaa tctcatacac ttacaggtgg cttctatcag
7986-2-6 agaacaggta tgtgtcgtga acagtactct cagatcagtg ctggaatcat gtggatcatg tttgagccaa tctcatacac ttacaggtgg cttctatcag
7986-2-1 agaacaggtg tgtgtcgtga acagtactct cagatccgtg ctggaatcat at-------- -ttgagccag tctcacacac ttacaggtgg cttctatcag
7986-2-3 agaacaggtg tgtgtcgtga acagtactct cagatccgtg ctggaatcat gt-------- -ttgagccag tctcacacac ttacaggtgg cttctatcag
7986-2-4 agaacaggcg tgtgtcgtga acagtactct cagatccgtg ctggaatcat gt-------- -ttgagccag tctcacacac ttacaggtgg cttctatcag

          4444
          0000
          1234
7986-2-2 acag
7986-2-5 acag
7986-2-6 acag
7986-2-1 acag
7986-2-3 acag
7986-2-4 acag

SNP LOCATIONS: 35T/A, 48A/T, 50T/C, 54G/A, 55C/T, 61A/G, 77G/T, 81A/G, 89A/G, 92A/G, 93T/G, 146G/A, 207A/T, 208C/T, 310A/G, 370A/G, 376C/T
```

27.

```
                 1 1111111112 2222222223 3333333334 4444444445 5555555556 6666666667 7777777778 8888888889 9999999990
        1234567890 1234567890 1234567890 1234567890 1234567890 1234567890 1234567890 1234567890 1234567890 1234567890
7988-2  cccaggaagt attggttaga aggtctctcg aatctccagt catctacaaa gacaactttt agcacaggtt tacaagcact ga-gtgctac aaaagcactt
7988-5  cccaggaagt attggttaga aggtctctcg aatctccagt catctacaaa gacaactttt agcacaggtt tacaagcact ga-gtgctac aaaagcactt
7988-7  cccaggaagt attggttaga aggtctctcg aatctccagt catctacaaa gacaactttt agcacaggtt tacaagcact ga-gtgctac aaaagcactt
7988-6  cccaggaagt attggttaga aggtctctcg aatctccagt catctacaaa gacaactttt agcacaggtt tacaagcact ga-gtgctac aaaagcactt
7988-1  cccaggaagt attggttaga aggtctctcg aattgccagt catctgcaaa gacacctttt agcacaagtt cacaagcact ggcgtgttat gaagctgttt
7988-4  cccaggaagt attggttaga aggtctctcg aattgccagt catctgtaaa gacacctttt agcacaagtt cacaagcact ggtgtgttat gaacctgttt
7988-3  cccaggaagt attggttaga aggtctctcg aattgccagt catctgcaaa gacaccettt agcacaagtt cacaagcact ggtgtgttat gaagctgttt

        1111111111 1111111111 1111111111 1111111111 1111111111 1111111111 1111111111 1111111111 1111111111 1111111112
        0000000001 1111111112 2222222223 3333333334 4444444445 5555555556 6666666667 7777777778 8888888889 9999999990
        1234567890 1234567890 1234567890 1234567890 1234567890 1234567890 1234567890 1234567890 1234567890 1234567890
7988-2  ggtataccaa ccagtcatga gtttgaggga gaagacaaag atctcctctg caactttagt agaggtatat ggggtccaag tgggccccaa ggaatcgctg
7988-5  ggtataccaa ccagtcatga gtttgaggga gaagacaaag atctcctctg caactttagt agaggtatat ggggtccaag tgggccccaa ggaatcgctg
7988-7  ggtataccaa ccagtcatga gtttgaggga gaagacaaag atctcctctg caactttagt agaggtatat ggggtccaag tgggccccaa ggaatcgctg
7988-6  ggtataccaa ccagtcatga gtttgaggga gaagacaaag atctcctctg caactttagt agaggtatat ggggtccaag tgggccccaa ggaatcgctg
7988-1  gatataccaa ccagtcatga gcctgaggga gaacacaaag atctcctctg cagctataga agcggcatat gggatccaat tgggcattaa ggcatcgcta
7988-4  gatataccaa ccagtcatga gcctgaggga gaacacaaag atctcctctg cagctttaga agcggcatat gggatccaat tgggcattaa ggcatcgcta
7988-3  gatataccaa ccagtcatga gcctgaggga gaacacaaag atctcctctg cagctttaga agcggcatat gggatccaat tgggcattaa ggcatcgcta

        2222222222 2222222222 2222222222 2222222222 2222222222 2222222222 2222222222 2222222222 2222222222 2222222223
        0000000001 1111111112 2222222223 3333333334 4444444445 5555555556 6666666667 7777777778 8888888889 9999999990
        1234567890 1234567890 1234567890 1234567890 1234567890 1234567890 1234567890 1234567890 1234567890 1234567890
7988-2  ctcacattat gctttctgca agagaaagaa ataaaccaac accttatggt ggctttagta catatggcaa ccttgaattc tgccctcacc ttgaatagtg
7988-5  ctcacattat gctttctgca agagaattaa ataaaccaac accttatggt ggctttaata catatggcaa ccttgaattc tgccctcacc ttgaatagtg
7988-7  ctcacattat gctttctgca agagaattaa ataaaccaac accttatggt ggctttagta catatggcaa ccttgaattc tgccctcacc ttgaatagtg
7988-6  ctcacattat gctttctgca agagaattaa ataaaccaac accttatggt ggctttagta catatggcaa ccttgaattc tgccctcacc ttgaatagtg
```

```
7988-1 ctcacattat gctttctgca gaggagttag atagacagtc attatatggg g--tttagta catccagtaa ccttgagttc tgccctcacc ttgaatagtg
7988-4 ctcacattat gctttctgca gaggagttag atagacggtc attatatggg g--tttagta catccagtaa ccttgaattc tgccctcacc ttgaatagtg
7988-3 ctcacattat gctttctgca agggagttag atagatggtc attatatggg g--tttagta catccagtaa ccttgaattc tgccctcacc ttgaataatg

       3333333333 3333333333 333333
       0000000001 1111111112 222222
       1234567890 1234567890 123456
7988-2 acactcgata ccaaccaccg cactac
7988-5 acactcgata ccaaccaccg cactac
7988-7 acactcgata ccaaccaccg cactac
7988-6 acactcgata ccaaccaccg cactac
7988-1 acactcgata ccaaccaccg cactac
7988-4 acactcgata ccaaccaccg cactac
7988-3 acactcgata ccaaccaccg cactac
```

SNP LOCATIONS: 35C/T, 36T/G, 46A/G, 55A/C, 67G/A, 71T/C, 82A/G, 87C/T, 90C/T, 91A/G, 94A/G, 95G/C, 96C/T, 97A/G, 98C/T, 102G/A, 122T/C, 123T/C, 134G/C, 152A/G, 160T/A, 163A/C, 166T/C, 174G/A, 180G/T, 186C/A, 187C/T, 188C/T, 193A/C, 200G/A, 221A/G, 222G/A, 223A/G, 226A/G, 230A/G, 234A/G, 237C/G, 238A/G, 239A/T, 242C/T, 243C/T, 244T/A, 250T/G, 264A/C, 265T/C, 266G/A, 268C/T

28.

```
                1 1111111112 2222222223 3333333334 4444444445 5555555556 6666666667 7777777778 8888888889 9999999990
       1234567890 1234567890 1234567890 1234567890 1234567890 1234567890 1234567890 1234567890 1234567890 1234567890
7990-4 cattacagtc accgacaggg tcagggcttg tctgcgttca cgcgtttgac tgccacatca tttttttaga cgctgaatgt aagatttcct ttgacatccc
7990-5 cattacagtc accgacaggg tcagggcttg tctgcgttca cgcgtttgac tgccacatca tttttttaga cgctgaatgt aagatttcct ttgacatccc
7990-3 cattacagtc accgacaggg tcagggcttg tctgcgttca cgcgtttgac tgccacatca tttttttaga cgctgaatgt aagatttcct ttgacatccc
7990-2 cattacagtc accgacaggg tcagggcttg tctgcgttca cgcgtttgac tgccacatca tttttttaga cgctgaatgt aagatttcct ttgacatccc
7990-1 cattacagtc accgacaggg tcagggcttg tctgcgttca cgcgtttgac tgccacatca tttttttagg cgctgaatgt aagatttcct ttgacatccc
7990-6 cattacagtc accgacaggg tcagggcttg tctgcgttca cgcgtttgac tgccacatca tttttttaga cgctgaatgt aagatttcct ttgacatccc

       1111111111 1111111111 1111111111 1111111111 1111111111 1111111111 1111111111 1111111111 1111111111 1111111112
```

```
       0000000001 1111111112 2222222223 3333333334 4444444445 5555555556 6666666667 7777777778 8888888889 9999999990
       1234567890 1234567890 1234567890 1234567890 1234567890 1234567890 1234567890 1234567890 1234567890 1234567890
7990-4 ggcatttt-a atgttagttt ttatgtaggc agctggtatc tctcaggtta gctcacacat tgagaaagag agcgagatat aaagggcgct a----ctcag
7990-5 ggcatttt-a atgttagttt ttatgtaggc agctggtatc tctcaggtta gctcacacat tgagaaagag agcgagatat aaagggcgct a----ctcag
7990-3 ggcatttt-a atgttagttt ttatgtaggc agctggtatc tctcaggtta gctcacacat tgagaaagag agcgagatat aaagggcgct a----ctcag
7990-2 ggcattttta atattagttt ttatgtaggc agctggtatc tctcaggtta gctcacacat tgagaaagag agcgagatat aaagggcgct agttactcag
7990-1 ggcattttta atgttagttt ttatgtaggc agctggtagc tctcaggtta gctcacacat tgagaaagag agcgagatat aaagggcgct agttactcag
7990-6 ggcattttta atgttagttt ttatgtaggc agctggtagc tctcaggtta gctcacacat tgagaaagag aacgagatat aaagggcgct agttactcag

       2222222222 2222222222 2222222222 2222222222 2222222222 2222222222 2222222222 2222222222 2222222222 2222222223
       0000000001 1111111112 2222222223 3333333334 4444444445 5555555556 6666666667 7777777778 8888888889 9999999990
       1234567890 1234567890 1234567890 1234567890 1234567890 1234567890 1234567890 1234567890 1234567890 1234567890
7990-4 gagccactaa cgttacaccc agcgggaaga tggcgcttaa acgaatcaat aaggtaagtg ggaaactata tcgagttaga aatatatcag ccagacatat
7990-5 gagccactaa cgttacaccc agcgggaaga tggcgcttaa acgaatcaat aaggtaagtg ggaaactata tcgagttaga aatatatcag ccagacatat
7990-3 gagccactaa cgttacaccc agcgggaaga tggcgcttaa acgaatcaat aaggtaagtg ggaaactata tcgagttaga aatataccag ccagacatat
7990-2 gagccactaa cgttacaccc agcgggaaga tggcgcttaa acgaatcaat aaggtaagtg ggaaactata tcgagttaga aatatatcag ccagacatat
7990-1 gagccactaa cattacaccc agcgggaaga tggcgcttaa acgaatcaat aaggtaagtg ggaaactata tcgagttaga aatatatcag ccagacatat
7990-6 gagccactaa cgttacaccc agcgggaaga tggcgcttaa acgaatcaat aagataagtg ggaaactata tcgagttaga aatatatcag ccagacatat

       3333333333 3333333333 3333333333 3333333333 3333333333 3333333333 3333333333 3333333333 3333333333 3333333334
       0000000001 1111111112 2222222223 3333333334 4444444445 5555555556 6666666667 7777777778 8888888889 9999999990
       1234567890 1234567890 1234567890 1234567890 1234567890 1234567890 1234567890 1234567890 1234567890 1234567890
7990-4 tataaaccag tgtatcttta aatatcttat tcgtccgtca gcgtgtcggc gtagtgctta tgtatgctta ttcctgatac aggcgacgaa tgattttttt
7990-5 tataaaccag tgtatcttta aatatcttat tcgtctgtca gcgtgtcggc gtagtgctta tgtatgctta ttcctgatac aggcgacgaa tgattttt-
7990-3 tataaaccag tgtatcttta aatatcttat tcgtctgtca gcgtgtcggc gtagtgctta tgtatgctta ttcstgatac aggcgacgaa tgattttt-
7990-2 tataaaccag tgtatcttta aatatcttat tcgtctgtca gcgtgtcggc gtagtgctta tgtatgctta ttcctgatac aggcgacgaa tgattttt-
7990-1 tataaaccag tgtatcttta aatatcttat tcgtctgtca gcgtggcggc gtagtgctta tgtatgctta ttcctgatac aggcgacgaa tgattttt-
7990-6 tataaaccag tgtatcatta aatatcttat tcgtctgtca gcgtgtcggc gtagtgctta tgtatgctta ttcctgatac aggcgacgaa tgattttt-

       4444444444 4444444444 4444444444 4444444444 4444444444 4444444444 4444444444 4444444444 4444444444 4444444445
```

```
        0000000001 1111111112 2222222223 3333333334 4444444445 5555555556 6666666667 7777777778 8888888889 9999999990
        1234567890 1234567890 1234567890 1234567890 1234567890 1234567890 1234567890 1234567890 1234567890 1234567890
7990-4  gtttttattt ataaggacat caaaccagtc gttttttaa tattattatt att---tccc attagtctgt agtaatatac aattttaacg cgccgttgtc
7990-5  gtttttattt ataaggacat caaaccagtc gttttttaa tattattatt attattttcc attagtctgt agtaatatac aactttaacg cgccgttgtc
7990-3  gtttttattt ataaggacat caaaccagtc gttttttaa tattattatt att---ttcc attagtctgt agtaatatac aattttaacg cgccgttgtc
7990-2  gtttttattt ataaggacat caaaccagtc gttttttaa tattattatt att---ttcc attagtctgt agtaatatac aattttaacg cgccgttgtc
7990-1  gttttgttt ataaggacat caaacctgtc gttttttaa tattattatt att---ttcc attagtctgt agtaatatac aattttaacg cgccgttgtc
7990-6  gtttttattt ataaggacat caaaccagtc gttttttaa tattattatt att---ttcc attagtctgt agtaatatac aattttaacg cgccgttgtc

        5555555555 5555555555 5555555555 5555555555 5555555555 5555555555 5555555555 5555555555 5555555555 5555555556
        0000000001 1111111112 2222222223 3333333334 4444444445 5555555556 6666666667 7777777778 8888888889 9999999990
        1234567890 1234567890 1234567890 1234567890 1234567890 1234567890 1234567890 1234567890 1234567890 1234567890
7990-4  aaccagtgtg aataatggtt ttgacagcta acttaataat tatttacgta acaccgtctt gatgtcatag gtggcagtgt gaaatgttta gataatacac
7990-5  aaccagtgtg aataatggtt ttgacagcta acttaataat t---tacgta acaccgtctc gatgtaatag gtggcactgt gaaatgttta gataatacat
7990-3  aaccagtgtg aataatggtt ttgacagcta acttaataat tatttacgta acaccgtctc gatgtcatag gtggcagtgt gaaatgttta gataatacac
7990-2  aaccagcgtg aataatggtt ttgacagcca acttaataat tatttacgta acaccgtctc gatgtcatag gtggcactgt gaaatgttta gataatacac
7990-1  aaccagtgtg aataatggtt ttgacagcta acttaataat tatttacgta acaccgtctc gatgtcatag gtggcactgt gaaatgttta gataatacac
7990-6  aaccagtgtg aataatggtt ttgacagcta acttaataat tatttacgta acaccgtctc gatgtcatag gtggcactgt gaaatgttta gataatacac

        6666666666 6666666666 6666666666 6666666666 6666666666 6666666666 6666666666 6666666666 6666666666 6666666667
        0000000001 1111111112 2222222223 3333333334 4444444445 5555555556 6666666667 7777777778 8888888889 9999999990
        1234567890 1234567890 1234567890 1234567890 1234567890 1234567890 1234567890 1234567890 1234567890 1234567890
7990-4  gataaatctc acatcgatat ttaattcata acaatgatta aaattctcac tgtagcattg gcactttgcc ttgtggtgtg ggcagtaaat tcttaccgtc
7990-5  aataaatctc acatcgatat ttaattcata acaatgatta aaatgctcac tgtagcattg gcactttgcc ttgtggtgtg ggcagtaaat tcttaccgtc
7990-3  gataaatctc acatcgatat ttaattcata acaatgatta aaattctcac tgtagcattg gcactttgcc ttgtggtgtg ggcagtaaat ttttaccgtc
7990-2  gataaatctc acatcgatat ttaattcata acaatgatta aaattctcac tgtagcattg gcactttgcc ttgtggtgtg ggcagtaaat ttttaccgtc
7990-1  gataactctc acatcgatat ttaattcata acaatgatta aaattctcac tgtagcattg gcactttgcc ttgtggtgtg ggcagtaaat ttttaccgtc
7990-6  gataaatctc acatcgatat ttaattcata acaatgatta aaattctcac tgtagcattg gcactttgcc ttgtggtgtg ggcagtaaat ttttaccgtc

        7777777777 7777777777 7777777777 7777777777 7777777777 7777777777 7777777777 7777777777 7777777777 7777777778
```

```
        0000000001 1111111112 2222222223 3333333334 4444444445 5555555556 6666666667 7777777778 8888888889 9999999990
        1234567890 1234567890 1234567890 1234567890 1234567890 1234567890 1234567890 1234567890 1234567890 1234567890
7990-4  ttatgaaata aaacgaaaca aaaacttaaa tcgtctttta attaattatt tttt-taaag ttcgtattaa tgtgataatt cctaaacgtt ttgacgtcga
7990-5  ttatgaaata aaacgaaaca aaaactaaaa tcgtctttta attaattatt tttt-taaag ttcgtattaa tgtgataatt cctaaacgtt ttgacgtcga
7990-3  ttatgaaata aaacgaaaca maaactaaaa tcgtctttta attaattatt tttt-taaag ttcgtattaa tgtgataatt cctaaacgtt ttgacgtcga
7990-2  ttatgaaata aaacgaaaca aaaactaaaa tcgtctttta attaattatt tttt-taaag ttcgtattaa tgtgataatt cctaaacgtt ttgacgtcga
7990-1  ttatgaaata aaacgaaaca aaaactaaaa tcgtcattta attaattatt tttt-taagg ttcgtattaa tgtgataatt cctaaacgtt ttgacgtcga
7990-6  ttatgaaata aaacgaaaca aaaactaaaa tcgtctttta attaattatt ttttctaaag ttcgtattaa tgtgataatt cctaaacgtt ttgacgtcga

        8888888888 8888888888 8888888888 8888888888 8888888888 8888888888 8888888888 8888888888 8888888888 8888888889
        0000000001 1111111112 2222222223 3333333334 4444444445 5555555556 6666666667 7777777778 8888888889 9999999990
        1234567890 1234567890 1234567890 1234567890 1234567890 1234567890 1234567890 1234567890 1234567890 1234567890
7990-4  ggtccctttt tactactact cttttagttg acctttaggg ggctgcaagg ttcactttgg gatcccgtgt atttttgtat ccgtattatg aagtaaactt
7990-5  ggtccctttt tactactact cttttagttg acctttaggg ggctgcaagg ttcactttgg gatcccgtgt atttttgtat ccgtattatg aagtaaactt
7990-3  ggtccctttt tactactact cttttagttg acctttaggg ggctgcaagg ttcactttgg gatcccgtgt atttttgtat ccgtattatg aagtaaactt
7990-2  ggtccctttt tactactact cttttagttg acctttaggg ggctgcaagg ttcactttgg gatcccgtgt atttttgtat ccgtattatg aagtaaactt
7990-1  ggtccctttt tactactact cttttagttg acctttaggg ggctgcaagg ttcactttgg gatcccgtgt atttttgtat ccgtattatg aagtaaactt
7990-6  ggtccctttt tactactact cttttagttg acctttaggg ggctgcaagg ttcactttgg gatcccgtgt atttttgtat ctgtattatg aagtaaactt

                                                                                                                 1
        9999999999 9999999999 9999999999 9999999999 9999999999 9999999999 9999999999 9999999999 9999999999 9999999990
        0000000001 1111111112 2222222223 3333333334 4444444445 5555555556 6666666667 7777777778 8888888889 9999999990
        1234567890 1234567890 1234567890 1234567890 1234567890 1234567890 1234567890 1234567890 1234567890 1234567890
7990-4  gtcagagagt tgcgaagcct gtctgtagtt gtgcaatgga agagctctag attctacccc cctgtcatga aatacccccca ggtgtcttcc cactcaacag
7990-5  gtcagagagt tgcgaagcct gtctgtagtt gtgcaatgga agagctctag attctacccc cctgtcatga aatacccccca ggtgtcttcc cactcaacag
7990-3  gtcagagagt tgcgaagcct gtctgtagtt gtgcaatgga agagctctag attctacccc cctgtcatga aatacccccca ggtgtcttcc cactcaacag
7990-2  gtcagagagt tgcgaagcct gtctgtagtt gtgcaatgga agagctctag attctacccc cctgtcatga aatacccccca ggtgtcttcc cactcaacag
7990-1  gtcagagagt tgcgaagcct gtctgtagtt gtgcaatgga agagctctag attctacccc cctgtcatga aatacccccca ggtgtcttcc cactcaacag
7990-6  ctcagagagt tgcgaagcct gtctgtagtt gtgcaatgga agagctctag attctacccc cctgtcatga aatacccccca ggtgtcttcc cactcaacag
```

```
       1111111111 1111111111 1111111111 1111111111 1111111111 1111111111 1111111111 1111111111 1111111111 1111111111
       0000000000 0000000000 0000000000 0000000000 0000000000 0000000000 0000000000 0000000000 0000000000 0000000001
       0000000001 1111111112 2222222223 3333333334 4444444445 5555555556 6666666667 7777777778 8888888889 9999999990
       1234567890 1234567890 1234567890 1234567890 1234567890 1234567890 1234567890 1234567890 1234567890 1234567890
7990-4 acatgttgaa ataaaaataa acgtagtgct cctaaagtag ggagcgcgga cggacatgcc tgtgggcttc aggttgtgtt ccagctcttc tcccgcatga
7990-5 acatgttgaa ataaaaataa acgtagtgct cctaaagtag ggagcgcgga cggacatgcc tgtgggcttc aggttgtgtt ccagctcttc tcccgcatga
7990-3 acatgttgaa ataaaaataa acgtagtgct cctaaagtag tgagcgcgga cggacatgcc tgtgggcttc aggttgtgtt ccagctcttc tcccgcatga
7990-2 acatgttgaa ataaaaataa acgtagtgct cctaaagtag tgagcgcgga cggacatgcc tgtgggcttc aggttgtgtt ccagctcttc tcccgcatga
7990-1 acatgttgaa ataaaaataa acgtagtgct cctaaagtag tgagcgcgga cggacatgcc tgtgggcttc agtttgtgtt ccagctcttc tcccgcatga
7990-6 acatgttgaa ataaaaataa acgtagtgct cctaaagtag ggagcgcgga ctgacatgcc tgtgggcttc aggttgtgtt ccagctcttc tcccgcatga

       1111111111 1111111111 1111111111 1111111111 1111111111 1111111111 1111111111 1111111111 1111111111 1111111111
       1111111111 1111111111 1111111111 1111111111 1111111111 1111111111 1111111111 1111111111 1111111111 1111111112
       0000000001 1111111112 2222222223 3333333334 4444444445 5555555556 6666666667 7777777778 8888888889 9999999990
       1234567890 1234567890 1234567890 1234567890 1234567890 1234567890 1234567890 1234567890 1234567890 1234567890
7990-4 cccctctgga gctataaata caccgtgctg ctacacgctc aatctgccgg tttcatcttg ctgtcaccat agtgataaaa gagccaagcg ccattattga
7990-5 cccctctgga gctataaata caccgtgctg ctacacgctc aatctgccgg tttcatcttg ctgtcgccat agtgataaaa gagccaagcg ccattattga
7990-3 cccctctgga gctataaata caccgtgctg ctacacgctc aatctgccgg tttcatcttg ctgtcaccat agtgataaaa gagccaagcg ccattattga
7990-2 cccctctgga gctattaata caccgtgctg ctacacgctc aatctgccgg tttcatcttg ctgtcaccat agtgataaaa gagccaagcg ccattattga
7990-1 cccctctgaa gctataaata caccgtgctg ctacacgctc aatctgccgg tttcatcttg ctgtcaccat agtgataaaa gagccaagcg ccattattga
7990-6 cccctctgga gctataaata caccgtgctg ctacacgctc agtctgccgg tttcatcttg ctgtcaccat agtgataaaa gagccaagcg ccattattgc

       1111111111 1111111111 1111111111 1111111111 1111111111 1111111111 1111111111 1111111111 1111111111 1111111111
       2222222222 2222222222 2222222222 2222222222 2222222222 2222222222 2222222222 2222222222 2222222222 2222222223
       0000000001 1111111112 2222222223 3333333334 4444444445 5555555556 6666666667 7777777778 8888888889 9999999990
       1234567890 1234567890 1234567890 1234567890 1234567890 1234567890 1234567890 1234567890 1234567890 1234567890
7990-4 cccttcgtta aggagagtct gtgtctttcc agtagttctg tctgtgtgct tgtgtttcac gaagctcatc ctgactcttc cctgtaggaa c-tgaatgat
7990-5 cccttcgtta aggagagtct gtgtctttcc agtagttctg tctgtgtgct tgtgtttcac gaagctcatc ctgactcttc cctgtaggaa c-tgaatgat
7990-3 cccttcgtta aggagagtct gtgtctttcc agtagttctg tctgtgtgct tgtgtttcac gaagctcatc ctgactcttc cctgtaggaa c-tgaatgat
7990-2 cccttcgtta aggagagtct gtgtctttcc agtagttctg tctgtgtgct tgtgtttcac gaagctcatc ctgactcttc cctgtaggaa c-tgaatgat
```

```
7990-1 cccttcgtta aggagagtct gtgtctttcc agtagttctg tctgtgtgct tgtgtttcat gaagctcatc ctgactcttc cctgtaggaa c-tgaatgat
7990-6 cccttcgtta aggagagtct gtgtctttcc agtagttctg tctgtgtgct tgtgtttcac gaaactcatc ggggctctca cacgtgggaa tatctatgct

       1111111
       3333333
       0000000
       1234567
7990-4 ttggcac
7990-5 ttggcac
7990-3 ttggcac
7990-2 ttggcac
7990-1 ttggcac
7990-6 ctgggac
```

SNP LOCATIONS: 139T/G, 577C/G, 692T/C, 1041T/G

29.

```
                1 1111111112 2222222223 3333333334 4444444445 5555555556 6666666667 7777777778 8888888889 9999999990
       1234567890 1234567890 1234567890 1234567890 1234567890 1234567890 1234567890 1234567890 1234567890 1234567890
7992-0 gaacatcata gtgctgggga tgactgccat ggtgacgatg gagatgaaga ccacagtcac tctatgacac atgtcaggac cttagagacg agagctagaa
7992-2 gaacatcata gtgctgggga tgactgccat ggtgacgatg gagatgaaga ccacagtcac tctatgacac atgtcaggac cttagagacg agagctagaa
7992-3 gaacatcata gtgctgggga tgactgccat ggtgacgatg gagatgaaga ccacagtcac tctatgacac atgtcaggac cttagagacg agagctagaa
7992-4 gaacatcata gtgctgggga tgactgccat ggtgacgatg gagatgaaga ccacagtcac tctatgacac atgtcaggac cttagagacg agagctagaa
7992-1 gaacatcata gtgctgggga tgactgccat ggtgacgatg gagatgaaga ccacagtcac tctatgacac atgtcaggac cttagagacg agaactagaa
7992-5 gaacatcata gtgctgggga tgactgccat ggtgacgatg gagatgaaga ccacagtcac tctatgacac atgtcaggac cttagagacg agagctagaa
7992-6 gaacatcata gtgctgggga tgactgccat ggtgatgatg gaaatgaaga ccacagtcac tctatgacac atgtcaggac cttagagacg agagctagaa

       1111111111 1111111111 1111111111 111111111
```

```
       0000000001 1111111112 2222222223 333333333
       1234567890 1234567890 1234567890 123456789
7992-0 aactggaaga gcaggttcac gacctcacag agagataca
7992-2 aactggaaga gcaggttcac gacctcacag agagataca
7992-3 aactggaaga gcaggttcac gacctcacag agagataca
7992-4 aactggaaga gcaggtccac gacctcacag agagataca
7992-1 aactggaaga gcaggtccac gacctcacag agagataca
7992-5 aactggaaga gcaggtccac gacctcacag agagataca
7992-6 aactggaaga gcaggtccac gacctcacag agagataca
```

SNP LOCATIONS: 117C/T

30.

```
                  1 1111111112 2222222223 3333333334 4444444445 5555555556 6666666667 7777777778 8888888889 9999999990
         1234567890 1234567890 1234567890 1234567890 1234567890 1234567890 1234567890 1234567890 1234567890 1234567890
8000-2   ccattacaaa gatgcccaag gatggtttct tttcgtatcc tttgcggcag atctgaggaa caccttcttc atcttctttc ccatctggtt ccatctgaaa
8000-4   ccattacaaa gatgcccaag gatggtttct tttcgtatcc tttgcggcag atctgaggaa caccttcttc atcttctttc ccatctggtt ccatctgaaa
8000-3   ccattacaaa gatgcccaag gatggtttct tttcgtatcc tttgcggcag atctgaggaa caccttcttc atcttctttc ccatctggtt ccatctgaaa
8000-7   ccattacaaa gatgcccaag gatggtttct tttcgtatcc tttgcggcag atctgaggaa caccttcttc atcttctttc ccatctggtt ccatctgaaa
8000-5   ccattacaaa gatgcccaag gatggtttct tttcgtctcc tttgcggcag atctgaggaa caccttcttc atcttctttc ccatctggtt ccatctgaaa
8000-6   ccattacaaa gatgcccaag gatggtttct tttcgtctcc tttgcggcag atctgaggaa caccttcttc atcttccttc ccatctggtt ccatctgaaa
8000-1   ccattacaaa gatgcccaag gatggtttct tttcgtatcc tttgcggcag atctgaggaa caccttcttc atcttctttc ccatctggtt ccatctgaaa

         1111111111 1111111111 1111111111 1111111111 1111111111 1111111111 1111111111 1111111111 1111111111 1111111112
         0000000001 1111111112 2222222223 3333333334 4444444445 5555555556 6666666667 7777777778 8888888889 9999999990
         1234567890 1234567890 1234567890 1234567890 1234567890 1234567890 1234567890 1234567890 1234567890 1234567890
8000-2   gaatcagtcg ggatcaagct catctgggtg gctgtgatag gagactggct caatctggtc ttcaaatggt aagcaaaagg accaataaag tttttactgt
8000-4   gaatcagtcg ggatcaagct catctgggtg gctgtgatag gagactggct caatctggtc ttcaaatggt aagcaaaagg accaataaag tttttactgt
```

```
8000-3  gaatcagtcg ggatcaagct catctgggtg gctgtgatag gagactggct caatctggtc ttcaaatggt aagcaaaagg accaataaag tttttactgt
8000-7  gaatcagtcg ggatcaagct catctgggtg gctgtgatag gagactggct caatctggtc ttcaaatggt aagcaaaagg accaatagag tttttactgt
8000-5  gaatcagtcg ggatcaagct catctgggtg gctgtgatag gagactggct caatctggtc ttcaaatggt aagcagaagg accaataaag tttttactgt
8000-6  gaatcagtcg ggatcaagct catctgggtg gctgtgatag gagactggct caatctggtc ttcaaatggt aagcagaagg accaataaag tttttactgt
8000-1  gaatcagtcg ggatcaagct catctgggtg gctgtgatag gagactggct caatctggtc ttcaaatggt aagcaaaagg accaataaag tttttactgt

        2222222222 2222222222 2222222222 2222222222 2222222222 2222222222 2222222222 2222222222 2222222222 2222222223
        0000000001 1111111112 2222222223 3333333334 4444444445 5555555556 6666666667 7777777778 8888888889 9999999990
        1234567890 1234567890 1234567890 1234567890 1234567890 1234567890 1234567890 1234567890 1234567890 1234567890
8000-2  caaatgaaat catgaattga attttaatgt tgttctattc atctatttaa ggatcctgtt tggagaacgt ccatactggt gg-tccatga gacttattac
8000-4  caaatgaaat catgaattga attttaatgt tgttctattc atctatttaa ggatcctgtt tggagaacgt ccatactggt gggtccatga gacttcttac
8000-3  caaatgaaat catgaattga attttaatgt tgttctattc atctatttaa ggatcctgtt tggagaacgt ccatactggt gggtccatga gacttcttac
8000-7  caaatgaaat catgaattga attttaatgt tgctctattc atctatttaa ggatcctatt tggagaacgt ccatactggt gggtccatga gacttcttac
8000-5  caa-tgaaat catgaattga attttaatgt tgttctattc atctatttaa ggatcctgtt tggagaacgt ccatactggt gggtccatga gacttcttac
8000-6  caaatgaaat catgaattga attttaatgt tgttctattc atctatttaa ggatcctgtt tggaggacgt ccatactggt gggtccatga gacttcttac
8000-1  caaatgaaat catgaattga attttaatgt tgttctattc atctatttaa ggatcctgtt tggagaacgt ccatactggt gggtccatga gacttcttac

        3333333333 3333333333 3333333333 3333333333 3333333333 3333333333 3333333333 3333333333 3333333333 3333333334
        0000000001 1111111112 2222222223 3333333334 4444444445 5555555556 6666666667 7777777778 8888888889 9999999990
        1234567890 1234567890 1234567890 1234567890 1234567890 1234567890 1234567890 1234567890 1234567890 1234567890
8000-2  tacatcaaca gctcaacgcc acatattgag cagtatccca tgacctgtga gaccggccca ggtgcgtctt tctcctctaa agttccatac taatccattt
8000-4  tacatcaaca gctcaacgcc acatattgag cagtatccca tgacctgtga gaccggccca ggtgcgtctt tctcctctaa agttccatac taatccattt
8000-3  tacatcaaca gctcaacgcc acatattggg cagtatccca tgacctgtga gaccggccca ggtgcgtctt tctcctctaa agttccatac taatccattt
8000-7  tacatcaaca gctcaacgcc acatattgag cagtatccca tgacctgtga gaccggccca ggtgcgtctt tctcctctaa agttccatac taatccattt
8000-5  tacatcaaca gctcaacgcc acatattgag cagtatccca tgacctgtga gaccggccca ggtgcgtctt tctcctctaa agttccatac taatccactt
8000-6  tacatcaaca gctcaacgcc acatattgag cagtatccca tgacctgtga gaccggccca ggtgcgtctt tctcctctaa agttccatac taatccactt
8000-1  tacatcaaca gctcaacgcc acatattgag cagtatccca tgacctgtga gaccggccca ggtgcgtctt tctcctctaa agttccatac taatccactt

        4444444444 4444444444 4444444444 4444444444 4444444444 4444444444 4444444444 4444444444 4444444444 4444444445
        0000000001 1111111112 2222222223 3333333334 4444444445 5555555556 6666666667 7777777778 8888888889 9999999990
```

	1234567890	1234567890	1234567890	1234567890	1234567890	1234567890	1234567890	1234567890	1234567890	1234567890
8000−2	atcacctgtt	ccatcataca	cccaaagagc	actttgatca	cttagctgct	tcttgatgca	ggaagcccat	ctggtcatgc	tatgggggct	gctggtgttt
8000−4	atcacctgtt	ccatcataca	cccaaagagc	actttgatca	cttagctgct	tcttgatgca	ggaagcccat	ctggtcatgc	tatgggggct	gctggtgttt
8000−3	atcacctgtt	ccatcataca	cccaaagagc	actttgatca	cttagctgct	tcttgatgca	ggaagcccat	ctggtcatgc	tatgggggct	gctggtgttt
8000−7	atcacctgtt	ccatcataca	cccaaagagc	actttgatca	cttagctgct	tcttgatgca	ggaagcccat	ctggtcatgc	tatgggggct	gctggtgttt
8000−5	atcacctgtt	ccatcataca	cccaaagagc	actttgatca	cttagctgct	tcttgatgca	ggaagcccat	ctggtcatgc	tatgggggct	gctggtgttt
8000−6	atcacctgtt	ccatcataca	cccaaagagc	actttgatca	cttagctgct	tcttgatgca	ggaagcccat	ctggtcatgc	tatgggggct	gctggtgttt
8000−1	atcacctgtt	ccatcataca	cccaaagagc	actttgatca	cttagctgct	tcttgatgca	ggaagcccat	ctggtcatgc	tatgggggct	gctggtgttt

	5555555555	5555555555	5555555555	5555555555	5555555555	5555555555	5555555555	5555555555	5555555555	5555555556
	0000000001	1111111112	2222222223	3333333334	4444444445	5555555556	6666666667	7777777778	8888888889	9999999990
	1234567890	1234567890	1234567890	1234567890	1234567890	1234567890	1234567890	1234567890	1234567890	1234567890
8000−2	actacacatt	ggtcacttca	atccttgcca	taatgctaag	caaagagaag	aaaacctcgt	ctaagggcct	gtaagtcaat	ctttatcaca	attagactgt
8000−4	actacacatt	ggtcacttca	atccttgcca	taatgctaag	caaagagaag	aaaacctcgt	ctaagggcct	gtaagtcaat	ctttatcaca	attagactgt
8000−3	actacacatt	ggtcacttca	atccttgcca	taatgctaag	caaagagaag	aaaacctcgt	ctaagggcct	gtaagtcaat	ctttatcaca	attagactgt
8000−7	actacacatt	ggtcacttca	atccttgcca	taatgctaag	caaagagaag	aaaacctcgt	ctaagggcct	gtaagtcaat	ctttatcaca	attagactgt
8000−5	actacacatt	ggtcacttca	atccttgcca	taatgctgag	caaagagaag	aaaacctcgt	ctaagggcct	gtaagtcaat	ctttatcaca	attagactgt
8000−6	actacacatt	ggtcacttca	atccttgcca	taatgctgag	caaagagaag	aaaacctcgt	ctaagggcct	gtaagtcaat	ctttatcaca	attagactgt
8000−1	actacacatt	ggtcacttca	atccttgcca	taatgctgag	caaagagaag	aaaacctcgt	ctaagggcct	gtaagtcaat	ctttatcaca	attagactgt

	6666666666	6666666666	6666666666	6666666666	6666666666	6666666666	6666666666	6666666666	6666666666	6666666667
	0000000001	1111111112	2222222223	3333333334	4444444445	5555555556	6666666667	7777777778	8888888889	9999999990
	1234567890	1234567890	1234567890	1234567890	1234567890	1234567890	1234567890	1234567890	1234567890	1234567890
8000−2	tttcacaccg	cttctgtttc	aaaatagata	gttgcctctg	cagtatgttt	atgatgtact	atctgttttt	taatcagata	cttgcgtggt	tcactttgga
8000−4	tttcacaccg	cttctgtttc	aaaatagata	gttgcctctg	cagtatgttt	atgatgtact	atctgttttt	taatcagata	cttgcgtggt	tcactttgga
8000−3	tttcacaccg	cttctgtttc	aaaatagata	gttgcctctg	cagtatgttt	atgatgtact	atctgttttt	taatcagata	cttgcgtggt	tcactttgga
8000−7	tttcacaccg	cttctgtttc	aaaatagata	gttgcctctg	cagtatgttt	atgatgtact	atctgttttt	taatcagata	cttgcgtggt	tcactttgga
8000−5	tttcacaccg	cttctgtttc	aaaatagata	gttgcctctg	caatatgttt	atgatgtact	atctgttttt	taatcagata	cttgcgtggt	tcactttgga
8000−6	tttcacaccg	cttctgtttc	aaaatagata	gttgcctctg	caatatgttt	atgatgtact	atctgttttt	taatcagata	cttgcgtggt	tcactttgga
8000−1	tttcacaccg	cttctgtttc	aaaatagata	gttgcctctg	caatatgttt	atgatgtact	atctgttttt	taatcagata	cttgcgtggt	tcactttgga

```
          777777777
          000000000
          123456789
8000-2    cgctcttct
8000-4    cgctcttct
8000-3    cgctcttct
8000-7    cgctcttct
8000-5    cgctcttct
8000-6    cgctcttct
8000-1    cgctcttct
```

SNP LOCATIONS: 37A/C, 176A/G, 398T/C, 538A/G, 643G/A

31.

```
                 1 1111111112 2222222223 3333333334 4444444445 5555555556 6666666667 7777777778 8888888889 9999999990
        1234567890 1234567890 1234567890 1234567890 1234567890 1234567890 1234567890 1234567890 1234567890 1234567890
8001-2  accatcagtg ccgccctctg ctatgctaca cagctcatca atatcctgtc tcatattctt gatgtcagcc ttccgaaaaa attgtgcaac aggttacagc
8001-4  accatcagtg ccgccctctg ctatgctaca cagctcatca atatcctgtc tcatattctt gatgtcagcc ttccgaaaaa attgtgcaac aggttacagc
8001-5  accatcagtg ccgccctctg ctatgctaca cagctcatca atatcctgtc tcatattctt gatgtcagcc ttccgaaaaa attgtgcaac aggttacagc
8001-1  accatcagtg ccgccctctg ctatgctaca cagctcatca atatcctgtc tcatattctt gatgtcaacc ttccgaaaaa attgtgcaac aggttacagc
8001-6  accatcagtg ccgccctctg ctatgctaca cagctcatca atatcctgtc tcatattctt gatgtcaacc ttccgaaaaa attgtgcaac aggttacagc
8001-3  accatcagtg ccgccctctg ctatgctaca cagctcatca atatcctgtc tcatattctt gatgtcaacc ttccgaaaaa attgtgcaac aggttacagc

        1111111111 1111111111 1111111111 1111111111 1111111111 1111111111 1111111111 1111111111 1111111111 1111111112
        0000000001 1111111112 2222222223 3333333334 4444444445 5555555556 6666666667 7777777778 8888888889 9999999990
        1234567890 1234567890 1234567890 1234567890 1234567890 1234567890 1234567890 1234567890 1234567890 1234567890
8001-2  tttatgttaa tggtctatta tagtttttat tcacattttg aaaaattttt ttgtttctat ttttgtttta attttactta acgtttcagt aattttgttg
8001-4  tttatgttaa tggtctatta tagtttttat tcacattttg aaaaattttt ttgtttctat ttttgtttta attttactta acgtttcagt aattttgttg
8001-5  tttatgttaa tggtctatta tagtttttat tcacattttg aaaaattttt ttgtttctat ttttgtttta attttactta acgtttcagt aattttgttg
```

```
8001-1 tttatgttaa tggtctatta tagtttttat tcatactttg aaattttttt ttgtttctat ttttatttta attttactta acgtttcagt aattttgttg
8001-6 tttatgttaa tggtctatta tagtttttat tcacattttg aaattttttt ttgtttctat ttttgtttta attttactta acgtttcagt aattttgttg
8001-3 tttatgttaa tggtctatta tagtttttat tcacattttg aaattttttt ttgtttctat ttttgtttta attctactta acgtttcagt aattttgttg

       2222222222 2222222222 2222222222 2222222222 2222222222 2222222222 2222222222 2222222222 2222222222 2222222223
       0000000001 1111111112 2222222223 3333333334 4444444445 5555555556 6666666667 7777777778 8888888889 9999999990
       1234567890 1234567890 1234567890 1234567890 1234567890 1234567890 1234567890 1234567890 1234567890 1234567890
8001-2 tttttgacac tttttctggt ttttaaaatg tctatatagt ttcatttgtt tattttttt-a cttcataaag ttactataaa tggaaaataa taaatgttgc
8001-4 tttttgacac tttttctggt ttttaaaatg tctatatagt ttcatttatt tattttttt-a cttcataaag ttactataaa tggaaaataa taaatgttgc
8001-5 tttttgacac tttttctggt ttttaaaatg tctatatagt ttcatttatt tattttttt-a cttcataaag ttactataaa tggaaaataa taaatgttgc
8001-1 tttttgacac tttttctggt ttttaaaatg tctatatagt tttatttatt tattttttt-a cttcataaag ttactataaa tggaaaataa taaatgttgc
8001-6 tttttgacac tttttctggt ttttaaaatg tctatatagt ttcatttatt tatttttttta cttcataaag ttactataaa tggaaaataa taaatgttgc
8001-3 ttttttacac tttttctggt ttttaaaatg tctatatagt ttcatttatt tattttttt-a cttcataaag ttactataaa aagaaaataa taaatgttgc

       3333333333 3333333333 3333333333 3333333333 3333333333 3333333333 3333333333 3333333333 3333333333 3333333334
       0000000001 1111111112 2222222223 3333333334 4444444445 5555555556 6666666667 7777777778 8888888889 9999999990
       1234567890 1234567890 1234567890 1234567890 1234567890 1234567890 1234567890 1234567890 1234567890 1234567890
8001-2 cctgacaact agctgaaaca agtttttttt t--tttgtat tttattataa tctaaaaatg aatatcctgt attctgtttt gttttctaca gtgagttctg
8001-4 cctgacaact agctgaaaca agtttttttt tcttttgtat tttattataa tctaaaaatg aatgtcctgt attctgtttt gttttctaca gtgagttctg
8001-5 cctgacaact agctgaaaca agtttttttt t-ttttgtat tttattataa tctaaaaatg aatgtcctgt attctgtttt gttttctaca gtgagttctg
8001-1 cctgacaact agctgaaaca agtttttttt ttttttgtat tttattataa tctaaaaatg aatgtcctgt attctgtttt gatttctaca gtgagttctg
8001-6 cctgacaact agctgaaaca agtttttttt t--tttgtat tttattataa tctaaaaatg aatgtcctgt attctgtttt gttttctaca gtgagttctg
8001-3 cctgacaact agctgaaaca agtttttttt t-ttttgtat tttattataa tctcaaaatg aatgtcctgt attctgtttt gttttctaca gtgagttctg

       4444444444 4444444444 4444444444 4444444444 4444444444 4444444444 4444444444 4444444444 4444444444 4444444445
       0000000001 1111111112 2222222223 3333333334 4444444445 5555555556 6666666667 7777777778 8888888889 9999999990
       1234567890 1234567890 1234567890 1234567890 1234567890 1234567890 1234567890 1234567890 1234567890 1234567890
8001-2 tggtgataat ctgacccgct accgattcac ccgtgccgta aacaaactta acaccaacat cctgcacctc tgcttctcgc aggtaaattc ttctcaaaac
8001-4 tggtgataat ctgacccgct accgattcac ccgtgccgta aacaaactta acaccaacat cctgcacctc tgcttctcgc aggtaaattc ttctcaaaac
8001-5 tggtgataat ctgacccgct accgattcac ccgtgccgta aacaaactta acaccaacat cctgcacctc tgcttctcgc aggtaaattc ttctcaaaac
```

```
8001-1 tggtgataat ctgacccgct accgattcac ccgtgccgta aacaaactta acaccaacat cctgcacctc tgcttctcgc aggtaaattc ctctcaaaac
8001-6 cggtgataat ctgacccgct accgattcac ccgtgccgta aacaaactta acaccaacat cctgcacctc tgcttctcgc aggtaaattc ttctcaaaac
8001-3 cggtgataat ctgacccgct accgattcac ccgtgctgta aacaaactta acaccaacat cctgcacctc tgcttctcac aggtaaattc ttcccaaaac

       5555555555 5555555555 5555555555 5555555555 5555555555 5555555555 5555555555 5555555555 5555555555 5555555556
       0000000001 1111111112 2222222223 3333333334 4444444445 5555555556 6666666667 7777777778 8888888889 9999999990
       1234567890 1234567890 1234567890 1234567890 1234567890 1234567890 1234567890 1234567890 1234567890 1234567890
8001-2 cgagaccact ctgatctgag aagtcttatt ctgatctcat gcctcatgtt tatgcttgat tcttagcatg ttgacagcga gctgcttcat cctcatcaca
8001-4 tgagaccact ctgatctgag aagtcttatt ctgatctcat gcctcatgtt tatgcttgat tcttagcatg ttgacagcga gctgctccat cctcatcaca
8001-5 tgagaccact ctgatctgag aagtcttatt ctgatctcat gcctcatgtt tatgcttgat tcttagcatg ttgacagcga gctgcttcat cctcatcaca
8001-1 tgagaccact ctgatctgag aagtcttatt ctgatctcat gcctcatgtt tatgcttgat tcttagcatg ttgacagcga gctgcttcat cctcatcaca
8001-6 tgagaccact ctgatctgag aagtcttatt ctgatctcat gcctcatgtt tatgctggat tcttagcatg ttgacagcga gctgcttcat cctcatcaca
8001-3 tgagaccact ctgatctgag aagtcttatt ctgatctcat gcctcatgtt tatgcttgat tcttagcatg ttgacagcga gctgcttcat cctcatcaca

       6666666666 6666666666 6666666666 6666666666 6666666666 6666666666 6666666666 6666666666 6666666666 6666666667
       0000000001 1111111112 2222222223 3333333334 4444444445 5555555556 6666666667 7777777778 8888888889 9999999990
       1234567890 1234567890 1234567890 1234567890 1234567890 1234567890 1234567890 1234567890 1234567890 1234567890
8001-2 ctttgaggaa tataatattt ctggtgtctc cagcaaacaa gaaacttggc aggtgagaaa ttacattgtc ctgttcttta gtgaaacaat ttacttttat
8001-4 ctttgaggaa tataatattt ctggtgtctc cagcaaacaa gaaacttggc aggtgagaaa ttacattgtc ctgttcttta gtgaaacaat ttacttttat
8001-5 ctttgaggaa tataatattt ctggtgtctc cagcaaacaa gaaacttggc aggtgagaaa ttacattgtc ctgttcttta gtgaaacaat ttacttttat
8001-1 ctttgaggaa tataatattt ctggtgtctc cagcaaacaa gaaacttggc aggtgagaaa ttacattgtc ctgttcttta gtgaaacaat ttacttttat
8001-6 ctttgaggaa tataatattt ctggtgtctc cagcaaacaa gaaacttggc aggtgagaaa ttacattgtc ctgttcttta gtgaaacaat ttacttttat
8001-3 ctttgaggaa tataatattt ctggtgtctc cagcaaacaa gaaacttggc aggtgagaaa ttacattgtc ctgttcttta gtgaaacaat ttacttttat

       7777777777 7777777777 7777777777 7777777777 7777777777 7777777777 7777777777 7777777777 7777777777 7777777778
       0000000001 1111111112 2222222223 3333333334 4444444445 5555555556 6666666667 7777777778 8888888889 9999999990
       1234567890 1234567890 1234567890 1234567890 1234567890 1234567890 1234567890 1234567890 1234567890 1234567890
8001-2 gttgtccaca atgtat---- ---------- -ttatgatcc aaataaattc caatagataa aatcaagtg- -gtcttgatt atttgctgat gatttttatt
8001-4 gttgtccaca atgtat---- ---------- -ttatgatcc aaataaattc -aatagataa aatcaagtg- -gtcttaatt atttgctgat gatttttatt
8001-5 gttgtccaca atgtat---- ---------- -ttatgatcc aaataaattc caatagataa aatcaagtg- -gtcttaatt atttgctgat gatttttatt
```

```
8001-1 gttgtccaca atgtat---- ---------- -ttatgatcc aaataaattc cagtagataa aatcaagtg- -gtcttaatt atttgctgat gatttttatt
8001-6 gttgtccacg atgtatgtta tagcccatta tttatgatcc aaataaattc cagttgataa aatcaagtgt tgtcttaatt atttgctgat gatttttttt
8001-3 gttgtccaca atgtatgtta tagcccatta tttatgatcc aaataaattc caatagataa aatcaagtgt tgtcttaatt atttgctgat gatttttttt

       8888888888 8888888888 8888888888 8888888888 8888888888 8888888888 8888888888 8888888888 8888888888 8888888889
       0000000001 1111111112 2222222223 3333333334 4444444445 5555555556 6666666667 7777777778 8888888889 9999999990
       1234567890 1234567890 1234567890 1234567890 1234567890 1234567890 1234567890 1234567890 1234567890 1234567890
8001-2 tttttttcac tccctgtata attttttttt ttccactact gttcaaaaac tttggtgtca ttagatagga cataattaaa ttgatcaaaa gtgacagtaa
8001-4 tttttt-cac tccctgtata attttttttt t-ccactact gttcaaaaac tttggtgtca ttagatagga cataattaaa ttgatcaaaa gtgacagtaa
8001-5 tttttt-cac tccctgtata attttttttt t-ccactact gttcaaaaac tttggtgtca ttagatagga cataattaaa ttgatcaaaa gtgacagtaa
8001-1 tttttt-cac tccctgtata attttttttt c--cactact gttcaaaaac tttggtgtca ttagatagga cataattaaa ttgatcaaaa gtgacagtaa
8001-6 tttttc--ac tccctgtata attttttttt c--cactact gttcaaaaac tttggtgtca ttagatagga cataattaaa ttgatcaaaa gtgacagtaa
8001-3 ttttc---ac tccctgtata attttttttt c--cactact gttcaaaaac tttggtgtca ttagatagga cataattaaa ttgatcaaaa gtgacagtaa

                                                                                                                1
       9999999999 9999999999 9999999999 9999999999 9999999999 9999999999 9999999999 9999999999 9999999999 9999999990
       0000000001 1111111112 2222222223 3333333334 4444444445 5555555556 6666666667 7777777778 8888888889 9999999990
       1234567890 1234567890 1234567890 1234567890 1234567890 1234567890 1234567890 1234567890 1234567890 1234567890
8001-2 agacttataa tctaattcaa ataaattctg ttcttttgaa ctttatattc atcaaagaat cctgaaaaaa aaaatgtaac atggktttta catcaataat
8001-4 agacttataa tctaattcaa ataaattctg ttcttttgaa ctttatattc atcaaagaat cctgaaaaaa aaaatgtaac atggttttta catcaataat
8001-5 agacttataa tctaattcaa ataaattctg ttcttttgaa ctttatattc atcaaagaat cctgaaaaaa aaaatgtaac atggttttta catcaataat
8001-1 agacttataa tctaactcaa ataaattctg ttcttttgaa ctttatattc atcaaagaat cctgaaaaaa aaaatgtaac atggttttta catcaataat
8001-6 agacttataa tctaattcaa ataaattctg ttcttttgaa ctttatattc atcaaagaat cctgaaaaaa aaaatgtaac atggttttta catcaataat
8001-3 agacttataa tctaattcaa ataaattctg ttcttttgaa ctttatattc atcaaagaat cctgaaaaaa aaa-tgtaac atggttttta catcaataat

       1111111111 1111111111 1111111111 1111111111 1111111111 1111111111 1111111111 1111111111 1111111111 1111111111
       0000000000 0000000000 0000000000 0000000000 0000000000 0000000000 0000000000 0000000000 0000000000 0000000001
       0000000001 1111111112 2222222223 3333333334 4444444445 5555555556 6666666667 7777777778 8888888889 9999999990
       1234567890 1234567890 1234567890 1234567890 1234567890 1234567890 1234567890 1234567890 1234567890 1234567890
8001-2 attaaatgtt tcttgagcag caaatcagca tattagaatg atttctgaag gatcatgtga cactaaagac tgtagtaatg atgccgaaaa ttcagctttg
```

```
8001-4 attaaatgtt tcttgagcag caaatcagca tattagaatg atttctgaag gatcatgtga cactaaagac tgtagtaatg atgccgaaaa ttcagctttg
8001-5 attaaatgtt tcttgagcag caaatcagca tattagaatg atttctgaag gatcatgtga cactaaagac tgtagtaatg atgccgaaaa ttcagctttg
8001-1 attaaatgtt tcttgagcag caaatcagcg tattagaatg atttctgaag gatcatgtga cactaaagac tgtagtaatg atgccgaaaa ttcagctttg
8001-6 actaaatgtt tcttgagcag caaatcagca tattagaatg atttctgaag gatcatgtga cactaaagac tgtagtaatg atgccgaaaa ttcagctttg
8001-3 attaaatgtt tcttgagcag caaatcagca tattagaatg atttctgaag gatcatgtga cactaaagac tgtagtaatg atgccgaaaa ttcagctttg

       1111111111 1111111111 1111111111 1111111111 1111111111 1111111111 1111111111 1111111111 1111111111 1111111111
       1111111111 1111111111 1111111111 1111111111 1111111111 1111111111 1111111111 1111111111 1111111111 1111111112
       0000000001 1111111112 2222222223 3333333334 4444444445 5555555556 6666666667 7777777778 8888888889 9999999990
       1234567890 1234567890 1234567890 1234567890 1234567890 1234567890 1234567890 1234567890 1234567890 1234567890
8001-2 catcacagga ctatatttta aaatatattt aaatgtaaat gttgttttaa attgtagtaa taattcataa atattattga ttttacaaat aaatgcagcc
8001-4 catcacagga ctatatttta aagtatattt aaatgtaaat gttgttttaa attgtagtaa taattcataa atattattga ttttacaaat aaatgcagcc
8001-5 catcacagga ctatatttta aaatatattt aaatgtaaat gttgttttaa attgtagtaa taattcataa atattattga ttttacaaat aaatgcagcc
8001-1 catcacagga ctatatttta aaatatattt aaatgtaaat gttgttttaa attgtagtaa taattcataa atattattga ttttacaaat aaatgcagcc
8001-6 catcacagga ctatatttta aaatatattt aaatgtaaat tttgttttaa attgtagtaa taattcataa atattattga ttttacaaat aaatgcagcc
8001-3 catctcagga ctatatttta aaatatattt aaatgtaaat gttgttttaa attgtagtaa taattcataa aaattattga ttttacaaat aaatgcagcc

       1111111111 1111111111 1111111111 1111111111 1111111111 1111111111 1111111111 1111111111 1111111111 1111111111
       2222222222 2222222222 2222222222 2222222222 2222222222 2222222222 2222222222 2222222222 2222222222 2222222223
       0000000001 1111111112 2222222223 3333333334 4444444445 5555555556 6666666667 7777777778 8888888889 9999999990
       1234567890 1234567890 1234567890 1234567890 1234567890 1234567890 1234567890 1234567890 1234567890 1234567890
8001-2 ttgctgaaca tacgagaaaa aaaagcatta aaaaaaaaac ttgccgaccc caaacctttg aatgctagta taagtcatat aatggataac ttatgtgttg
8001-4 ttgctgaaca tacgagaaaa aaa-gcattt aaaaaaaaac ttgccgaccc caaacctttg aatgctagta taagtcatat aatggataac ttatgtgttg
8001-5 ttgctgaaca tacgagaaaa aaagcattta aaaaaaaaac ttgccgaccc caaacctttg aatgctagta taagtcatat aatggataac ttatgtgttg
8001-1 ttgctgaaca tacgagaaaa aaagcattta aaaaaaaaac ttgccgaccc caaacctttg aatgctagta taagtcatat aatggataac ttatgtgttg
8001-6 ttgctgaaca tacgagaaaa aaa-gcatta aaaaaaaaac ttgccgaccc caaacctttg aatgctagta taagtcatat aatggataac ttatgtgttg
8001-3 ttgctgaaca tatgagaaaa aag-cattaa aaaaagaaac ttgccgaccc caaacctttg aatgctagta taagtcatat aatggataac ttatgtgttg

       1111111111 1111111111 1111111111 1111111111 1111111111 1111111111 1111111111 1111111111 1111111111 1111111111
       3333333333 3333333333 3333333333 3333333333 3333333333 3333333333 3333333333 3333333333 3333333333 3333333334
```

```
        0000000001 1111111112 2222222223 3333333334 4444444445 5555555556 6666666667 7777777778 8888888889 9999999990
        1234567890 1234567890 1234567890 1234567890 1234567890 1234567890 1234567890 1234567890 1234567890 1234567890
8001-2 caagcatttc cactcttgtt cactagaacc gggccgtttg aggtgagcgc tgacttggaa gactctatgg agtttgtgga accagaggcg gctggaccag
8001-4 caaacatttc cactcttgtt cactagaacc gggccgtttg aggtgagcgc tgacttggag gactctatgg agtttgtgga accagaggcg gctggaccag
8001-5 caaacatttc cactcttgtt cactagaacc gggccgtttg aggtgagcgc tgacttggaa gactctatgg agtttgtgga accagaggcg gctggaccag
8001-1 caaacatttc cactcttgtt cactagaacc gggccgtttg aggtgagcgc tgacttggaa gactctatgg agtttgtgga accagaggcg gctggaccag
8001-6 caaacatttc cactcttgtt cactagaacc gggccgtttg aggtgagcgc tgacttggaa gactctatgg agtttgtgga accagaggcg gctggaccag
8001-3 caaacatttc cactcttgtt cactagaacc gggccgtttg aggtgagcgc tgacttggaa gaatctatgg agtttgtgga accagaggcg gctggaccag

        1111111111 1111111111 1111111111 111
        4444444444 4444444444 4444444444 444
        0000000001 1111111112 2222222223 333
        1234567890 1234567890 1234567890 123
8001-2 cggaggagag cggagaggag gctgtgacgg atg
8001-4 cggaggagag cggagaggag gctgtgacgg atg
8001-5 cggaggagag cggagaggag gctgtgacgg atg
8001-1 cggaggagag cggagaggag gctgtgacgg atg
8001-6 cggaggagag cggagaggag gctgtgacgg atg
8001-3 cggaggagag cggagaggag gctgtgacgg atg
```

SNP LOCATIONS: 68G/A, 144A/T, 145A/T, 400T/C, 753A/G, 798A/T, 831T/C

32.

```
                 1 1111111112 2222222223 3333333334 4444444445 5555555556 6666666667 7777777778 8888888889 9999999990
        1234567890 1234567890 1234567890 1234567890 1234567890 1234567890 1234567890 1234567890 1234567890 1234567890
8008-2 gctgagccaa gtgggagata tcagcagctg cccagtgaag aggagccgga ggagggagct caggtggcca gtgaccctcc gcccccttac agtagtattg
8008-3 gctgagccaa gtgggagata tcagc---tg cccagtgaag aggagccgga ggagggagct caggtggcca gtgaccctcc gcccccttac agtagtattg
8008-4 gctgagccaa gtgggagata tcagc---tg cccagtgaag aggagccgga ggagggagct caggtggcca gtgaccctcc gcccccttac agtagcattg
8008-1 gctgagccaa gtgggagata tcagcagctg cccagtgaag aggagccgga ggagggagct caggtggcca gtgaccctcc gcccccttac agtagtattg
```

```
        1111111111 1111111111 1111111111 1111111111 1111111111 1111111111 1111111111 1111111111 1111111111 1111111112
        0000000001 1111111112 2222222223 3333333334 4444444445 5555555556 6666666667 7777777778 8888888889 9999999990
        1234567890 1234567890 1234567890 1234567890 1234567890 1234567890 1234567890 1234567890 1234567890 1234567890
8008-2 cagctgacag cgcagtcttc tcagcatatt ttgattacaa agacgatgca gcatttccca agcccccatc ttacaacgta gccacatctc taccatcgta
8008-3 cagctgacag cgcagtcttc tcagcatatt tcgattacaa agacgatgca gcgtttccca agcccccatc ttacaacgta gccacatctc taccatcgta
8008-4 cagctgacag cgcagtcttc tcaggatatt ttgattacaa agacgatgca gcatttccca agcccccatc ttacaacgta gc-acatctc taccatcgta
8008-1 cagctgacag cggggtcttc tcaagatatt ttgattacaa agacgatgca gcatttccca agcccccatc ttacaacgta gccacatctc taccgtcgta

        2222222222 2222222222 2222222222 222
        0000000001 1111111112 2222222223 333
        1234567890 1234567890 1234567890 123
8008-2 cgatgaggct gagagaaaca aagcagaggc gac
8008-3 cgatgaggct gagagaaaca aagcagaggc gac
8008-4 cgatgaggct gagagaaaca aagcagaggc gac
8008-1 cgatgaggct gagagaaaca aagcagaggc gac
```

SNP LOCATIONS: 125C/G

33.

```
                    1 1111111112 2222222223 3333333334 4444444445 5555555556 6666666667 7777777778 8888888889 9999999990
           1234567890 1234567890 1234567890 1234567890 1234567890 1234567890 1234567890 1234567890 1234567890 1234567890
8009-2-1 aggtgatgtt cctgtctggg tttattgtca gatgatctca gatgggaaag atgaagacaa aggaggatgg atggtaataa tgtgacttca gatcattaaa
8009-2-5 aggtgatgtt cctgtctggg tttattgtca gatgatctca gatggaaaag atgaagacaa aggaggatgg acggtaataa tgtgacttca gatcattaaa
8009-2-2 aggtgatgtt cctgtctggg tttactgtca gatgatctca gatggaaaag atgaagacaa aggaggatgg acggtaataa tgtgacttca gatcattaaa
8009-2-3 aggtgatgtt cctgtctggg tttactgtca gatgatctca gatggaaaag atgaagacaa aggaggatgg acggtaataa tgtgacttca gatcattaaa
8009-2-4 aggtgatgtt cctgtctggg tttactgtca gatgatctca gatggaaaag atgaagacaa aggaggatgg acggtaataa tgtgacttta gatcattaaa
8009-2-6 aggtgatgtt cctgtctggg tttactgtca gatgatctca gatgggaaag atgaagacaa aggaggatgg acggtaggaa tgtgacttta gatcattaaa

         1111111111 1111111111 1111111111 1111111111 1111111111 1111111111 1111111111 1111111111 1111111111 1111111112
         0000000001 1111111112 2222222223 3333333334 4444444445 5555555556 6666666667 7777777778 8888888889 9999999990
```

```
          1234567890 1234567890 1234567890 1234567890 1234567890 1234567890 1234567890 1234567890 1234567890 1234567890
8009-2-1 aacacatcaa aacctttatt ataaatcata tattctatat gaactcctca caggtgattc agaggagaat ggacggcagt gtgaatttct atcggccgtg
8009-2-5 aacacatcaa aacctttatt ataaatcata tattctatat gaactcctca caggtgattc agaggagaat ggacggcagt gtgaatttct atcggccgtg
8009-2-2 aacacatcaa aacctttatt ataaatcata tattctatat gaactcctca caggtgattc agaggagaat ggacggcagt gtgaatttct atcggccgtg
8009-2-3 aacacatcaa aacctttatt ataaatcata tattctatat gaactcctca caggtgattc agaggagaat ggacggcagt gtgaatttct atcggccgtg
8009-2-4 cacacatcaa aacctttatt ataaatcata tattctatgt gaactcctca caggtgattc agaggagaat ggacggcagt gtgaatttct atcggccgtg
8009-2-6 aacacatcac aagattcatt ataaatcata tattctatat gaacccatca caggtgattc agaggagaat ggacggcagt gtgaatttct atcggccatg

          2222222222 2222222222 2222222222 2222222222 2222222222 2222222222 2222222222 2222222222 2222222222 2222222223
          0000000001 1111111112 2222222223 3333333334 4444444445 5555555556 6666666667 7777777778 8888888889 9999999990
          1234567890 1234567890 1234567890 1234567890 1234567890 1234567890 1234567890 1234567890 1234567890 1234567890
8009-2-1 gaatcagtac aggagaggat ttgggaatgt ggagagagaa tactggctgg gtaagatctc acagcgtgtg tgtgtttgtg tgtgtgtgtg tgtgt-----
8009-2-5 gaatcagtac aggagaggat ttgggaatgt ggcgagtgaa tactggctgg gtaagatctc acagtgtgtg tgtgtttgtg tgtgtgtgtg tgtgtgtgtg
8009-2-2 gaatcagtac aggagaggat ttgggaatgt ggagagagaa tactggctgg gtaagatctc acagcgtgtg tgtgtgtgtg tgtgtgtgtg tgtgtgtgtg
8009-2-3 gaatcagtac aggagaggat ttgggaatgt ggagagagaa tactggctgg gtaagatctc acagcgtgtg tgtgtttgtg tgtgtgtgtg tgtgtgtgtg
8009-2-4 gaatcagtac aagagaggat ttgggaatgt ggagagggaa tactggctgg gtaagatctc acagcgtgtg tgtatgtgtg tgtgtgtgtg tgtgtgtgtg
8009-2-6 ggatcagtac aagagaggat ttgggaatgt ggagggagaa tactggctgg gtaagatctt acagtgtgtg tgtgtgtgtg tgtgtgtgtg tgtgtgtgtg

          3333333333 3333333333 3333333333 3333333333 3333333333 3333333333 3333333333 3333333333 3333333333 3333333334
          0000000001 1111111112 2222222223 3333333334 4444444445 5555555556 6666666667 7777777778 8888888889 9999999990
          1234567890 1234567890 1234567890 1234567890 1234567890 1234567890 1234567890 1234567890 1234567890 1234567890
8009-2-1 ---------- -------atg ttcatgtcca gtgtttggtt gaacgtgttt ttcatgtgtg tgtgtatgat cagggctgga gaacatgtac caggtgacac
8009-2-5 tgtgtgtgtg tgg----atg ttcatgtcca gtgtttggtt gaacgtgttc ttcatgtgtg tgtgtatgat cagggctgga gaacatgtac caggtgacac
8009-2-2 tgtgtgtgt- ---------- -tcatgtcca gtgtttggtt gagcgtgttc tccatgtgtg tgtgtatgat cagggctgga gaacatgtac caggtgacac
8009-2-3 tgtgtgtgtg tgtgtggata ttcatgtcca gtgtttggtt gaacgtgttc ttcatgtgtg tgtgtatgat cagggctgga gaacatgtac caggtgacac
8009-2-4 tgtgtgtgtg tgtgtgtatg tttatgtcca gtgtttggtt gaacgtgttc ttcatgtgtg tg--tatgat cagggctgga gaacatgtac caggtgacac
8009-2-6 tg-------- ------gatg ttcatgtcca gtgtttggtt gaatgtgttc ttcatgtgtg tg--tatgat cagggctgga aaacatgtac cagctgacac

          4444444444 444444
          0000000001 111111
```

```
          1234567890 123456
8009-2-1 gcaacaggaa gtacat
8009-2-5 gcaacaggaa gtacat
8009-2-2 gcaacaggaa gtacat
8009-2-3 gcaacaggaa gtacat
8009-2-4 gcaacaggaa gtacat
8009-2-6 gcaacaggaa gtacat
```

SNP LOCATIONS: 25C/T, 45A/G, 89C/T, 212G/A, 265C/T, 276G/T

34.

```
                 1 1111111112 2222222223 3333333334 4444444445 5555555556 6666666667 7777777778 8888888889 9999999990
        1234567890 1234567890 1234567890 1234567890 1234567890 1234567890 1234567890 1234567890 1234567890 1234567890
8011-1 tggtttggag ggcggttagc ttttgctaca caacactggc aaagttttac ctcgttatcg ttcatgtaag ttaactgata aattcttctc gcgatgggga
8011-2 tggtttggag ggcggttagc ttttgctaca caacactggc aaagttttac ctcgttatcg ttcatgtaag ttaactaata aattcttctc gcgatgggga
8011-6 tggtttggag ggcggttagc ttttgctaca caacactggc aaagttttac ctcgttatcg ttcatgtaag ttaactaata aattcttctc gcgatgggga
8011-3 tggtttggag ggcggttagc ttttgctaca caacactggc aaagttttac ctcgttatcg ttcatgtaag ttaactaata aattcttctc gcgatgggga
8011-4 tggtttggag ggcggttagc ttttgctaca caacactggc aaagttttac ctcgttatcg ttcatgtaag ttaactaata aattcttctc gcgatgggg-
8011-5 tggtttggag ggcggttagc ttttgctaca caacactggc aaagttttac ctcgttatcg ttcatgtaag ttaactaata aattcttctc gcgatgggga
8011-0 tggtttggag ggcggttagc ttttgctaca caacactggc aaagttttac ctcgttatcg ttcatgtaag ttaactaata aattcttctc gcgatgggga

       1111111111 1111111111 1111111111 1111111111 1111111111 1111111111 1111111111 1111111111 1111111111 1111111112
       0000000001 1111111112 2222222223 3333333334 4444444445 5555555556 6666666667 7777777778 8888888889 9999999990
       1234567890 1234567890 1234567890 1234567890 1234567890 1234567890 1234567890 1234567890 1234567890 1234567890
8011-1 ccgcagtggg atatgcactg agagaacttt tagtttatct atcagctgtc gccatgatat tatttgcgat cgcgcagatg actctgttga aactcctgtc
8011-2 ccgcagtggg atatgcactg agaaaacttt tagtttatct atcagctgtc gccatgatat tatttgcgat cgcgcagatg actctgttga aactcctgtc
8011-6 ccgcagtggg atatgcactg agaaaacttt tagtttatct atcagctgtc gccatgatat tatttgcgat cgcgcagatg actctgttga aactcctgtc
8011-3 ccgcagtggg atatgcactg agaaaacttt tagtttatat atcagctgtc gccatgatat tatttgcgat cgcgcagatg actctgttga aactcctgtc
8011-4 ccgcagtggg atatgcactg agaaaacttt tagtttatct atcagctgtc gccatgatat tatttgcgat cgcgcatttg actctgttga aactcctgtc
8011-5 ccgcagtggg atatgcactg agaaaacttt tagtttatct atcagctgtc gccatgatat tatttgcgat cgcgcatttg actctgttga aactcctgtc
```

```
8011-0 cagcggtggg atatgcgctc agaaaacttt ctgtttatgt atcagctgtg gccatgatat tatttgcgat cgtgaatgta accctgttga aactcctgtc

       2222222222 2222222222 2222222222 2222222222 2222222222 2222222222 2222222222 2222222222 2222222222 2222222223
       0000000001 1111111112 2222222223 3333333334 4444444445 5555555556 6666666667 7777777778 8888888889 9999999990
       1234567890 1234567890 1234567890 1234567890 1234567890 1234567890 1234567890 1234567890 1234567890 1234567890
8011-1 agt-cgtttt cccgggactg atgaagaaaa tccatcttaa aatgggagag aagtttacga tgacccaaaa tcccaaattc agatacgaag actggggtcc
8011-2 agt-cgtttt cccgggactg atgaagaaaa tccatcttaa aatgggagag aagtttacga tgacccaaaa tcccaaattc agatacgaag actggggtcc
8011-6 agt-cgtttt cccgggactg atgaagaaaa tccatcttaa aatgggagag aagtttacga tgacccaaaa tcccaaattc agatacgaag actggggtcc
8011-3 agt-cgtttt cccgggactg atgaagaaaa tccatcttaa aatgggagag aagtttacga tgacccaaaa tcccaaattc agatacgaag actggggtcc
8011-4 agt-cgtttt cccgggactg atgaagaaaa tccatcttaa aatgggagag aagtttacga tgacccaaaa tcccaaattc agatacgaag actggggtcc
8011-5 agt-cgtttt cccgggactg atgaagaaaa tccatcttaa aatgggagag aagtttacga tgacccaaaa tcccaaattc agatacgaag actggggtcc
8011-0 ctttcgtttc cccgggactg atgaggaaaa tccatctaaa aatgggagag aagtcgacga tgactcaaaa ccccaaattc agatacgaag actggggtcc

       333
       000
       123
8011-1 gac
8011-2 gac
8011-6 gac
8011-3 gac
8011-4 gac
8011-5 gac
8011-0 gac
```

SNP LOCATIONS: 177G/T, 178A/T

35.

```
                1 1111111112 2222222223 3333333334 4444444445 5555555556 6666666667 7777777778 8888888889 9999999990
       1234567890 1234567890 1234567890 1234567890 1234567890 1234567890 1234567890 1234567890 1234567890 1234567890
8012-0 cagagtccac ggcaagtatg attcttctgc acaatctgac tttgctgcag cctggaattg aactgctggt ttcgtctggt cagaggagaa ctggcccccc
```

```
8012-4 cagagtccac ggcaagtatg attcttctgc acagtctgac tttgctgcag cctggaattg aactgctggt tttgtctggt cagagtagaa ctggcccccc
8012-6 cagagtccac ggcaagtatg attcttctgc acaatctgac tttactgcag cctggaattg aactgctggt ttcgtctggt cagaggagaa ctggcccccc
8012-3 cagagtccac ggcaagtatg attcttctgc acaatctgac tttactgcag cctggaattg aactgctggt ttcgtctggt cagaggagaa ctggcccccc
8012-1 cagagtccac ggcaagtatg attcttctgc acaatctgac tttactgcag cctggaattg aactgctggt ttcgtctggt cagaggagaa ctggcccccc
8012-2 cagagtccac ggcaagtatg attcttctgc acaatctgac tttactgcag cctggaattg aactgctggt ttcgtctggt cagaggagaa ctggcccccc
8012-7 cagagtccac ggcaagtatg attcttctgc acaatctgac tttactgcag cctggaattg aactgctggt ttcgtctggt cagaggagaa ctggcccccc
8012-5 cagagtccac ggcaagtatg attcttctgc acaatctgac tttactgcag cctggaattg aactgctgat ttcgtctggt cagaggagaa ctggccccct

       1111111111 1111111111 1111111111 1111111111 1111111111 1111111111 1111111111 1111111111 1111111111 1111111112
       0000000001 1111111112 2222222223 3333333334 4444444445 5555555556 6666666667 7777777778 8888888889 9999999990
       1234567890 1234567890 1234567890 1234567890 1234567890 1234567890 1234567890 1234567890 1234567890 1234567890
8012-0 gactgagcct ggtttctccc aaggtttttt -ctccattct gtcaccgatg gagttttggt tccttgccgc tgttgcctct ggcttgctta gttggggaca
8012-4 gactgagcct ggtttctccc aaggtttttt -ctccattct gtcaccgatg gagttttggt tccttgccgc tgttgcctct ggcttgctta gttggggaca
8012-6 gactgagcct ggtttctccc aaggtttttt -ctccattct gtcaccgatg gagttttggt tccttgccgc tgttgcctct ggcttgctta gttggggaca
8012-3 gactgagcct ggtttctccc aaggtttttt -ctccattct gtcaccgatg gagttttggt tccttgccgc tgttgcctct ggcttgctta gttggggaca
8012-1 gactgagcct ggtttctccc aaggtttttt -ctccattct gtcaccgatg gagttttggt tccttgccgc tgttgcctct ggcttgctta gttggggaca
8012-2 gactgagcct ggtttctccc aaggtttttt tctccattct gtcaccgatg gagttttggt tccttgccgc tgttgcctct ggcttgctta gttggggaca
8012-7 gactgagcct ggtttcttcc aaggtttttt tctccattct gtcaccgatg gagttttggt tccttgccgc tgttgcctct ggcttgctta gttggggaca
8012-5 gactgagcct ggtttctccc aaggtttttt tctccattct ctcactgatg gaattttggt tccttgccgc tgtcgtctct ggcttgctta gttggggaca

       2222
       0000
       1234
8012-0 cttc
8012-4 cttc
8012-6 cttc
8012-3 cttc
8012-1 cttc
8012-2 cttc
8012-7 cttc
```

```
8012-5 cttc

SNP LOCATIONS: 44A/G

36.
                1 1111111112 2222222223 3333333334 4444444445 5555555556 6666666667 7777777778 8888888889 9999999990
       1234567890 1234567890 1234567890 1234567890 1234567890 1234567890 1234567890 1234567890 1234567890 1234567890
8013-6 acaccttccc ccagtaaacc tcctgagact acaaagcctc ctcccagtgc tgcaggactg ggtcctctgc ccaccgtgct gtgccttctg atcccagcag
8013-7 acaccttccc ccagtaaacc tcctgagact acaaagcctc ctcccagtgc tgcaggactg ggtcctctgc ccaccgtgct gtgccttctg atcccagcag
8013-5 acaccttccc ccagtaaacc tcctgagact acaaagcctc ctcccagtgc tgcaggactg ggtcctctgc ccaccgtgct gtgccttctg atcccagcag
8013-2 acaccttccc ccagtaaacc tcctgagact acaaagcctc ctcccagtgc tgcaggactg ggtcctctgc ccaccgtgct gtgccttctg atcccagcag
8013-0 acaccttccc ccagtaaacc tcctgagact acaaagcctc ctcccagtgc tgcaggactg ggtcctctgc ccaccgtgct gtgccttctg atcccagcag
8013-4 acaccttccc ccagtaaacc tcctgagact acaaagcctc ctcccagtgc tgcaggactg ggtcctctgc ccaccgtgct gtgccttctg atcccagcag
8013-1 acaccttccc ccagtaaacc tcctgagact acaaagcctc ctcccagtgc tgcaggactg agtcctctgc ccaccgtgct gtgccttctg atcccagcag
8013-3 acaccttccc ccagtaaacc tcctgagact acaaagcctc ctcccagtgc tgcaggactg agtcctctgc ccaccgtgct gtgccttctg atcccagcag

       1111111111 1111111111 1111111111 1111111111 1111111111 1111111111 1111111111 1111111111 1111111111 1111111112
       0000000001 1111111112 2222222223 3333333334 4444444445 5555555556 6666666667 7777777778 8888888889 9999999990
       1234567890 1234567890 1234567890 1234567890 1234567890 1234567890 1234567890 1234567890 1234567890 1234567890
8013-6 ccaccctggg cttcattcac agtagattct aaactggctg gagaatgttt agatgaccaa gaggagcaaa gatagatagc cagtcacatg aagaccaaac
8013-7 ccaccctggg cttcattcac agtagattct aaactggctg gagaatgttt agatgaccaa gaggagcaaa gatagatagc cagtcacatg aagaccaaac
8013-5 ccaccctggg cttcattcac agtagattct aaactggctg gagaatgttt agatgaccaa gaggagcaaa gatagatagc cagtcacatg aagaccaaac
8013-2 ccaccctggg cttcattcac agtagattct aaactggctg gagaatgttt agatgaccaa gaggagcaaa gatagatagc cagtcacatg aagaccaaac
8013-0 ccaccctggg cttcattcac agtagattct aaactggctg gagaatgttt agatgaccaa gaggagcaaa gatagatagc cagtcacatg aagaccaaac
8013-4 ccaccctggg cttcattcac agtagattct aagctggctg gagaatgttt agatgaccaa gaggagcgaa gatagatagc cagtcacatg aagaccaaac
8013-1 ccaccctggg cttcatccac agtagattct aaactggctg gagaatgttt agatgaccaa gaggagcaaa gatagatagc cagtcacatg aagaccaaac
8013-3 ccaccctggg cttcatccac agtagattct aaactggctg gagaatgttt agatgaccaa gaggagcaaa gatagatagc cagtcacatg aagaccaaac

       2222222222 2222222222 2222222222 2222222222 2222222222 2222222222
       0000000001 1111111112 2222222223 3333333334 4444444445 5555555556
```

```
        1234567890 1234567890 1234567890 1234567890 1234567890 1234567890
8013-6 ttttccaaaa ttctttaaga tagaagacac a---agaaca actcaacaag aagatgccac
8013-7 ttttccaaaa ttctttaaga tagaagacac a---agaaca actcaacaag aagatgccac
8013-5 ttttccaaaa ttctttaaga tagaagacac a---agaaca actcaacaag aagatgccac
8013-2 ttttccaaaa ttctttaaga tagaagacac a---agaaca actcaacaag aagatgccac
8013-0 ttttccaaaa ttctttaaga tagaagacac agcaagaaca actcaacaag aagatgccac
8013-4 ttttccaaaa ttctttaaga tagaagacac a---agaaca actcaacaag aagatgccac
8013-1 ttttccaaaa ttctttaaga tagaagacac a---agaaca actcaacaag aagatgccac
8013-3 ttttccaaaa ttctttaaga tagaagacac a---agaaca actcaacaag aagatgccac
```

SNP LOCATIONS: 61G/A, 117T/C

37.

```
                1 1111111112 2222222223 3333333334 4444444445 5555555556 6666666667 7777777778 8888888889 9999999990
       1234567890 1234567890 1234567890 1234567890 1234567890 1234567890 1234567890 1234567890 1234567890 1234567890
8017-1 catttctgag accttcaacc atgctaatgg cctgacgctg gtgtctcgcg ctcatcagct ggtcatggag gtaagatcca caattcaact cttcacaacc
8017-2 catttctgag accttcaacc atgctaatgg cctgacgctg gtgtctcgcg ctcatcagct ggtcatggag gtaagatcca caattcaact cttcacaacc
8017-5 catttctgag accttcaacc atgctaatgg cctgacgctg gtgtctcgcg ctcatcagct ggtcatggag gtaagatcca caattcaact cttcacaacc
8017-3 catttctgag accttcaacc atgctaatgg cctgacgctg gtgtctcgcg ctcatcagct ggtcatggag gtaagatcca caattcaact cttcgcaacc
8017-4 catttctgag accttcaacc atgctaatgg cctgacgctg gtgtctcgcg ctcatcagct ggtcatggag gtaaggtcca caattcaact cttcacaacc

       1111111111 1111111111 1111111111 1111111111 1111111111 1111111111 1111111111 1111111111 1111111111 1111111112
       0000000001 1111111112 2222222223 3333333334 4444444445 5555555556 6666666667 7777777778 8888888889 9999999990
       1234567890 1234567890 1234567890 1234567890 1234567890 1234567890 1234567890 1234567890 1234567890 1234567890
8017-1 cttcagtggg atgaagataa tcagatatca aataaaactt tttttttttc tttttctttt ttcctttcag ggatacaact ggtgtcatga taggaacgtt
8017-2 cttcagtggg atgaagataa tcagatatca aataaaactt tttttttttc tttttctttt ttcctttcag ggatacaact ggtgtcatga taggaacgtt
8017-5 cttcagtggg atgaagataa tcagatatca aataaaactt tttttttt-c tttttctttt ttcctttcag ggatacaact ggtgtcatga taggaacgtt
8017-3 cttcagtggg atgaagataa tcagatatca aataaaactt tttttttttc tttttctttt ttcctttcag ggatacaact ggtgtcatga taggaacgtt
8017-4 cttcagtggg atgaagataa tcagatatca aataaaactt tttttttttc tttttctttt ttcctttcag ggatacaact ggtgtcatga taggaacgtt
```

```
       2222222222 2222222222 2222222222 2222222222 2222222222 2222222222 2222222222 2222222222 2222222222 2222222223
       0000000001 1111111112 2222222223 3333333334 4444444445 5555555556 6666666667 7777777778 8888888889 9999999990
       1234567890 1234567890 1234567890 1234567890 1234567890 1234567890 1234567890 1234567890 1234567890 1234567890
8017-1 gtcactatct tcagtgcgcc aaactactgc tatcgctgtg gaaaccaggc agccattatg gaactggatg atactcttaa atattcgttg taagttgcgc
8017-2 gtcactatct tcagtgcgcc aaactactgc tatcgctgtg gaaaccaggc agccattatg gaactggatg atactcttaa atattcgttg taagttgcgc
8017-5 gtcactatct tcagtgcgcc aaactactgc tatcgctgtg gaaaccaggc agccattatg gaactggatg atactcttaa atattcgttg taagttgcgc
8017-3 gtcactatct tcagtgcgcc aaactactgc tatcgctgtg gaaaccaggc agccattatg gaactggatg atactcttaa atattcgttg taagttgcgc
8017-4 gtcactatct tcagtgcgcc aaactactgc tatcgctgtg gaaaccaggc agccattatg gaactggatg atactcttaa atattcgttg taagttgcgc

       3333333333 3333333333 3333333333 3333333333 3333333333 3333333333 3333333333 3333333333 3333333333 3333333334
       0000000001 1111111112 2222222223 3333333334 4444444445 5555555556 6666666667 7777777778 8888888889 9999999990
       1234567890 1234567890 1234567890 1234567890 1234567890 1234567890 1234567890 1234567890 1234567890 1234567890
8017-1 ttctcattat aatctttcaa atatgtacta tcatgtaagg tgtaatttca acaacatgtc tgaattatat ttcagttggt tcacttcaaa aataataaag
8017-2 ttctcattat aatctttcaa atatgtacta tcatgtaagg tgtaatttca acaacatgtc tgaattatat ttcagttggt tcacttcaaa aataataaag
8017-5 ttctcattat aatctttcaa atatgtacta tcatgtaaag tgtaatttca acaacatgtc tgaattatat ttcagttggt tcactccaaa aataataaag
8017-3 ttctcattat aatctttcaa atatgtacta tcatgtaaag tgtaatttca acaacatgtc tgaattatat ttcagttggt tcacttcaaa aataataaag
8017-4 ttctcattat aatctttcaa atatgtacta tcatgtaaag tgtaatttca acaacatgtc tgaattatat ttcagttggt tcacttcaaa aataataaag

       4444444444 4444444444 4444444444 4444444444 4444444444 4444444444 4444444444 4444444444 4444444444 4444444445
       0000000001 1111111112 2222222223 3333333334 4444444445 5555555556 6666666667 7777777778 8888888889 9999999990
       1234567890 1234567890 1234567890 1234567890 1234567890 1234567890 1234567890 1234567890 1234567890 1234567890
8017-1 cctatttcta aaactataat ggttttgcat ctttttggca ttactttaaa attgtgactg ttaacccaaa aatattttac aggaatattc ttattgctgc
8017-2 cctatttcta aaactataat ggttttgcat ctttttggca ttactttaaa attgtgactg ttaacccaaa aatattttac aggaatattc ttattgctgc
8017-5 cctatttcta aaactataat ggttttgcat ctttttggca ttactttaaa attgtgactg tcaacccaaa aatattttac aggaatattc ttattgctgc
8017-3 cctatttcta aaactataat ggttttgcat ctttttggca ttactttaaa attgtgactg ttaacccaaa aatattttac aggaatattc ttattgctgc
8017-4 cctatttcta aaattataat ggttttgcat ctttttggca ttactttaaa attgtgactg ttaacccaaa aatattttac aggaatattc ttattgctgc

       5555555555 5555555555 5555555555 5555555555 5555555555 5555555555 5555555555 5555555555 5555555555 5555555556
       0000000001 1111111112 2222222223 3333333334 4444444445 5555555556 6666666667 7777777778 8888888889 9999999990
       1234567890 1234567890 1234567890 1234567890 1234567890 1234567890 1234567890 1234567890 1234567890 1234567890
```

8017-1 aatgcaaaca gtcttgaagt gaagtgaagt atgtaattgc catcgaaatg ccacttgcag gaatgttttc tgaacaccca tggaagtcca aaaaagacct
8017-2 aatgcaaaca gtcttgaagt gaagtgaagt atgtaattgc catcgaaatg ccacttgcag gaatgttttc tgaacaccca tggaagtcca aaaaagacct
8017-5 aatgcaaaca gccttgaagt gaagtgaagt atgtaattgc catcgaaatg ccacttgcag gaatgttttc tgaacaccca tggaagtcca aaaa--acct
8017-3 aatgcaaaca gtcttgaagt gaagtgaagt atgtaattgc catcgaaatg ccacttgcag gaatgttttc tgaacaccca tggaagtcca aaaaaaacct
8017-4 aatgcaaaca gtcttgaagt gaagtgaagt atgtaattgc catcgaaatg ccacttgcag gaatgttttc tgaacaccca tggaagtcca aaaaaaacct

6666666666 6666666666 6666666666 6666666666 6666666666 6666666666 6666666666 6666666666 6666666666 6666666667
0000000001 1111111112 2222222223 3333333334 4444444445 5555555556 6666666667 7777777778 8888888889 9999999990
1234567890 1234567890 1234567890 1234567890 1234567890 1234567890 1234567890 1234567890 1234567890 1234567890
8017-1 tcctgcaagt cttttcatag caccagtgtt gggcacttca gggaaatcaa cctacaaatg tctcactgcg tattaggtct gaaacgatta gtggaatagt
8017-2 tcctgcaagt cttttcatag caccagtgtt gggcacttca gggaaatcaa cctacaaatg tctcactgcg tattaggtct gaaacgatta gtggaatagt
8017-5 tcctgcaagt cttttcatag caccagtgtt ggacacttca gggaaatcaa cctacaaatg tctcactgcg tattaggtct gaaacgatta gtcgaatagt
8017-3 tcctgcaagt cttttcatag caccagtgtt ggacacttca gggaaatcaa cctacaaatg tctcactgcg tattaggtct gaaacgatta gtcgaatagt
8017-4 tcctgcaagt cttttcatag caccagtgtt ggacacttca gggaaatcaa cctacaaatg tctcactgcg tattaggtct gaaacgatta gtcgaatagt

7777777777 7777777777 7777777777 7777777777 7777777777 7777777777 7777777777 7777777777 7777777777 7777777778
0000000001 1111111112 2222222223 3333333334 4444444445 5555555556 6666666667 7777777778 8888888889 9999999990
1234567890 1234567890 1234567890 1234567890 1234567890 1234567890 1234567890 1234567890 1234567890 1234567890
8017-1 tgtttgatct catttgactt aatgaaagag cctgcagtaa tgcggtttga ccagtgtagt gcaagactgt aatgcaatcg aacctgacgc tgctgaaaac
8017-2 tgtttgatct catttgactt aatgaaagag cctgcagtaa tgcggtttga ccagtgtagt gcaagactgt aatgcaatcg aacctgacgc tgctgaaaac
8017-5 tgtttgatct catttgactt aatgaaagag cctgcagtaa tgcggtttga ccagtgtagt gcaagactgt aatgcaatcg aacctgacgc tgctgaaaac
8017-3 tgtttgatct catttgactt aatgaaagag cctgcagtaa tgcggtttga ccagtgtagt gcaagactgt aatgcaatcg aacctgacgc tgctgaaaac
8017-4 tgtttgatct catttgactt aatgaaagag cctgcagtaa tgcggtttga ccagtgtagt gcaagactgt aatgcaatcg aacctgacgc tgctgaaaac

8888888888 8888888888 8888888888 8888888888 8888888888 8888888888 8888888888 8888888888 8888888888 8888888889
0000000001 1111111112 2222222223 3333333334 4444444445 5555555556 6666666667 7777777778 8888888889 9999999990
1234567890 1234567890 1234567890 1234567890 1234567890 1234567890 1234567890 1234567890 1234567890 1234567890
8017-1 cgagtttata tgaatatgta aatatgtgct gcatgctttc ctcttaataa tgaaataaac acacaacaaa aattagagcg gtgagaaaac agtagttaag
8017-2 cgagtttata tgaatatgta aatatgtgct gcatgctttc ctcttaataa tgaaataaac acacaacaaa aattagagcg gtgagaaaac agtagttaag
8017-5 cgagtttata tgaatatgta aatatgtgct gcatgctttc ctcttaataa tgaaataaac acacaacaaa aattagagcg gtgagaaaac agtagttaag

```
8017-3 cgagtttata tgaatatgta aatatgtgct gcatgctttc ctcttaataa tgaaataaac acacaacaaa aattagagcg gtgagaaaac agtagttaag
8017-4 cgagtttata tgaatatgta aatatgtgct gcatgctttc ctcttaataa tgaaataaac acacaacaaa aattagagcg gtgagaaaac agtagttaag

                                                                                                                1
       9999999999 9999999999 9999999999 9999999999 9999999999 9999999999 9999999999 9999999999 9999999999 9999999990
       0000000001 1111111112 2222222223 3333333334 4444444445 5555555556 6666666667 7777777778 8888888889 9999999990
       1234567890 1234567890 1234567890 1234567890 1234567890 1234567890 1234567890 1234567890 1234567890 1234567890
8017-1 aaagcatctg ttcatagcaa tgtggacaag tttgcaccag ctctgcgatc ggctctagag tcacgtcatg tttatttata tagtgtttta tacaatagat
8017-2 aaagcatctg ttcatagcaa tgtggacaag tttgcaccag ctctgcgatc ggctctagag tcacgtcatg tttatttata tagtgtttta tacaatagat
8017-5 aaagcatctg ttcatagcaa tgtggacaag tttgcactag ctctgcaatc ggctctagag tcacgtcatg tttatttata tagtgtttta tacaatagat
8017-3 aaagcatctg ttcatagcaa tgtggacaag tttgcactag ctctgcaatc ggctctagag tcacgtcatg tttatttata tagtgtttta tacaatagat
8017-4 aaagcatctg ttcatagcaa tgtggacaag tttgcactag ctctgcaatc ggctctagag tcacgtcatg tttatttata tagtgtttta tacaatagat

       1111111111 1111111111 1111111111 1111111111 1111111111 1111111111 1111111111 1111111111 1111111111 1111111111
       0000000000 0000000000 0000000000 0000000000 0000000000 0000000000 0000000000 0000000000 0000000000 0000000001
       0000000001 1111111112 2222222223 3333333334 4444444445 5555555556 6666666667 7777777778 8888888889 9999999990
       1234567890 1234567890 1234567890 1234567890 1234567890 1234567890 1234567890 1234567890 1234567890 1234567890
8017-1 tgatttaacg catgcagctt tacagtatta aacatgaaaa aaaaaa--ct cagtgtcagt ctcactttca gttagactta caactagggt tgggcgatgt
8017-2 tgatttaacg catgcagctt tacagtatta aacatgaaaa aaaaaa--ct cagtgtcagt ctcactttca gttagactta caactagggt tgggcgatgt
8017-5 tgatttaacg catgcagctt tacagtatta aacatgaaaa aaaaaa--ct cagtgtcagt ctcactttca gttagactta caactagggt tgggcgatgt
8017-3 tgatttaacg catgcagctt tacagtatta aacatgaaaa aaaaaaa-ct cagtgtcagt ctcactttca gttagactta caactagggt tgggcgatgt
8017-4 tgatttaacg catgcagctt -acagtatta aacatgaaaa aaaaaaaact cagtgtcagt ctcactttca gttagactta caactagggt tgggcgatgt

       1111111111 1111111111 1111111111 1111111111 1111111111 1111111111 1111111111 1111111111 1111111111 1111111111
       1111111111 1111111111 1111111111 1111111111 1111111111 1111111111 1111111111 1111111111 1111111111 1111111112
       0000000001 1111111112 2222222223 3333333334 4444444445 5555555556 6666666667 7777777778 8888888889 9999999990
       1234567890 1234567890 1234567890 1234567890 1234567890 1234567890 1234567890 1234567890 1234567890 1234567890
8017-1 ctacaaattt ggcattggac gatgtttaca a--------- ---------- ---------- ---------- ---------- ---------- ----------
8017-2 ctacaaattt ggcattggac gatgtttaca a--------- ---------- ---------- ---------- ---------- ---------- ----------
8017-5 ctacaaattt ggcattggac gatgtttaca a--------- ---------- ---------- ---------- ---------- ---------- ----------
```

```
8017-3 ctacaaattt ggcattggac gatgtttaca a--------- ---------- ---------- ---------- ---------- ---------- ----------
8017-4 ctacaaattt ggcattggac gatgtctaca aatttggcat tggacgatgt ctacaaattt ggcattggac gatgtctaca aatttggcat tggacgatgt

       1111111111 1111111111 1111111111 1111111111 1111111111 1111111111 1111111111 1111111111 1111111111 1111111111
       2222222222 2222222222 2222222222 2222222222 2222222222 2222222222 2222222222 2222222222 2222222222 2222222223
       0000000001 1111111112 2222222223 3333333334 4444444445 5555555556 6666666667 7777777778 8888888889 9999999990
       1234567890 1234567890 1234567890 1234567890 1234567890 1234567890 1234567890 1234567890 1234567890 1234567890
8017-1 ---------- ---------- ---------- -tgaaacatt gtgatggacg atgacattgg tgggggcggg ggttttgtgc ccgaagcgtg aattataagg
8017-2 ---------- ---------- ---------- -tgaaacatt gtgatggacg atgacattgg tgggggcggg ggttttgtgc ccgaagcgtg aattataagg
8017-5 ---------- ---------- ---------- -tgaaacatt gtgatggacg atgacattgg tgggggcggg ggttttgtgc ccgaagcgtg aattataagg
8017-3 ---------- ---------- ---------- -tgaaacatt gtgatggacg atgacattgg tgggggcggg ggttttgtgc ccgaagcgtg aattataagg
8017-4 ctacaaattt ggcattggac gatgtctaca atgaaacatt gtgatggacg atgatattgg tgggggcggg ggttttgtgc ccgaagcgtg aattataagg

       1111111111 1111111111 1111111111 1111111111 1111111111 1111111111 1111111111 1111111111 1111111111 1111111111
       3333333333 3333333333 3333333333 3333333333 3333333333 3333333333 3333333333 3333333333 3333333333 3333333334
       0000000001 1111111112 2222222223 3333333334 4444444445 5555555556 6666666667 7777777778 8888888889 9999999990
       1234567890 1234567890 1234567890 1234567890 1234567890 1234567890 1234567890 1234567890 1234567890 1234567890
8017-1 gaaaacaacc aacgattcga cagtgagctg tacatttaag ccacttttct tcttttcccc agccttcagt ttgatccagc tcctcgacgc ggagaacctc
8017-2 gaaaacaacc aacgattcga cagtgagctg tacatttaag ccacttttct tcttttcccc agccttcagt ttgatccagc tcctcgacgc ggagaacctc
8017-5 gaaaacaacc aacgattcga cagtgagctg tacatttaag ccacttttct tcttttcccc agccttcagt ttgatccagc tcctcgacgc ggagaacctc
8017-3 gaaaacaacc atcgattcga cagtgagctg tacatttaag ccacttttct tcttttcccc agccttcagt ttgatccagc tcctcgacgc ggagaacctc
8017-4 gaaaacaacc aacgattcga cagtgagctg tacatttaag ccacttttct tcttttcccc agccttcagt ttgatccagc tcctcgacgt ggagaacttc

       1111111111 1111111111 1111111111 1111111111 1111111111 1111111111 1111111111 1111111111 111111
       4444444444 4444444444 4444444444 4444444444 4444444444 4444444444 4444444444 4444444444 444444
       0000000001 1111111112 2222222223 3333333334 4444444445 5555555556 6666666667 7777777778 888888
       1234567890 1234567890 1234567890 1234567890 1234567890 1234567890 1234567890 1234567890 123456
8017-1 acgtcacccg ccgcaccccc gactacttcc tataagtgag cactggcctg ccacgaaaga gctctgaagc aagcagaaaa tgacaa
8017-2 acgtcacccg ccgcaccccc gactacttcc tataagtgag cactggcctg ccacgaaaga gctctgaagc aagcagaaaa tgacaa
8017-5 acgtcacccg ccgcaccccc gactacttcc tataagtgag cactggcctg tcacgaagga gctctgaagc aagcagaaaa tgacaa
```

```
8017-3 acgtcacccg ccgcaccccc gactacttcc tataagtgag cactggcctg ccacgaaaga gctctgaagc aagcagaaaa tgacaa
8017-4 acgtcacccg ccgcaccccc gactacttcc tataagtgag cactggcctg ccacgaaaga gctctgaagc aagcagaaaa tgacaa
```

SNP LOCATIONS: 339A/G, 596A/G, 633A/G, 693C/G, 938T/C, 947A/G

38.

```
                1 1111111112 2222222223 3333333334 4444444445 5555555556 6666666667 7777777778 8888888889 9999999990
       1234567890 1234567890 1234567890 1234567890 1234567890 1234567890 1234567890 1234567890 1234567890 1234567890
8021-2 tctgtcactt tagatcctgg tgtagtaaaa caaccaaagc atgtgatgat gtggtatttt aatgacactc tcattgctga aatcactgga gatcccaaca
8021-4 tctgtcactt tagatcctgg tgtagtaaaa caaccaaatc atgtgatgac gtggtatttt aatgacactc tcattgctga aatcactgga gatcccaaca
8021-1 tctgtcactt tagatcctgg tgtagtaaaa caaccaaatc atgtgatgat gtggtatttt aatgacactc tcatcgctga aatcactgga gatcccaaca
8021-3 tctgtcactt tagatcctgg tgtagtaaaa caaccaaatc atgtgatgac gtggtatttt aatgacactc tcatcgctga aatcactgga gatcccaaca
8021-5 tctgtcactt tagatcctgg tgtagtaaaa caaccaaatc atgtgatgat gtggtatttt aatgacactc tcatcgctga aatcactgga gatcccaaca
8021-0 tctgtcactt tagatcctgg tgtagtaaga caaccaaatg aggtgatgag atggtattat aaagacactc tcatcgctga aatcactcaa tatctgagta

       1111111111 1111111111 1111111111 1111111111 1111111111 1111111111 1111111111 1111111111 1111111111 1111111112
       0000000001 1111111112 2222222223 3333333334 4444444445 5555555556 6666666667 7777777778 8888888889 9999999990
       1234567890 1234567890 1234567890 1234567890 1234567890 1234567890 1234567890 1234567890 1234567890 1234567890
8021-2 aggcctgcac agactttcat tgtcctgaga gattcagaga cagactgcag ctggatcatc agactggatc tctgaccatc aggcacatca gaatcacaga
8021-4 aggcctgcac agactttcat tgtcctgaga gattcagaga cagactgcag ctggatcatc agactggatc tatgaccatc aggcacatca gaatcacaga
8021-1 aggcctgcac agactttcat tgtcctgaga gattcagaga cagactgcag ctggatcatc agactggatc tctgaccatc aggaacatca gagtcacaga
8021-3 aggcctgcac agactttcat tgtcctgaga gattcagaga cagactgcag ctggatcatc agactggatc tctgactatc aggaacatca gagtcacaga
8021-5 aggcctgcac agactttcat tgtcctgaga gattcagaga cagactgcag ctggatcatc agactggatc tctgaccatc aggcacatca gaatcacaga
8021-0 acgtctgtac agatgatgag tgtaaagaga gattcagaga cagactgcag ctggatcatc agactggatc tctgaccatc aggcacatca gaatcacaga

       2222222222 2222222222 2222222222 2222222222 2222222222 2222222222 2222222222 2222222222 2222222222 2222222223
       0000000001 1111111112 2222222223 3333333334 4444444445 5555555556 6666666667 7777777778 8888888889 9999999990
       1234567890 1234567890 1234567890 1234567890 1234567890 1234567890 1234567890 1234567890 1234567890 1234567890
8021-2 ttctggactt tatgaactaa agatcagcag cagcagtcgc cgccgccgcc gcagcatcag cagtgtaaag agcttcgatg ttactgtcat tagtaagtgt
8021-4 ttctggactt tatgaactaa agatcagcag cagcagtcgc cgccgccgcc gcagcatcag cagtgtaaag agcttcgatg ttactgtcat tagtaagtgt
```

```
8021-1 ctctggactt tatgaactaa agatcagcag cagcagtcgc cgccgccgcc gcagcatcag cagtgtaaag agcttcgatg ttactgtcat tagtaagtgt
8021-3 ctctggactt tatgaactaa agatcagcag cagcagtcgc cgccgccgcc gcagcatcag cagtgtaaag agcttcgatg ttactgtcat tagtaagtgt
8021-5 ctctggactt tatgaactaa agatcagcag cagcagtcgc cgccgccgcc gcagcatcag cagtgtaaag agcttcgatg ttactgtcat tagtaagtgt
8021-0 ctctggactt tatgaactaa agatcagcag cagcagtcgc cgccgccgcc gcagcatcag cagtgtaaag agcttcgatg ttactgtcat ta--------

       3333333333 3333333333 3333333333 3333333333 3333333333 3333333333 3333333333 3333333333 3333333333 3333333334
       0000000001 1111111112 2222222223 3333333334 4444444445 5555555556 6666666667 7777777778 8888888889 9999999990
       1234567890 1234567890 1234567890 1234567890 1234567890 1234567890 1234567890 1234567890 1234567890 1234567890
8021-2 aatttagtcc ttcaggcatt tggactcata cagtggaata taaagaatga tatagatcag tctgactcat tcattcatac acactaatga cggtctctgt
8021-4 aatttagtcc ttcaggcatt tggactcata cagtggaata taaagaatga tatagatcag tctgactcat tcattcatac acactaatga cggtctctgt
8021-1 aatttagtcc ttcaggcatt tggactcata cagtggaata taaagaatga tatagatcag tctgactcat tcattcatac acactaatga cggtctctgt
8021-3 aatttagtcc tccaggcatt tggactcata gagtggaata taaagaatga tatagatcag tctgactcat tcattcatac acactaatga cggtctctgt
8021-5 aatttagtcc ttcaggcatt tggactcata cagtggaata caaagaatga tatagatcag tctgactcat tcatgcatac acactaatga cggtctctgt
8021-0 ---------- ---------- ---------- ---------- ---------- ---------- ---------- ---------- ---------- ----------

       4444444444 4444444444 4444444444 4444444444 4444444444 4444444444 4444444444 4444444444 4444444444 4444444445
       0000000001 1111111112 2222222223 3333333334 4444444445 5555555556 6666666667 7777777778 8888888889 9999999990
       1234567890 1234567890 1234567890 1234567890 1234567890 1234567890 1234567890 1234567890 1234567890 1234567890
8021-2 tttgttccag attcaggtct gtctccgggt gctgtagcag gtctgtctgt tattgttctt ctactggtga ctgcagctgt agctgttggt gtgattttct
8021-4 tttgttccag attcaggtct gtctccgggt gctgtagcag gtctgtctgt tattgttctt ctactggtga ctgcagctgt agctgttggt gtgattttct
8021-1 tttgttccag attcaggtct gtctctgggt gctgtagcag gtctgtctgt tattgttctt ctactgatga ctgcagctgt agctgttggt gtgattttct
8021-3 tttgctccag attcaggtct gtctctgggt gctgtagcag gtctgtctgt tattgttctt ctactggtga ctgcagctgt agctgttggt gtgattttct
8021-5 tttgttccag attcaggtct gtctctgggt gctgtagcag gtctgtctgt tattgttctt ctactggtga ctgcagctgt agctgttggt gtgattttct
8021-0 ---------- attcaggtct gtctctgggt gctgtagcag gtctgtctgt tattgttctt ctactggtga ctgcagctgt agctgttggt gtgatttttt

       5555555555 5555555555 5555555555 5555555555 5555555555 5555555555 5555555555 5555555555 5555555555 5555555556
       0000000001 1111111112 2222222223 3333333334 4444444445 5555555556 6666666667 7777777778 8888888889 9999999990
       1234567890 1234567890 1234567890 1234567890 1234567890 1234567890 1234567890 1234567890 1234567890 1234567890
8021-2 atcaccgcag gaattccaga aatggcaa-- gtatcaccta ttaatactac attgagagga aatcatcacc tttattgagc aaaatctgta tttaattatg
8021-4 atcaccgcag gaattccaga aatggcaa-- gtatcaccta ttaatactac attgagagga aaacatcacc tttattgagc aaaatctgta tttaattatg
```

```
8021-1 atcaccgcag gaattccaga aatggcaa-- gtatcgccta ttaatactac attgagagga aaacatcacc tttattgagc aaaatctgta tttaattatg
8021-3 atcaccgcag gaattccaga aatggcaa-- gtatcgccta ttaatactac attgagagga aaacatcacc cttattgagc aaaatctgta tttaattatg
8021-5 atcaccgcag gaattccaga aatggcaa-- gtatcgccta ttaatactac attgagagga aaacatcacc tttgttgagc aaaatctgta tttaattatg
8021-0 atcacggcaa gagatccata aacg------ ---------- ---------- ---------- ---------- ---------- ---------- ----------

       6666666666 6666666666 6666666666 6666666666 6666666666 66666666
       0000000001 1111111112 2222222223 3333333334 4444444445 55555555
       1234567890 1234567890 1234567890 1234567890 1234567890 12345678
8021-2 tgtgtgatta tttttaggtc acgagattgt gcagtccgaa caggattagt gggtaaca
8021-4 tgtgtgatta tttttaggtc acgagattgt gcagtccgaa caggattagt gggtaaca
8021-1 tgtgtgatta tttttaggtc acgagattgt gcagtccgaa caggattagt gggtaaca
8021-3 tgtgtgatta tttttaggtc acgagattgt gcagtccgaa caggattagt gggtaaca
8021-5 tgtgtgatta tttttaggtc acgagattgt gcagtccgaa caggattagt gggtaaca
8021-0 ---------- -------gtc acgagattgt gcagtccgaa caggattagt gggtaaca
```

SNP LOCATIONS: 50T/C, 75T/C, 184C/A, 103A/G, 201C/T, 426T/C, 536G/A

39.

```
                1 1111111112 2222222223 3333333334 4444444445 5555555556 6666666667 7777777778 8888888889 9999999990
       1234567890 1234567890 1234567890 1234567890 1234567890 1234567890 1234567890 1234567890 1234567890 1234567890
8025-4 caggaacttg tccgtgtgtc tgtgagttac actttggatt tgcgtctctt tgtttctgct tgttttccat aatgctacat tcaattctgg ctcatctttc
8025-5 caggaacttg tccgtgtgtc tgtgagttac actttggatt tgcgtctctt tgtttctgct tgttttccat aatgctacat tcaattctgg ctcatctttc
8025-1 caggaacttg tccgtgtgtc tgtgagttac actttggatt tgcgtctctt tgtttctgct tgttttccat aatgctacat tcaattctgg ctcatctttc
8025-3 caggaacttg tccgtgtgtc tgtgagttac actttggatt tgcgtctctt tgtttctgct tgttttccat aatgctacaa tcaattctgg ctcattgctc
8025-2 caggaacttg tccgtgtgtc tgtgagttac actttggatt tgcgtctctt tgtttctgct tgttttccat aatgctacat tcaattctgg ctcatctttc

       1111111111 1111111111 1111111111 1111111111 1111111111 1111111111 1111111111 1111111111 1111111111 1111111112
       0000000001 1111111112 2222222223 3333333334 4444444445 5555555556 6666666667 7777777778 8888888889 9999999990
       1234567890 1234567890 1234567890 1234567890 1234567890 1234567890 1234567890 1234567890 1234567890 1234567890
8025-4 tttttcccct catactagag cctgattaag gatcctaagc tgtcaagtat cgtgatgaat cctcatatca agcgcagtct caaacagaag actttcattg
```

8025-5 tttttcccct catactagag cctgattaag gatcctaagc tgtcaagtat cgtgatgaat cctcatatca agcgcagtct caaacagaag actttcattg
8025-1 tttttcccca catactagag cctgattaag gatcctaagc tgtcaagtat tgtgatgaat cctcatatca agcgcagtct caaacagaag actttcattg
8025-3 tttttcccct catactagag cctgattaag gatcctaagc tgtcaagtat tgtgatgaat cctcatatca agcgcagtct caaacagaag actttcattg
8025-2 tttttcccct catactagag cctgattaag gatcctaagc tgtcaagtat tgtgatgaat cctcatatca agcgcagtct caaacagaag actttcattg

2222222222 2222222222 2222222222 2222222222 2222222222 2222222222 2222222222 2222222222 2222222222 2222222223
0000000001 1111111112 2222222223 3333333334 4444444445 5555555556 6666666667 7777777778 8888888889 9999999990
1234567890 1234567890 1234567890 1234567890 1234567890 1234567890 1234567890 1234567890 1234567890 1234567890
8025-4 atgctttgac taaggcgaaa ctctccccca tcaccatcaa cctcatcagt gagttttcta accagccttt attttcacta tcactcaggt tcggtggcgg
8025-5 atgctttgac taaggcgaaa ctctccccca tcaccatcaa cctcatcagt gagttttcta accagccttt attttcacta tcactcaggt tcggtggcgg
8025-1 atgctttgac taaggcgaaa ctctccccca tcaccatcaa cctcatcagt gagttttcta accagccttt attttcacta tcactcaggc tcggtggcgg
8025-3 atgctttgac taaggcgaaa ctctccccca tcaccatcaa cctcatcagt gagttttcta accagccttt attttcacta tcactcaggt tcagtagctg
8025-2 atgctttgac taaggcgaaa ctctccccca tcaccatcaa cctcatcagt gagttttcta accagccttt attttcacta tcactcaggt tcagtagctg

3333333333 3333333333 3333333333 3333333333 3333333333 3333333333 3333333333 3333333333 3333333333 3333333334
0000000001 1111111112 2222222223 3333333334 4444444445 5555555556 6666666667 7777777778 8888888889 9999999990
1234567890 1234567890 1234567890 1234567890 1234567890 1234567890 1234567890 1234567890 1234567890 1234567890
8025-4 tttttggcac gctcgaagcg ggtctcgccc agggtatgat tcagtgtaga accgccactg ctcaggttga ttcaatagaa ctatgatgtg attaaaaaat
8025-5 tttttggcac gctcgaagcg ggtctcgccc agggtatgat tcagtgtaga accgccactg ctcaggttga ttcaatagaa ctatgatgtg attaaaaaac
8025-1 tttttggcac gctcgaagcg ggtctcgccc agggtatgat tcagtgtaga acggccactg ctcaggttga ttcaatagaa ctatgatgtg attaaaaaat
8025-3 tttttggcac gctcgaagcg ggtctcgccc agggtgtaat tcagtgtaga accgccactg ctcaggttga ttcaatagaa ctatgatgtg attaaaaaat
8025-2 tttttggcac gctcgaagcg ggtctcgccc agggtgtaat tcagtgtaga accgccactg ctcaggttga ttcaatagaa ctatgatgtg attaaaaaat

4444444444 4444444444 4444444444 4444444444 4444444444 4444444444 4444444444 4444444444 4444444444 4444444445
0000000001 1111111112 2222222223 3333333334 4444444445 5555555556 6666666667 7777777778 8888888889 9999999990
1234567890 1234567890 1234567890 1234567890 1234567890 1234567890 1234567890 1234567890 1234567890 1234567890
8025-4 gtttgcacac attaagtgaa aagtgtagtg tagaagagcg actttgacat tgaaaaacat tgaaattcat gatagttgca tttagaaaat taggagtaat
8025-5 gtttgcacac attaagtgaa aagtgtagtg tagaagagcg actttgacat tgaaaaacat tgaaattcat gatagttgca tttagaaaat taggagtaat
8025-1 gtttgcacac attaagtgaa aagtgtagtg tagaagagcg actttgacat tgaaaaacat tgaaattcat gatagttgca tttagaaaat taggagtaat
8025-3 gtttgcacac attaagtaaa aagtgtagtg ttgaagagcg actttgacat tgaaaaacat tgaaattcat gatagttgca tttagaaaat taggagtaat

```
8025-2 gtttgcacat attaagtgaa aagtgtagtg tcgaagagcg actttgacat tgaaaaacat tgaaattcat gatagttgca tttagaaaat caggagtaat

       5555555555 5555555555 5555555555 5555555555 5555555555 5555555555 5555555555 5555555555 5555555555 5555555556
       0000000001 1111111112 2222222223 3333333334 4444444445 5555555556 6666666667 7777777778 8888888889 9999999990
       1234567890 1234567890 1234567890 1234567890 1234567890 1234567890 1234567890 1234567890 1234567890 1234567890
8025-4 ttataggttt gaaagtgcat tatctatgaa tatttacaat tatttttgtt tacagatgtc ctagcagaaa atggccgctt gaccttgacc catgatgtca
8025-5 ttataggttt gaaagtgcat tatctatgaa tatttacaat tatttttgtt tacagatgtc ctagcagaaa atggccgctt gaccttgacc catgatgtca
8025-1 ttataggttt gaaagtgcat tatctatgaa tatttacaat tatttttgtt tacagatgtc ctagcagaaa atggccgctt gaccttgacc catgatgtca
8025-3 ttataggttt gaaagtgcat tatctatgaa tatttacaat tatttttgtt tacagatgtc ctagcagaaa atggccgctt gaccttgacc catgatgtca
8025-2 ttataggttt gaaagtgcat tatctacgaa tatttacaat tatttttgtt tacagatgtc ctagcagaaa atggccgctt gaccttgacc cctgatgtca

       6666666666 6666666666 6666666666 6666666666 6666666666 6666666666 6666666666 6666666666 6666666666 6666666667
       0000000001 1111111112 2222222223 3333333334 4444444445 5555555556 6666666667 7777777778 8888888889 9999999990
       1234567890 1234567890 1234567890 1234567890 1234567890 1234567890 1234567890 1234567890 1234567890 1234567890
8025-4 tcacagcctt tggcaaaatg atgagtgccc acagaggaga ggtcacatgc tcagtcacca ccgctcaggt aagcggtgta tacattcatg cacaaatata
8025-5 tcacagcctt tggcaaaatg atgagtgccc acagaggaga ggtcacatgc tcagtcacca ccgctcaggt aagcggtgta tacattcatg cacaaatata
8025-1 tcacagcctt tggcaaaatg atgagtgccc acagaggaga ggtcacatgc tcagtcacca ccgctcaggt aagcggtgta tacat-catg cacaaatata
8025-3 tcacagcctt tggcaaaatg atgagtgccc acagaggaga ggtcacatgc tcagtcacca ccgctcaggt aagcggtgta tacattcatg cacaaatata
8025-2 tcacagcctt tggcaaaatg atgagtgccc acagaggaga ggtcacatgc tcagtcacca ccgctcaggt aagcggtgta tacattcatg cacaaatatg

       7777777777 7777777777 7777777777 7777777777 7777777777 7777777777 7777777777 7777777777 7777777777 7777777778
       0000000001 1111111112 2222222223 3333333334 4444444445 5555555556 6666666667 7777777778 8888888889 9999999990
       1234567890 1234567890 1234567890 1234567890 1234567890 1234567890 1234567890 1234567890 1234567890 1234567890
8025-4 ttcgcatgtt aatatcacta ctaaagtaaa taatttgtat tcaaatattt tttggttgcg taaaactttt tgaatttgtt gtt-cattaa agttttgaat
8025-5 ttcgcatgtt aatatcacta ctaaagtaaa taatttgtat tcaaatattt tttggttgcg taaaactttt tgaatttgtt gtt-cattaa agttttgaat
8025-1 ttcgcatgtt aatatcacta ctaaagtaaa tcatttgtat tcaaatattt tttggttgcg taaaactttt tgaatttgtt gtt-cattaa agttttgaat
8025-3 ttcgcatgtt aatatcatta ctaaagtaaa tcatttgtat tcaaatattt tttggttgcg taaaactttt tgaatttgtt gtt-cattaa agttttgaat
8025-2 ttcgcatgtt aatatcacta ctaaagtaaa taatttgtat ttaaatattt tttggtcgca taaaactttt agaatttact gtttctttaa agttttgaat

       8888888888 8888888888 8888888888 8888888888 8888888888 8888888888 8888888888 8888888
```

```
        0000000001 1111111112 2222222223 3333333334 4444444445 5555555556 6666666667 7777777
        1234567890 1234567890 1234567890 1234567890 1234567890 1234567890 1234567890 1234567
8025-4 ggaacctttg tgtctgtgaa cagcctctgg atgaagctaa tcttgcagag ctgaatgtgg cactgaatgg tttcctg
8025-5 ggaacctttg tgtctgtgaa cagcctctgg atgaagctaa tcttgcagag ctgaaggtgg cactgaatgg tttcctg
8025-1 ggaacctttg tgtctgtgaa cagcctctgg atgaagctaa tcttgcagag ctgaaggtgg cactgaatgg tttcctg
8025-3 ggaacctttg tgtctgtgaa cagcctctgg atgaagctaa tcttgcagag ctgaaggtgg cactgaatgg tttcctg
8025-2 gaaacctttg tgtctgtgaa cagcctctgg atgaagctaa tcttgcagag ctgaaggtgg cactgaatgg tttcctg
```

SNP LOCATIONS: 151T/C, 293G/A, 296G/A, 299G/T, 336A/G, 338G/A, 732A/C

40.

```
                 1 1111111112 2222222223 3333333334 4444444445 5555555556 6666666667 7777777778 8888888889 9999999990
        1234567890 1234567890 1234567890 1234567890 1234567890 1234567890 1234567890 1234567890 1234567890 1234567890
8034-0 ctgtgtttgg tgggtttcgt ctggaaaatg ctgtagtaaa acccaaattc gttcaaaatg gctgctgctc atcgtttagt gattgtacgc cacggcgaga
8034-1 ctgtgtttgg tgggtttcgt ctggaaaata ctgtagtaaa acccaaattc gttcaaaatg gctgctgctc atcgtttagt gattgtacgc cacggcgaga
8034-3 ctgtgtttgg tgggtttcgt ctggaaaata ctgtagtaaa acccaaattc gttcaaaatg gctgctgctc atcgtttagt gattgtacgc cacggcgaga
8034-6 ctgtgtttgg tgggtttcgt ctggaaaata ctgtagtaaa acccaaattc gttcaaaatg gctgctgctc atcgtttagt gattgtacgc cacggagaga
8034-7 ctgtgtttgg tgggtttcgt ctggaaaata ctgtagtaaa acccaaattc gttcaaaatg gctgctgctc atcgtttagt gattgtacgc cacggagaga
8034-4 ctgtgtttgg tgggtttcgt ctggaaaata ctgtagtaaa acccaaattc gttcaaaatg gctgctgctc atcgtttagt gattgtacgc cacggcgaga
8034-2 ctgtgtttgg tgggtttcgt ctggaaaata ctgtagtaaa acccaaattc gttcaaaatg gctgctgctc atcgtttagt gattgtacgc cacggcgaga
8034-5 ctgtgtttgg tgggtttcgt ctggaaaata ctgtagtaaa acccaaattc gttcaaaatg gctgctgctc atcgtttagt gattgtacgc cacggcgaga

        1111111111 1111111111 1111111111 1111111111 1111111111 1111111111 1111111111 1111111111 1111111111 1111111112
        0000000001 1111111112 2222222223 3333333334 4444444445 5555555556 6666666667 7777777778 8888888889 9999999990
        1234567890 1234567890 1234567890 1234567890 1234567890 1234567890 1234567890 1234567890 1234567890 1234567890
8034-0 gctcctggaa ccaagagaac cgtttctgtg gctggtttga tgcagacctc agtgaaaagg gtctggagga agcaaagcga ggtgctcaag ccatcaaaga
8034-1 gctcctggaa ccaagagaac cgtttctgtg gctggtttga tgcagacctc agtgaaaagg gtctggagga agcaaagcga ggtgctcaag ccatcaaaga
8034-3 gctcctggaa ccaagagaac cgtttctgtg gctggtttga tgcagacctc agtgaaaagg gtccggagga agcaaagcga ggtgctcaag ccatcaaaga
8034-6 gctcctggaa ccaagagaac cgtttctgtg gctggtttga tgcagacctc agtgaaaagg gtctggagga agcaaagcga ggtgctcaag ccatcaaaga
8034-7 gctcctggaa ccaagagaac cgtttctgtg gctggtttga tgcagacctc agtgaaaagg gtctggagga agcaaagcga ggtgctcaag ccatcaaaga
```

```
8034-4 gctcctggaa ccaagagaac cgtttctgtg gctggtttga tgcagacctc agtgaaaagg gtctggagga agcaaagcga ggtgctcaag ccatcaaaga
8034-2 gctcctggaa ccaagagaac cgtttctgtg gctggtttga tgcagacctc agtgaaaaag gtctggagga agcaaagcga ggtgctcaag ccatcaaaga
8034-5 gctcctggaa ccaagaggac cgtttctgtg gctggtttga tgcagacctc agtgaaaaag gtctggagga agcaaagcga ggtgctcaag ccatcaaaga

       2222222222 2222222222 2222222222 2222222222 2222222222 2222222222 2222222222 2222222222 2222222222 2222222223
       0000000001 1111111112 2222222223 3333333334 4444444445 5555555556 6666666667 7777777778 8888888889 9999999990
       1234567890 1234567890 1234567890 1234567890 1234567890 1234567890 1234567890 1234567890 1234567890 1234567890
8034-0 tgcaggcatg aagtttgatg tgtgctacac ttccgttttg aagcgcgcta tcaagactct gtggaccatc atggaggtca cagaccagat gtggctgcct
8034-1 tgcaggcatg aagtttgatg tgtgctacac ttccgttttg aagcgcgcta tcgagactct gtggaccatc atggaggtca cagaccagat gtggctgcct
8034-3 tgcaggcatg aagtttgatg tgtgctacac ttccgttttg aagcgcgcta tcaagactct gtggaccatc atggaggtca cagaccagat gtggctgcct
8034-6 tgcaggcatg aagtttgatg tgtgctacac ttccgttttg aagcgcgcta tcaagactct gtggaccatc atggaggtca cagaccagat gtggctgcct
8034-7 tgcaggcatg aagtttgatg tgtgctacac ttccgttttg aagcgcgcta tcaagactct gtggaccatc atggaggtca cagaccagat gtggctgcct
8034-4 tgcaggcatg aagtttgatg tgtgttacac ttccgttttg aagcgcgcta tcaagactct gtggaccatc atggaggtca cagaccagat gtggctgcct
8034-2 tgcaggcatg aagtttgatg tgtgctacac ttccgttttg aagcgcgcta tcaagactct gtggaccatc atggaggtca cagaccagat gtggctgcct
8034-5 tgcaggcatg aagtttgatg tgtgctacac ttccgttttg aagcgcgcta tcaagactct gtggaccatc atggaggtca cagcccagat gtggctgcct

       3333333333 3333333333 3333333
       0000000001 1111111112 2222222
       1234567890 1234567890 1234567
8034-0 gtagtgcgca cctggcgtct gaatgaa
8034-1 gtagtgcgca cctggcgtct gaatgaa
8034-3 gtagtgcgca cctggcgtct gaatgaa
8034-6 gtagtgcgca cctggcgtct gaatgaa
8034-7 gtagtgcgca cctggcgtct gaatgaa
8034-4 gtagtgcgca cctggcgtct gaatgaa
8034-2 gtagtgcgca cctggcgtct gaatgaa
8034-5 gtagtgcgca cctggcgtct gaatgaa
```

SNP LOCATIONS: 96C/A, 159G/A

41.

```
                 1 1111111112 2222222223 3333333334 4444444445 5555555556 6666666667 7777777778 8888888889 9999999990
        1234567890 1234567890 1234567890 1234567890 1234567890 1234567890 1234567890 1234567890 1234567890 1234567890
8041-3 ctttccttct acaaaccgtc tccaaaacgc tataagattc ttctagaaga caaggccttt ccatcagacc acgtatgtat gcctaaatta atcctaacat
8041-6 ctttccttct acaaaccgtc tccaaaacgc tataagattc ttctagaaga caaggccttt ccatcagacc acgtatgtat gcctaaatta atcctaacat
8041-2 ctttccttct acaaaccgtc tccaaaacgc tataagattc ttctagaaga caaggccttt ccatcagacc acgtatgtat gcctaaatta atcctaacat
8041-5 ctttccttct acaaaccgtc tccaaaacgc tataagattc ttctagaaga caaggccttt ccatcagacc acgtatgtat gcctaaatta atcctaacat
8041-1 ctttccttct acaaaccgtc tccaaaacgc tataagattc ttctagaaga caaggccttt ccatcagacc acgtatgtat gcctaaatta atcctaacat
8041-7 ctttccttct acaaaccgtc tccaaaacgc tataagattc ttctagaaga caaggccttt ccatcagacc acgtatgtat gcctaaatta atcctaacat
8041-4 ctttccttct acaaaccgtc tccaaaacgc tataagattc ttctagaaga caaggccttt ccatcagacc acgtatgtat gcctaaatta atcctaacat

       1111111111 1111111111 1111111111 1111111111 1111111111 1111111111 1111111111 1111111111 1111111111 1111111112
       0000000001 1111111112 2222222223 3333333334 4444444445 5555555556 6666666667 7777777778 8888888889 9999999990
       1234567890 1234567890 1234567890 1234567890 1234567890 1234567890 1234567890 1234567890 1234567890 1234567890
8041-3 tattttgatg tgaacattac gggataagtc atgtttgtgt gtggatgtgc agtacgccat tgaatctcag atccggctga gaggactgga cgtgaccgac
8041-6 tattttgatg tgaacattac gggataagtc atgtttgtgt gtggatgtgc agtacgccat tgaatctcag atccggctga gaggactgga cgtgaccgac
8041-2 tattttgatg tgaacattac gggataagtc atgtttgtgt gtggatgtgc agtatgccat tgaatctcag atccggctga gaggactgga cgtgaccgac
8041-5 tattttgatg tgaacattac gggataagtc atgtttgtgt gtggatgtgc agtatgccat tgaatctcag atccggctga gaggactgga cgtgaccgac
8041-1 tattttgatg tgaacattac gggataagtg atgtttgtgt gtggatgtgc agtatgccat tgaatctcag atccggctga gaggactgga cgtgaccgac
8041-7 tattt-gatg tgaacattac gggataagtg atgtttgtgt gtggatgtgc agtatgccat tgaatctcag atccggctga gaggactgga cgtgaccgac
8041-4 tattttgatg tgaacattac gggataagtg atgtttgtgt gtggatgtgc agtatgccat tgaatctcag atccagctga gaggactgga cgtgaccgac

       2222222222 2222222222 2222222222 2222222222 2222222222 2222222222 2222222222 2222222222 2222222222 2222222223
       0000000001 1111111112 2222222223 3333333334 4444444445 5555555556 6666666667 7777777778 8888888889 9999999990
       1234567890 1234567890 1234567890 1234567890 1234567890 1234567890 1234567890 1234567890 1234567890 1234567890
8041-3 agcatgctga tcattaaacc aagacaggta atgacatcac ttcctctcag tgtgggcagt atgacaggaa gtgctctgtg agcgtgtgga ttggccgttt
8041-6 agcatgctga tcattaaacc aagacaggta atgacatcac ttcctctcag tgtgggcagt atgacaggaa gtgctctgtg agcgtgtgga ttggccgttt
8041-2 agcatgctga tcattaaacc aagacaggta atgacatcac ttcctctcag tgtgggcagt atgacaggaa gtgctctgtg agcgtgtgga ttggccgttt
8041-5 agcatgctga tcattaaacc aagacaggta atgacatcac ttcctctcag tgtgggcagt atgacaggaa gtgctctgtg agcgtgtgga ttggccgttt
8041-1 agcatgctga tcattaaacc aagacaggta atgacatcac ttcctctcag tgtgggcagt atgacaggaa gtgctctgtg agcgtgtgga ttggccgttt
```

```
8041-7 agcatgctga tcattaaacc aagacaggta atgacatcac ttcctctcag tgtgggcagt atgacaggaa gtgctctgtg agcgtgtgga ttggccgttt
8041-4 agcatgctga tcattaaacc aagacaggta atgacatcac ttcctctcag tgtgggcagt atgacaggaa gtgctctgtg agcgtgtgga ttggccgttt

       3333333333 3333333333 333333333
       0000000001 1111111112 222222222
       1234567890 1234567890 123456789
8041-3 cagggtgaag acaccatcag gacagagga
8041-6 cagggtgaag acaccatcag gacagagga
8041-2 cagggtgaag acaccatcag gacagagga
8041-5 cagggtgaag acaccatcag gacagagga
8041-1 cagggtgaag acaccatcag gacagagga
8041-7 cagggtgaag acaccatcag gacagagga
8041-4 cagggtgaag acaccatcag gacagagga
```

SNP LOCATIONS: 130C/G, 155T/C

42.

```
                1 1111111112 2222222223 3333333334 4444444445 5555555556 6666666667 7777777778 8888888889 9999999990
       1234567890 1234567890 1234567890 1234567890 1234567890 1234567890 1234567890 1234567890 1234567890 1234567890
8048-2 taggcagtga atgtgggatt gaatttgatg aggagaaaac tgctgtcatt gaccaccata actatgacgt ttctgatcct ggtgagagca caccctgatt
8048-3 taggcagtga atgtgggatt gaatttgatg aggagaaaac tgctgtcatt gaccaccata actatgacgt ttctgatcct ggtgagagca caccctgatt
8048-1 taggcagtga atgtgggatt gaatttgatg aggagaaaac tgctgtcatt gaccaccata actatgacgt ttctgatcct ggtgagagca caccctgatt
8048-4 taggcagtga atgtgggatt gaatttgatg aggagaaaac tgctgtcatt gaccaccata actatgacgt ttctgatcct ggtgagagca caccctgatt
8048-0 taggcagtga atgtgggatt gaatttgatg aggagaaaac tgctgtcatt gaccaccata actatgacgt ttctgatcct ggtgag--ca caccctgatt

       1111111111 1111111111 1111111111 1111111111 1111111111 1111111111 1111111111 1111111111 1111111111 1111111112
       0000000001 1111111112 2222222223 3333333334 4444444445 5555555556 6666666667 7777777778 8888888889 9999999990
       1234567890 1234567890 1234567890 1234567890 1234567890 1234567890 1234567890 1234567890 1234567890 1234567890
8048-2 gttgcagatc cagagaatct tcttaaagcc ccaacaatag ttggcaagcc cactaataaa ccagtcctgt tcaaaggtgt tggcatggtg gctgatccag
8048-3 gttgcagatc cagagaatct tcttaaagcc ccaacaatag ttggcaagcc cactaataaa ccagtactgt tcaaaggtgt tggcatggtg gctgatccag
```

```
8048-1 gttgcagatc cagagaatct tcttaaagcc ccaacaatag ttggcaagcc cactaataaa ccagtcctgt tcaaaggtgt tggcatggtg gctgatccag
8048-4 gttgcagatc cagagaatct tcttaaagcc ccaactatag ttggcaagcc cactaataaa ccagtcctgt tcaaaggtgt tggcatggtg gctgatccag
8048-0 gttgcagacc cagagaatct tcttaaatcc ccaactatag ttggcaagcc cactgataaa ccagtcctgt tcaaaggtgt tggcatggtg gctgatccag

       2222222222 2222222222 2222222222 2222222222 2222222222 22222222
       0000000001 1111111112 2222222223 3333333334 4444444445 55555555
       1234567890 1234567890 1234567890 1234567890 1234567890 12345678
8048-2 ataactctct ggtgctggac attctgacag gatcctccac ctcttactcc tacttccc
8048-3 ataactctct ggtgctggac attctgacag gatcctccac ctcttactcc tacttccc
8048-1 ataactctct ggtgctggac attctgacag gatcctccac ctcttactcc tacttccc
8048-4 ataactctct ggtgctggac attctgacag gatcctccac ctcttactcc tacttccc
8048-0 ataactctct ggtgctggac attctgacag gatcctccac ctcttactcc tacttccc
```

SNP LOCATIONS: 136A/T

43.

```
                   1 1111111112 2222222223 3333333334 4444444445 5555555556 6666666667 7777777778 8888888889 9999999990
         1234567890 1234567890 1234567890 1234567890 1234567890 1234567890 1234567890 1234567890 1234567890 1234567890
8050-1-3 tcaaggaagg caacgggaag tggc-accga cggatttatt cgttgaccaa agtctgcaat cggacattta tgagtgcagt ttttcatcgc agttgagatc
8050-1-7 tcaaggaagg caacgggaag tggccaccga cggatttatt cgttgaccaa agtctgcaat cggacattta tgagtgcagt ttttcatcgc agttgagatc
8050-1-5 tcaaggaagg caacgggaag tggccaccga cggatttatt cgttgaccaa agtctgcaat cggacattta tgagtgcagt ttttcatcgc agttgagatc
8050-1-2 tcaaggaagg caacgggaag tggccaccga cggatttatt cgttgaccaa agtctgcaat cggacattta tgagtgcagt ttttcatcgc agttgagatc
8050-1-0 tcaaggaagg caacgggaag tggccaccga cggacttatt cgttgaccaa agtctgcaat cggacattta tgactgcagt tcttcatcgc agttgagacc
8050-1-6 tcaaggaagg caacgggaag tggccaccga cggacttatt cgttgaccaa agtctgcaat cggacagtta tgactgcagt tcttcatcgc agttgagacc
8050-1-1 tcaaggaagg caacgggaag cggccaccga cggacttatt cgttgaccaa agtctgcaat cggacattta tgactgcagt tcttcatcgc agttgagacc
8050-1-4 tcaaggaagg caacgggaag tggccaccga cggatttatt cgttgaccaa agtctgcaat cggacattta tgactgcagt tcttcatcgc agttgagacc

         1111111111 1111111111 1111111111 1111111111 1111111111 1111111111 1111111111 111111111
         0000000001 1111111112 2222222223 3333333334 4444444445 5555555556 6666666667 777777777
         1234567890 1234567890 1234567890 1234567890 1234567890 1234567890 1234567890 123456789
```

```
8050-1-3 cccc-tcaaa caggctcatt tcccgcccgc tggagcgagt tttgtcgctg tggcagtgga ctcggtactt ctcccgctg
8050-1-7 cccc-tcaaa caggctcatt tcccgcccgc tggagcgagt tttgtcgctg tggcagtgga ctcggtactt ctcccgctg
8050-1-5 cccc-tcaaa caggctcatt tcccgcccgc tggagcgagt tttgtcgctg tggcagtgga ctcggtactt ctcccgctg
8050-1-2 cccc-tcaaa caggctcatt tcccgcccgc tggagcgagt tttgtcgctg tggcagtgga ctcggtactt ctcccgctg
8050-1-0 ccccctcaaa caggctcatt tcccgcccac tggagcgagt tttgtcgctg tggaagtgga ctcggtactt ctcccgctg
8050-1-6 ccccctcaaa caggctcatt tcccgcccac tggagcgagt tttgtcgctg tggaagtgga ctcggtactt ctcccgctg
8050-1-1 ccccctcaaa caggctcatt tcccgcccac tggagcgagt tttgtcgctg tggcagtgga ctcggtactt ctcccgctg
8050-1-4 ccccctcaaa caggctcatt tcccgcccac tggagcgagt tttgtcgctg tggaagtgga ctcggtactt ctcccgctg
```

SNP LOCATIONS: 35T/C, 74C/G, 82C/T, 99C/T, 129A/G, 154C/A

44.

```
                    1 1111111112 2222222223 3333333334 4444444445 5555555556 6666666667 7777777778 8888888889 9999999990
           1234567890 1234567890 1234567890 1234567890 1234567890 1234567890 1234567890 1234567890 1234567890 1234567890
8052cg2-5 ---------a ccctcattgt ggtgtccaat ccaggtgagc aaacactctc acaacatgct taattttta atgaattgct ta-gttttgc tgtaccgcac
8052cg2-6 accctcat-a ccctcattgt ggtgtccaat ccaggtgagc aaacattctc acaacatgct taattttta atgaattgct ta-gttttgc tgtaccgcac
8052cg2-1 ---------a ccctcattgt ggtgtccaat ccaggtgagc aaacactctc acaacatgct taattttta atgaattgct ta-gttttgc tgtaccgcac
8052cg2-4 ---------a ccctcattgt ggtgtccaat ccaggtgagc aaacactctc acaacatgct taattttta atgaattgct tc-gttttgc tgtaccgcac
8052cg2-2 ---------a ccctcattgt ggtgtccaat ccaggtgagc aaaca----- --aacacact cacacatcat gcgtaacgtt aatgttttgc tgtgcagcac
8052cg2-3 ---------a ccctcattgt ggtgtccaat ccaggtgagc aaaca----- --aacacact cacacatcat gcgtaacgtt aatgttttgc tgtgcagcac

          1111111111 1111111111 1111111111 1111111111 1111111111 1111111111 1111111111 1111111111 1111111111 1111111112
          0000000001 1111111112 2222222223 3333333334 4444444445 5555555556 6666666667 7777777778 8888888889 9999999990
          1234567890 1234567890 1234567890 1234567890 1234567890 1234567890 1234567890 1234567890 1234567890 1234567890
8052cg2-5 cacaaccaat gtgcataaat gaaattatcc aaacaaatgc agcaaattct gttaaaataa tatttttaaa ataaaatttg agttaatttc tagaaattta
8052cg2-6 cacaaccaat gtgcataaat gaaattatcc aaacaaatgc agcaaattct gttaaaataa tatttttaaa ataaaatttg agttaatttc tagaaattta
8052cg2-1 cacaaccaat gtgcataaat gaaattatcc aaacaaatgc agcaaattct gttaaaataa tatttttaaa ataaaatttg agttaatttc tagaaattta
8052cg2-4 cacaaccaat gtgcataaat gaaattatcc aaacaaatgc agcaaattct gttaaaataa tatttttgaa ataaaatttg agttaatttc tagaaattta
8052cg2-2 gacaaccaac ------aaac caaattacat gttaaaattc tataaatttt actttt-tac ttttttaat at-------- --ttattctt taa-----ta
8052cg2-3 gacaaccaac ------aaac caaattacat gttaaaattc tataaatttt actttt-tac ttttttaat at-------- --ttattctt taa-----ta
```

```
           2222222222 2222222222 2222222222 2222222222 2222222222 2222222222 2222222222 2222222222 2222222222 2222222223
           0000000001 1111111112 2222222223 3333333334 4444444445 5555555556 6666666667 7777777778 8888888889 9999999990
           1234567890 1234567890 1234567890 1234567890 1234567890 1234567890 1234567890 1234567890 1234567890 1234567890
8052cg2-5  atgatctgtt ttatttccta gttattggca aataatgtct cggccaaaga tttattgtgc agcttctaat gaga--gaaa aaacgaaat- ------ggca
8052cg2-6  atgatctgtt ttatttccta gttattggca aataatgtct cggccaaaga tttattgtgc agcttctaat gaga--gaaa aaacgaaat- ------ggca
8052cg2-1  atgatctgtt ttatttccta gttattggca aataatgtct cggccaaaga tttattgtgc agcttctaat gaga--gaaa aaacgaaat- ------ggca
8052cg2-4  atgatctgtt ttatttccta gttattggca aataatgtct cggccaaaga tttattgtgc agcttctaat gaga--gaaa aaacgaaat- ------ggca
8052cg2-2  ccagtatatg t--tttcata gttaatggca aataatatat tagccgcaga tata--gtgc agctcctaac gagatcgaga atacaaaatt ctaaatgcta
8052cg2-3  ccagtatatg t--tttcata gttaatggca aataatatat tagccgcaga tata--gtgc agctcctaac gagatcgaga atacaaaatt ctaaatgcta

           3333333333 3333333333 3333333333 3333333333 3333333333 3333333333 3333333333 3333333333 3333333333 3333333334
           0000000001 1111111112 2222222223 3333333334 4444444445 5555555556 6666666667 7777777778 8888888889 9999999990
           1234567890 1234567890 1234567890 1234567890 1234567890 1234567890 1234567890 1234567890 1234567890 1234567890
8052cg2-5  ttctaaatct gtgtctattt caggttcctg tagtgtatta atagtaactg tggttgtgtc tgtgttcata cagtggatgt gttgacctac gtgacctgga
8052cg2-6  ttctaaatct gtgtccattt caggttcctg tagtgtatta atagtaactg tggttgtgtc tgtgttcata cagtggatgt gttgacctac gtgacctgga
8052cg2-1  ttctaaatct gtgtccattt caggttcctg tagtgtatta atagtaacag tggttgtgtc tgtgttcata cagtggatgt gttgacctac gtgacctgga
8052cg2-4  ttctaaatct gtgtccattt caggttcctg tagtgtatta atagtaacag tggttgtgtc tgtgttcata cagtggatgt gttgacctac gtgacctgga
8052cg2-2  ttctaaagct atgtccattt cagactcctg cactgtattt acagtaattg cggttg---- tctgttcata cagtggatgt tttgacctac gtgacatgga
8052cg2-3  ttctaaagct atgtccattt cagactcctg cactgtattt acagtaattg cggttg---- tctgttcata cagtggatgt tttgacctac gtgacatgga

           4444444444 4444444444 4444444444 4444444444 4444444444 4444444444 4444444444 4444444444 4444444444 4444444445
           0000000001 1111111112 2222222223 3333333334 4444444445 5555555556 6666666667 7777777778 8888888889 9999999990
           1234567890 1234567890 1234567890 1234567890 1234567890 1234567890 1234567890 1234567890 1234567890 1234567890
8052cg2-5  agctgagcgg cctgccaaag caccgtgtca ttggcagcgg gaccaacctg gactctgctc gcttccgcca catcatggcc gagaaactgg gcatccactc
8052cg2-6  agctgagcgg cctgccaaag caccgtgtca tcggcagcgg gaccaacctg gactctgctc gcttccgcca catcatggcc gagaaatggg gcatccactc
8052cg2-1  agctgagcgg cctgccaaag caccgtgtca tcggcagcgg gaccaacctg gactctgctc gcttccgcca catcatggcc gagaaactgg gcatccactc
8052cg2-4  agctgagcgg cctgccaaag caccgtgtca tcggcagcgg gaccaacctg gactctgctc gcttccgcca catcatggcc gagaaactgg gcatccactc
8052cg2-2  agctgagcgg cctgccaaag caccgtgtca tcggcagcgg gaccaacctg gactccgctc gtttccgcta catcatggct gagaaactag gcatccactc
8052cg2-3  agctgagcgg cctgccaaag caccgtgtca tcggcagcgg gaccaacctg gactccgctc gtttccgcta catcatggct gagaaactag gcatccactc
```

```
           5555555555 5555555555 5555555555 5555555555 5555555555 5555555555 5555555555 5555555555 5555555555 5555555556
           0000000001 1111111112 2222222223 3333333334 4444444445 5555555556 6666666667 7777777778 8888888889 9999999990
           1234567890 1234567890 1234567890 1234567890 1234567890 1234567890 1234567890 1234567890 1234567890 1234567890
8052cg2-5  cagcagcttc aacggttata tcctgggaga gcacggagac tccagcggtg agag-aggga gctgaaagaa gagga--aaa tatgagata- --------aa
8052cg2-6  cagcagcttc aacggttata tcctgggaga gcacggagac tccagtggtg agag-aggga gctgaaagaa gagga--aaa tatgagata- --------aa
8052cg2-1  cagcagcttc aacggttata tcctgggaga gcacggagac tccagcggtg agag-aggga gctgaaagaa gagga--aaa tatgagata- --------aa
8052cg2-4  cagcagcttc aacggttata tcctgggaga gcacggagac tccagcggtg agag-aggga gctgaaagaa gagga--aaa tatgagata- --------aa
8052cg2-2  cagcagcttc aacggctaca tcctgggaga gcacggagac tccagcggtg agagcaggaa gaggaaaaca tgagataaga tgtagggtgt cgagagggaa
8052cg2-3  cagcagcttc aacggctaca tcctgggaga gcacggagac tccagcggtg agagcaggaa gaggaaaaca tgagataaga tgtagggtgt cgagagggaa

           6666666666 6666666666 6666666666 6666666666 6666666666 6666666666 6666666666 6666666666 6666666666 6666666667
           0000000001 1111111112 2222222223 3333333334 4444444445 5555555556 6666666667 7777777778 8888888889 9999999990
           1234567890 1234567890 1234567890 1234567890 1234567890 1234567890 1234567890 1234567890 1234567890 1234567890
8052cg2-5  atgggtgt-- ----ggagag gagcaagt-- ----agaacg aatattaaaa tggataaatg tgtactgctt gcatgatgtg acaaggtttc agtcgttgac
8052cg2-6  atgggtgt-- ----ggagag gagcaagtgc tagtagaacg gatattaaaa tggataaatg tgtactgctt gcatgatgtg acacggtttc agtcgttgac
8052cg2-1  atgggtgt-- ----ggagag gagcaagtgc tagtagaacg aatattaaaa cggataaatg tgtactgctt gcatgatgtg acacggtttc agtcgttgac
8052cg2-4  atgggtgt-- ----ggagag g-gcaagtgc tggtagaacg aatattaaaa tggataaatg tgtactgctt gcatgatgtg acacggtttc agtcgttgac
8052cg2-2  atgagtaacc agaggaagag gatgaagggc cagtagaaag aataataaga cggatgaatg tgaactgct- gcatgatgtg acgcgatttc agtcagtcac
8052cg2-3  atgagtaacc agaggaagag gatgaagggc tggtagaaag aataataaga cggatgaatg tgaactgct- gcatgatgtg acgcgacttc agtcagtcac

           7777777777 7777777777 7777777777 7777777777 7777777777 7777777777 7777777777 7777777777 7777777777 7777777778
           0000000001 1111111112 2222222223 3333333334 4444444445 5555555556 6666666667 7777777778 8888888889 9999999990
           1234567890 1234567890 1234567890 1234567890 1234567890 1234567890 1234567890 1234567890 1234567890 1234567890
8052cg2-5  agcatatttg atttttaaaa gtgctgtttt tgtgtttaac agtgcctgtg tggagcggag caaatgttgc tggagtcagc ctgcagaaac tcaaccctga
8052cg2-6  agcatatttg atttttaaaa gtgctgtttt tgtgtttaac agtgcctgtg tggagcggag caaatgttgc tggagtcagc ctgcagaaac tcaaccctga
8052cg2-1  agcatatttg atttttaaaa gtgctgtttt tgtgtttaac agtgcctgtg tggagcggag caaatgttgc tggagtcagc ctgcagaaac tcaaccctga
8052cg2-4  agcatatttg atttttaaaa gtgctgtttt tgtgtttaac agtgcctgtg tggagcggag caaatgttgc tggagtcagc ctgcagaaac tcaaccctga
8052cg2-2  agcgtt---- --------aa gtgctgtttt tgtgtttaac agtgcctgta tggagtggag taaatgtggc tggagtgagc ctgcagaaac tcaaccctga
8052cg2-3  agcgtt---- --------aa gtgctgtttt tgtgtttaac agtgtctgta tggagtggag taaatgtggc tggagtgagc ctgcagaaac tcaaccctga
```

```
          8888888888 8888888888 888888888
          0000000001 1111111112 222222222
          1234567890 1234567890 123456789
8052cg2-5 catcggcaca gataaagaca gcgagaact
8052cg2-6 catcggcaca gataaagaca gcgagaact
8052cg2-1 catcggcaca gataaagaca gcgagaact
8052cg2-4 catcggcaca gataaagaca gcgagaact
8052cg2-2 catcggcaca gataaagaca gcgagaact
8052cg2-3 catcggcaca gataaagaca gcgagaact
```

SNP LOCATIONS: 57T/C, 58G/A, 61T/C, 63A/C, 64T/A, 65T/C, 66T/A, 68T/C, 69T/A, 70A/T, 71A/G, 72T/C, 74A/T, 76T/A, 77T/C, 79C/T, 81T/A, 94A/G, 96C/A, 101C/G, 110T/C, 120T/C, 121G/C, 128T/C, 129C/A, 130C/T, 131A/G, 132A/T, 133A/T, 134C/A, 139G/T, 141A/T, 142G/A, 143C/T, 149C/T, 151G/A, 152T/C, 154A/T, 155A/T, 156A/T, 160A/C, 162A/T, 170A/T, 186A/T, 188T/C, 190C/T, 193G/A, 201A/C, 202T/C, 203G/A, 205C/A, 207G/A, 209T/G, 217C/A, 224T/A, 238C/A, 240C/T, 241G/A, 324G/A, 325T/C, 331T/C, 333G/C, 340A/T, 342T/C, 362G/C, 381G/T, 396C/A, 456T/C, 462G/T, 469C/T, 480C/T, 489G/A, 516T/C, 519T/C, 750G/A, 756C/T, 761C/T, 767T/G, 776C/G

45.

```
                1 1111111112 2222222223 3333333334 4444444445 5555555556 6666666667 7777777778 8888888889 9999999990
       1234567890 1234567890 1234567890 1234567890 1234567890 1234567890 1234567890 1234567890 1234567890 1234567890
8123-4 catt-accac taaa-attag gaggacataa atgagcacta ctactagcct cattaataat tttcctaatt acaattaata tgcttggctt actaccgtat
8123-6 catttaccac taaa-attag gaggacataa atgagcacta ctactagcct cattaataat tttcctaatt acaattaata tgcttggctt actaccgtat
8123-1 catt-accac taaacattag gaggacataa atgagcacta ctactagcct cattaataat tttcctaatt acaattaata tgcttggctt actaccgtat
8123-2 catt-accac taaccattag gaggacataa atgagcacta ctactagcct cattaataat tttcctaatt acaattaata tgcttggctt actaccgtat
8123-3 catt-accac taac-attag gaggacataa atgagcacta ctactagcct cattaataat tttcctaatt acaattaata tgcttggctt actaccgtat
8123-5 catt-accac taaa-attag gaggacataa atgagcacta ctactagcct cattaataat tttcctaatt acaattaata tgcttggctt actaccgtat
8123-7 catt-accac taaaaattag gaggacataa atgagcacta ctactagcct cattaataat tttcctaatt acaattaata tgcttggctt actaccgtat

       1111111111 1111111111 1111111111 1111111111 1111111111 1111111111 1111111111 1111111111 1111111111 1111111112
```

```
       0000000001 1111111112 2222222223 3333333334 4444444445 5555555556 6666666667 7777777778 8888888889 9999999990
       1234567890 1234567890 1234567890 1234567890 1234567890 1234567890 1234567890 1234567890 1234567890 1234567890
8123-4 accttcacac ccacaacaca actatcactc aatatgggat ttgccgtgcc tctatgactc gctactgtaa ttattgggat acgaaatcaa ccaacagttg
8123-6 accttcacac ccacaacaca actatcactc aatatgggat ttgccgtgcc tctatgactc gctactgtaa ttattgggat acgaaatcaa ccaacagttg
8123-1 accttcacac ccacaacaca actatcactc aatatgggat ttgccgtgcc tctatgactc gctactgtaa ttattgggat acgaaatcaa ccaacagttg
8123-2 accttcacac ccacaacaca actatcactc aatatgggat ttgccgtacc tctatgactc gctactgtaa ttattgggat acgaaatcaa ccaacagttg
8123-3 accttcacac ccacaacaca actatcactc aatatgggat ttgccgtacc tctatgactc gctactgtaa ttattgggat acgaaatcaa ccaacagttg
8123-5 accttcacac ccacaacaca actatcactc aatatgggat ttgccgtacc tctatgactc gctactgtaa ttattgggat acgaaatcaa ccaacagttg
8123-7 accttcacac acccaacaca actatcactc aatatgggat ttgccgtacc tctatgactc gctactgtaa ttattgggat acgaaatcaa ccaacagttg

       2222222222 2222222222 2222222222 2222222222 2222222222 2222222222 2222222222 2222222222 2222222222 2222222223
       0000000001 1111111112 2222222223 3333333334 4444444445 5555555556 6666666667 7777777778 8888888889 9999999990
       1234567890 1234567890 1234567890 1234567890 1234567890 1234567890 1234567890 1234567890 1234567890 1234567890
8123-4 ccctaggaca cctactacca gaaggaacac ccattccact gatcccagta ctaattatca tcgaaacaat tagcttattt atccgcccat tagccctggg
8123-6 ccctaggaca cctactacca gaaggaacac ccattccact gatcccagta ctaattatca tcgaaacaat tagcttattt atccgcccat tagccctggg
8123-1 ccctaggaca cctactacca gaaggaacac ccattccact gatcccagta ctaattatca tcgaaacaat tagcttattt atccgcccat tagccctggg
8123-2 ccctaggaca cctactacca gaaggaacac ccattccact gatcccagta ctaattatca tcgaaacaat tagcctattt atccgcccat tagccctggg
8123-3 ccctaggaca cctactacca gaaggaacac ccattccact gatcccagta ctaattatca tcgaaacaat tagcctattt atccgcccat tagccctggg
8123-5 ccctaggaca cctactacca gaaggaacac ccattccact gatcccagta ctaattatca tcgaaacaat tagcctattt atccgcccat tagccctggg
8123-7 ccctaggaca cctactacca gaaggaacac ccattccact gatcccagta ctaattatca tcgaaacaat tagcctattt atccgcccat tagccctggg

       3333333333 3333333333 3333333333 3333333333 3333333333 3333333333 3333333333 3333333333 3333333333 333333333
       0000000001 1111111112 2222222223 3333333334 4444444445 5555555556 6666666667 7777777778 8888888889 999999999
       1234567890 1234567890 1234567890 1234567890 1234567890 1234567890 1234567890 1234567890 1234567890 123456789
8123-4 agtccgacta acagccaatc taaccgcagg tcaccgtgtt aatccaactc atcgccacag ctgtatttgt tctcctacca ataataccaa cagtagcat
8123-6 agtccgacta acagccaatc taaccgcagg tcacc-tgtt aatccaactc atcgccacag ctgtatttgt tctcctacca ataataccaa cagtag---
8123-1 agtccgacta acagccaatc taaccgcagg tcacc-tgtt aatccaactc atcgccacag ctgtatttgt tctcctacca ataataccaa cagtagcat
8123-2 agtccgacta acagccaatc taaccgcagg tcacc-tgtt aatccaactc atcgccacag ctgtatttgt tctcctacca ataatgccaa cagtagc--
8123-3 agtccgacta acagccaatc taaccgcagg tcacc-tgtt aatccaactc atcgccacag ctgtatttgt tctcctacca ataatgccaa cagtagc--
8123-5 agtccgacta acagccaatc taaccgcagg tcacc-tgtt aatccaactc atcgccacag ctgtatttgt tctcctacca ataatgccaa cagtagcat
```

8123-7 agtccgacta acagccaatc taaccgcagg tcacc-tgtt aatccaactc atcgccacag ctgtatttgt tctcctacca ataataccaa cagtagcat

SNP LOCATIONS: 148A/G, 275C/T, 386A/G

46.

```
                1 1111111112 2222222223 3333333334 4444444445 5555555556 6666666667 7777777778 8888888889 9999999990
       1234567890 1234567890 1234567890 1234567890 1234567890 1234567890 1234567890 1234567890 1234567890 1234567890
8142-2 ---------- ---------- ---------- ---------- ---------- ---------- ---------- ---------- ---------- ----------
8142-3 ggggaaaacc gctggccctg gaactgcagc tggccattta ccaacccctc ctggcccatg acggtacacc ctgagcccag gtactccttt gcatccacct
8142-5 ggggaaaact gctggccctg gaactgcagc tggccattta ccaacccctc ctggcccatg acggtacacc ctgagcccag gtactccttt gcatccacct
8142-4 ggggaaaact gctggccctg gaactgcagc tggccattta ccaacccctc ctggcccatg acggtacacc ctgagcccag gtactccttt gcatccacct
8142-7 ggggaaaact gctggccctg gaactgcagc tggccattta ccaacccctc ctggcccatg acggtacacc ctgagcccag gtactccttt gcatccacct
8142-8 ggggaaaact gctggccctg gaactgcagc tggccattta ccaacccctc ctggcccatg acggtacacc ctgagcccag gtactccttt gcatccacct
8142-6 ggggaaaact gctggccctg gaactgcagc tggccattta ccaacccctc ctggcccatg acggtacacc ctgagcccag gtactccttt gcatccacct
8142-1 ---------- ---------- ---------- ---------- ---------- ---------- ---------- ---------- ---------- ----------

       1111111111 1111111111 1111111111 1111111111 1111111111 1111111111 1111111111 1111111111 1111111111 1111111112
       0000000001 1111111112 2222222223 3333333334 4444444445 5555555556 6666666667 7777777778 8888888889 9999999990
       1234567890 1234567890 1234567890 1234567890 1234567890 1234567890 1234567890 1234567890 1234567890 1234567890
8142-2 ---------- ---------- ---------- ---------- ---------- ---------- -agggagtgg gaacttgaag gctgagtagg tatactttgc
8142-3 cctgcttgat gtgaggcatc ccagtaaagc tgcccttgcg accatcctgc atgctggggt gagggagtgg gaacttgaag gctgagtagg tatactttgc
8142-5 cctgcttgat gtgaggcatc ccagtaaagc tgcccttgcg accatcctgc atgctggggt gagggagtgg gaacttgaag gctgagtagg tatactttgc
8142-4 cctgcttgat gtgaggcatc ccagtaaagc tgcccttgcg accatcctgc atgctggggt gagggagtgg gaacttgaag gctgagtagg tatactttgc
8142-7 cctgcttgat gtgaggcatc ccagtaaagc tgcccttgcg accatcctgc atgctggggt gagggagtgg gaacttgaag gctgagtagg tatactttgc
8142-8 cctgcttgat gtgaggcatc ccagtaaagc tgcccttgcg accatcctgc atgctggggt gagggagtgg gaacttgaag gctgagtagg tatactttgc
8142-6 cctgcttgat gtgaggcatc ccagtaaagc tgcccttgcg accatcctgc atgctggggt gagggagtgg gaacttgaag gctgagtagg tatactttgc
8142-1 ---------- ---------- ---------- ---------- ---------- ---------- -agggagtgg gaacttgaag gctgagttgg tgtactttcc

       2222222222 2222222222 2222222222 2222222222 2222222222 2222222222 2222222222 2222222222 2222222222 2222222223
       0000000001 1111111112 2222222223 3333333334 4444444445 5555555556 6666666667 7777777778 8888888889 9999999990
```

```
       1234567890 1234567890 1234567890 1234567890 1234567890 1234567890 1234567890 1234567890 1234567890 1234567890
8142-2 gccggtgctt tcgaggagct gctttagctc ggacactggg tcgctaccgt cttgggatgg gttaacaaaa cgaggctggg aatcagtgtt caccgtcatc
8142-3 gccggtgctt tcgaggagct gctttagctc ggacactggg tcgctaccgt cttgggatgg gttaacaaaa cgaggctggg aatcagtgtt caccgtcatc
8142-5 gccggtgctt tcgaggagct gctttagctc ggacactggg tcgctaccgt cttgggatgg gttaacaaaa cgaggctggg aatcagtgtt caccgtcatc
8142-4 gccggtgttt tcgaggagct gctttagctc ggacactggg tcgctaccgt cttgggatgg gttaacaaaa cgaggctggg aatcagtgtt caccgtcatc
8142-7 gccggtgctt tcgaggagct gctttagctc ggacactggg tcgctactgt cttgggatgg gttaacaaaa cgaggctggg aatcagtgtt caccgtcatc
8142-8 gccggtgctt tcgaggagct gctttagctc ggacactggg tcgctactgt cttgggatgg gttaacaaaa cgaggctggg aatcagtgtt caccgtcatc
8142-6 gccggtgctt tcgaggagct gctttagctc ggacactggg tcgctactgt cttgggatgg gttaacaaaa cgaggctggg aatcagtgtt caccgtcatc
8142-1 actggcgtct ccaaggagtt gctttagctc agacactggg tcactactgc ctttggatgg gtttacaaaa caaagctggg aatcagcgtt ca------tc

       3333333333 3333333333 3333333333 3333333333 3333333333 3333333333 3333333333 3333333333 3333333333 3333333334
       0000000001 1111111112 2222222223 3333333334 4444444445 5555555556 6666666667 7777777778 8888888889 9999999990
       1234567890 1234567890 1234567890 1234567890 1234567890 1234567890 1234567890 1234567890 1234567890 1234567890
8142-2 catgggttcc gaggggagct tttctggcag cttccatctg tacccgggtt ccactgctgg ggtcggagat tgggggtcga tgggtgtatg cttgcaaatg
8142-3 catgggttcc gaggggagct tttctggcag cttccatctg tacccgggtt ccactgctgg ggtcggagat tgggggtcga tgggtgtatg cttgcaaatg
8142-5 catgggttcc gaggggagct tttctggcag cttccatctg tacccgggtt ccactgctgg ggtcggagat tgggggtcga tgggtgtatg cttgcaaatg
8142-4 catgggttcc gaggggagct tttctggcag cttccatctg tacccgggtt ccactgctgg ggtcggagat tgggggtcga tgggtgtatg cttgcaaatg
8142-7 catgggttcc gaggggagct tttctcgcag cttccacctg tacctgggtt ccactgctgg ggtcggagat tcggggtcga tgggtgtata cttgcaaatg
8142-8 catgggttcc gaggggagct tttctcgcag cttccacctg tacctgggtt ccactgctgg ggtcggagat tcggggtcga tgggtgtata ctcgcaaatg
8142-6 catgggttcc gaggggagct tttctcgcag cttccacctg tacctgggtt ccactgctgg ggtcggagat tcggggtcga tgggtgtata cttgcaaatg
8142-1 catgggttcc gaggggagct tttctcgcag cttccagctg tgccctggtt ccactgctgg agtcggggat taggggttgg tgggtgtatg cttgtaaatg

       4444444444 4444444444 4444444444 4444444444 4444444444 4444444444 4444444444 4444444444 4444444444 4444444445
       0000000001 1111111112 2222222223 3333333334 4444444445 5555555556 6666666667 7777777778 8888888889 9999999990
       1234567890 1234567890 1234567890 1234567890 1234567890 1234567890 1234567890 1234567890 1234567890 1234567890
8142-2 gggacttgga ggatgggtat gtggaagcat agtgagatgg gctagataaa atgtttggtg caggggcctc accctgagac tgaggtctct gctgatccca
8142-3 gggacttgga ggatgggtat gtggaagcat agtgagatgg gctagataaa atgtttggtg caggggcctc accctgagac tgaggtctct gctgatccca
8142-5 gggacttgga ggatgggtat gtggaagcat agtgagatgg gctagataaa atgtttggtg caggggcctc accctgagac tgaggtctct gctgatccca
8142-4 gggacttgga ggatgggtat gtggaagcat agtgagatgg gctagataaa atgtttggtg caggggcctc accctgagac tgaggtctct gctgatccca
8142-7 gggacttgga ggatgggtat gtggaagcat agtgagatgg gctagataaa atgtttggtg caggggcctc accctgaggc tgaggtctct gctgatccca
```

```
8142-8 gggacttgga ggatgggtat gtggaagcat agtgagatgg gctagataaa atgtttggtg caggggcctc accctgagac tgaggtctct gctgatccca
8142-6 gggacttgga ggatgggtat gtggaagcat agtgagatgg gctagataaa atgtttggtg caggggcctc accctgagac tgaggtctct gctgatccca
8142-1 gggacttgga ggatgggtat gtggaagcat agttagatgg gctagacaaa atgttaggtg caggggcctc accctgagac tgaggtctct gctgatccca

       5555555555 5555555555 5555555555 5555555555 5555555555 5555555555 5555555555 5555555555 5555555555 5555555556
       0000000001 1111111112 2222222223 3333333334 4444444445 5555555556 6666666667 7777777778 8888888889 9999999990
       1234567890 1234567890 1234567890 1234567890 1234567890 1234567890 1234567890 1234567890 1234567890 1234567890
8142-2 tgacagctcc ttttgaggat aatgtcctag agggatggac tgtggcatag cagagg---- -----aggac aaaagaggac caggtgatga ----------
8142-3 tgacagctcc ttttgaggat aatgtcctag agggatggac tgtggcatag cagagg---- -----aggac aaaagaggac caggtgatga agagaccact
8142-5 tgacagctcc ttttgaggat aatgtcctgg agggatggac tgtggcatag cagagg---- -----aggac aaaagaggac caggtgatga agagaccact
8142-4 tgacagctcc ttttgaggat aatgtcctag agggatggac tgtggcatag cagagg---- -----aggac aaaagaggac caggtgatga agagaccact
8142-7 tgacagctcc ttttgaggat aatgtcccag agggatggac tgtggcatag cagagg---- -----aggac aaaagaggac caggtgatga agagaccact
8142-8 tgacggctcc ttttgaggat aatgtcccag agggatggac tgtggcatag cagagg---- -----aggac aaaagaggac caggtgatga agagaccact
8142-6 tgacagctcc ttttgaggat aatgtcccag agggatggac tgtggcatag cagagg---- -----aggac aaaagaggac caggtgatga agagaccact
8142-1 tgaaatctcc tgttgagggt aatgacctgg agggatggac tgtggcatag cagaggtggc agaggaggac gaaagaggac caggtgatga ----------

       6666666666 6666666666 6666666666 6666666666 6666666666 6666666666 6666666666 6666666666 6666666666 6666666667
       0000000001 1111111112 2222222223 3333333334 4444444445 5555555556 6666666667 7777777778 8888888889 9999999990
       1234567890 1234567890 1234567890 1234567890 1234567890 1234567890 1234567890 1234567890 1234567890 1234567890
8142-2 ---------- ---------- ---------- ---------- ---------- ---------- ---------- ---------- ---------- ----------
8142-3 tgtggcatac tctgcgggt- ---------- ---------- ---------- ---------- ---------- ---------- ---------- ----------
8142-5 tgtggcatac tctgcgggta ggaggttggc agatgctgat ggtgatttgc aggtgaagat gcgcctggag aa-caacagt ttgtgggtca gtggcagaca
8142-4 tgtggcatac tctgcgggta ggaggttggc agatgctgat ggtgatttgc aggtgaagat gcgcctggag aaacaacagt ttgtgggtca gtggca----
8142-7 tgtggcatac tctgcgggta ggaggttggc agatgctgat ggtgatttgc aggtgaagat gcgcct---- ---------- ---------- ----------
8142-8 tgtggcatac tctgcgggta ggaggttgg- ---------- ---------- ---------- ---------- ---------- ---------- ----------
8142-6 tgtggcatac tctgcgggta ggaggttggc agatgctgat ggtgatttgc aggtgaagat gc-------- ---------- ---------- ----------
8142-1 ---------- ---------- ---------- ---------- ---------- ---------- ---------- ---------- ---------- ----------

       77
       00
```

```
       12
8142-2 --
8142-3 --
8142-5 ac
8142-4 --
8142-7 --
8142-8 --
8142-6 --
8142-1  -
```

SNP LOCATIONS: 208C/T, 248C/T, 327C/G, 337T/C, 345C/T, 372G/C, 390G/A, 528C/T, 529A/G

47.

```
                 1 1111111112 2222222223 3333333334 4444444445 5555555556 6666666667 7777777778 8888888889 9999999990
        1234567890 1234567890 1234567890 1234567890 1234567890 1234567890 1234567890 1234567890 1234567890 1234567890
8184-1  gctcaaccca ataacccgaa acgagcacat ttacagcgct tacaatatct ccgctgac-- ----acacag gacaacacca ccgagcgttt cctcaaagcg
8184-2  gctcaaccca ataacccgaa acgagcacat ttacagcgct tacaatatct ccgctgac-- ----acacag gacaacacca ccgagcgttt cctcaaagcg
8184-5  gctcaaccca ataacccgaa acgagcacat ttacagcgct tacaatatcc ccgctgacac aaggacacag gacaacacca ccgagcgttt cctcaaagcg
8184-6  gctcaaccca ataacccgaa acgagcacat ttacagcgct tacaatatcc ccgctgacac aaggacacag gacaacacca ccgagcgttt cctcaaagcg
8184-3  gctcaaccca ataacccgaa acgagcacat ttacagcgct tacaatatcc ccgctgacac aaggacacag gacaacacca ccgagcgttt cctcaaagcg
88184-7 gctcaaccca ataacccgaa acgagcacat ttacagcgct tacaatatcc ccgctgacac aaggacacag gacaacacca ccgagcgttt cctcaaagcg
8184-4  gctcaaccca ataacccgaa acgagcacat ttacagcgct tacaatatcc ccgctgacac aaggacacag gacaacacca ccgagcgttt cctcaaagcg
8184-8  gctcaaccca ataacccgaa acgagcacat ttacagcgct tacaatatcc ctgctgacac aaggacacag gacaacacca ccgagcgttt cctaaaagcg

        1111111111 1111111111 1111111111 1111111111 1111111111 1111111111 1111111111 1111111111 1111111111 1111111112
        0000000001 1111111112 2222222223 3333333334 4444444445 5555555556 6666666667 7777777778 8888888889 9999999990
        1234567890 1234567890 1234567890 1234567890 1234567890 1234567890 1234567890 1234567890 1234567890 1234567890
8184-1  ttcaaatgtt acgaaaacga gtcgtcacct ttcagtgaca cctgacacac cgtacacgga gcgcgcggtt tgtaaacatg cacgaattga cgatttcatt
8184-2  ttcaaatgtt acgaaaacga gtcgtcacct ttcagtgaca cctgacacac cgtacacgga gcgcgcggtt tgtaaacatg cacgaattga cgatttcatt
8184-5  ttcaaatgtt acgaaaacga gtcgtcacct ttcagtgaca cctgacacac cgtacacgga gcgcgcggtt tgtaaacatg cacgaattga cgatttcatt
```

```
8184-6  ttcaaatgtt acgaaaacga gtcgtcacct ttcagtgaca cctgacacac cgtacacgga gcgcgcggtt tgtaaacatg cacgaattga cgatttcatt
8184-3  ttcaaatgtt acgaaaacga gtcgtcacct ttcagtgaca cctgacacac cgtacacgga gcgcgcgatt tgtaaacatg cacgaattga cgatttcatt
88184-7 ttcaaatgtt acgaaaacga gtcgtcacct ttcagtgaca cctgacacac cgtacacgga gcgcgcggtt tgtaaacatg cacgaattga cgatttcatt
8184-4  ttcaaatgtt acgaaaacga gtcgtcacct ttcagtgaca cctgacacac cgtacacgga gcgcgcggtt tgtaaacatg cacgaattga cgatttcatt
8184-8  ttcaaatgtt acgaaaacga gtcgtcacct ttcagtgaca cctgacacac cgtacacgga gcgcgcggtt tgtaaacatg cacgaattga cgatttcatt

        2222222222 2222222222 2222222222 2222222222 2222222222 2222222222 2222222222 2222222222 2222222222 2222222223
        0000000001 1111111112 2222222223 3333333334 4444444445 5555555556 6666666667 7777777778 8888888889 9999999990
        1234567890 1234567890 1234567890 1234567890 1234567890 1234567890 1234567890 1234567890 1234567890 1234567890
8184-1  tatgatacga aaattattct tcaccggcac gtgctatatt ttgaattaaa aagaatacat caatgcaccg catccttaac cccatccttg tcctgaaatc
8184-2  tatgatacga aaattattct tcaccggcac gtgctatatt ttgaattaaa aagaatacat caatgcaccg catccttaac cccatccttg tcctgaaatc
8184-5  tatgatacga aaattattct tcatcggcac gtgctatatt tttaattaaa aagaatacat caatgcaccg catccttaac cccatccttg tcttgaaatc
8184-6  tatgatacga aaattattct tcatcggcac gtgctatatt tttaattaaa aagaatacat caatgcaccg catccttaac cccatccttg tcttgaaatc
8184-3  tatgatacga aaattattct tcatcggcac gtgctatatt ttgaattaaa aagaatacat caatgcaccg catccttaac cccatccttg tcttgaaatc
88184-7 tatgatacga aaattattct tcatcggcac gtgctatatt ttgaattaaa aagaatacat caatgcaccg catccttaac cccatccttg tcttgaaatc
8184-4  tatgatacga aaattattct tcatcggcac gtgctatatt ttgaattaaa aagaatacat caatgcaccg catccttaac cccatccttg tcttgaaatc
8184-8  tatgatgcga aaatgattct tcaccggcac gtgctatatt ttgaattaaa aagaatacat caatgcaccg catccttaac cccatccttg tcctgaaatc

        3333333333 3333333333 3333333333 3333333333 3333333333 3333333333 3333333333 3333333333 3333333333 3333333334
        0000000001 1111111112 2222222223 3333333334 4444444445 5555555556 6666666667 7777777778 8888888889 9999999990
        1234567890 1234567890 1234567890 1234567890 1234567890 1234567890 1234567890 1234567890 1234567890 1234567890
8184-1  tatattccag tgaaagtatc cgtgtttcga agcaggcttt tgaagcgtta tattgtaatt cactaatcta cgtgtaat-- ------aaat aaacactttg
8184-2  tatattccag tgaaagtatc cgtgtttcga agcaggcttt tgaagcgtta tattgtaatt cactaatcta cgtgtaat-- ------aaat aaacactttg
8184-5  tctattccag tgaaagtatc cgtgtttcga agcaggcttt tgaagcgtta tattgtaatt cactaatcta cgtgtaatgt atctataaat aaacactttg
8184-6  tctattccag tgaaagtatc cgtgtttcga agcaggcttt tgaagcgtta tattgtaatt cactaatcta cgtgtaatgt atctataaat aaacactttg
8184-3  tctattccag tgaaagtatc cgtgtttcga agcaggcttt tgaagcgtta tattgtaatt cactaatcta cgtgtaatgt atctataaat aaacactttg
88184-7 tctattccag tgaaagtatc cgtgtttcga agcaggcttt tgaagcgtta tattgtaatt cactaatcta cgtgtaatgt atctataaat aaacactttg
8184-4  tctattccag tgaaagtatc cgtgtttcga agcaggcttt tgaagcgtta tattgtaatt cactaatcta cgtgtaatgt atctataaat aaacactttg
8184-8  tatattccag tgaaagt-tc cgtgtttcaa agcacgcttt tgaagcgtta tattgtgatt cactaatata cgtgtaatgt atctataaat aaacactttg
```

```
        4444444444 444
        0000000001 111
        1234567890 123
8184-1  cggttgcctg ctt
8184-2  cggttgcctg ctt
8184-5  cggttgcctg ctt
8184-6  cggttgcctg ctt
8184-3  cggttgcctg ctt
88184-7 cggttgcctg ctt
8184-4  cggttgcctg ctt
8184-8  cggttgcctg ctt
```

SNP LOCATIONS: 50C/T, 224T/C, 243G/T, 293T/C, 302C/A

Table S1. Information of primers used in this study.

	Primers	Size(bp)	*T*m(°C)
7873-1	F: GTGGCAACAGTGCTAAAGT	180	51
	R: TCCATCACAAGAGTAACACG		
7873-2	F: TCCTTCTAATGTCCCCAACG	190	53
	R: CCCTGAACATCCTGAACCG		
7877	F: TAACCCCACCAGCAAGGCAG	163	56
	R: CACTCCAGGGAACACATAAGCGT		
7885	F: GTATGCTGCCCGCCAACT	215	51
	R: ATGCCACAACCACAATAGACTT		
7889	F: TGGCAAGCCTCTGGATGTAG	319	53
	R: CACAAAACTGTCTTCACTTCTGGT		
7892	F: TGGAGGAAGAAGCAGTCGG	253	58
	R: CAAACTTGATCTGGTTGACGC		
7895-2	F: TCCCCGTTCTCATGGACATC	157	55
	R: GGGTTGACACGCTGGTTC		
7896	F: TCAGCATCATTACTCCAGTC	448	49
	R: GTCACCAACAGGTCAAGC		
7898	F: TGGTGGTGTGTTCGACATCT	238	52
	R: GGGTGGAGTGTCTCTTGGAA		
7909	F: TCGCTTTAGTCTCCACCAT	533	49
	R: CCGAGACCACCTCTTCAG		
7918	F: CAAGAAGAAGATTGAGCACC	278	51
	R: TGACAGACTCCAGACAGCC		
7921	F: CTGGCTTCTGCTCACGCT	164	53
	R: CTGGTTTGCTTGGAGTTTCAC		
7927	F: AAGCCGTGGGACAAATCT	322	52
	R: AACAGTCATACAACACTTCAGG		
7929	F: ACCAGGCTCTTCATGTCAACACT	222	52
	R: CTTTATGCTTGCCGTCCTCTATC		
7933	F: TGCCTTTGGTGGAAATAGAC	429	52
	R: GCTGGAGGAACTTGCTGAT		
7943	F: GCAGCACACGCTTTATCAGT	402	49
	R: CTATCACGGCGAAAGTCATC		
7944	F: TGGTGACACTGGCTCTGGGT	298	56
	R: GGATGCTGTGATGGAGGAAAC		
7947	F: GGGTGGAGAAATACAAGAAC	236	52
	R: GCCAATGAGTATCTCTGTAGC		
7953	F: TGCCAGAGTATCAGTCCAAG	347	52
	R: CGTGTTGATTTTATCCAGTG		
7955	F: TAAAAGAACACAACGGGCAC	240	53
	R: CAGACTCAAAGTAGCAAACGG		

Table S1. *Cont.*

	**Primers**	**Size(bp)**	***T*m(°C)**
7957	F: CTTCCACGAGACCCTTTAC	354	53
	R: GTCCCCATCAGGTAGAATC		
7961	F: ACTTTCCAGAAGACGAGCAC	222	50
	R: CTGATAGACCTTATACCCAGC		
7969	F: TGGATTTAGAGAACGAAACAG	217	49
	R: TAACTTTGCTGGTTTGCTTC		
7974	F: GAGCGTCCATCAGAAAGAAT	215	50
	R: CAGTTTGTTGCTGGTGTCC		
7980-2	F: GTTGTCATCCCTGCTGGTGTCC	211	56
	R: CTCTGTTGGGGTTGTAGACGCC		
7986-2	F: TGAAAGCATTTGTTGAAGCG	191	53
	R: CTGTCTGATAGAAGCCACCTGT		
7988	F: CCCAGGAAGTATTGGTTAGAAG	184	52
	R: GTAGTGCGGTGGTTGGTATC		
7990	F: CATTACAGTCACCGACAGGG	273	53
	R: GTGCCAAATCATTCAGTTCC		
7992	F: GAACATCATAGTGCTGGGGA	139	52
	R: TGTATCTCTCTGTGAGGTCGTG		
8000	F: CCATTACAAAGATGCCCAAG	419	53
	R: AGAAGAGCGTCCAAAGTGAAC		
8001	F: ACCATCAGTGCCGCCCTCT	376	56
	R: CATCCGTCACAGCCTCCTCTC		
8008	F: GCTGAGCCAAGTGGGAGAT	233	55
	R: GTCGCCTCTGCTTTGTTTCT		
8009-2	F: AGGTGATGTTCCTGTCTGG	213	52
	R: ATGTACTTCCTGTTGCGTGT		
8011	F: TGGTTTGGAGGGCGGTTAGC	303	54
	R: GTCGGACCCCAGTCTTCGTATC		
8012	F: CAGAGTCCACGGCAAGTATGA	203	55
	R: GAAGTGTCCCCAACTAAGCAAG		
8013	F: ACACCTTCCCCCAGTAAACC	260	54
	R: GTGGCATCTTCTTGTTGAGTTG		
8017	F: CATTTCTGAGACCTTCAACCAT	313	54
	R: TTGTCATTTTCTGCTTGCTTC		
8021	F: TCTGTCACTTTAGATCCTGGTGTAG	447	53
	R: TGTTACCCACTAATCCTGTTCG		
8025	F: CAGGAACTTGTCCGTGTGTCT	318	54
	R: CAGGAAACCATTCAGTGCCA		
8034	F: CTGTGTTTGGTGGGTTTCG	327	54
	R: TTCATTCAGACGCCAGGTG		

Table S1. *Cont.*

	**Primers**	**Size(bp)**	***T*m(°C)**
8040	F: CCTCAGGAAGAAGGGCATC	348	55
	R: TGGTTTATCAGCGTACACAGC		
8041	F: CTTTCCTTCTACAAACCGTCTC	173	52
	R: TCCTCTGTCCTGATGGTGTCT		
8048	F: TAGGCAGTGAATGTGGGAT	256	52
	R: GGGAAGTAGGAGTAAGAGGTG		
8050-1	F: TCAAGGAAGGCAACGGGAAG	179	56
	R: CAGCGGGAGAAGTACCGAGTC		
8052cg2	F: ACCCTCATTGTGGTGTCC	286	54
	R: AGTTCTCGCTGTCTTTATCTG		
8123	F: ACCACTAAATGTAGGAGGAC	428	55
	R: GTAATGTGAGGAGGAAGAG		
8142	F: AGGGAGTGGGAACTTGAA	420	57
	R: TCATCACCTGGTCCTCTTT		
8184	F: GCTCAACCCAATAACCCGA	413	57
	R: AAGCAGGCAACCGCAAAG		
GT7892	F1: *ACTTA*CGATAAAGCCCGCAGA	160	
	F2: *ACGGTTGTAATAA*CGATAAAGCCCGCAGG	168	60
	R: GACGAAAGCACAGAGGTAAG		
GT7953	F1: *ACTTA*AACCAAAGAGCAGCTTCTCAG	149	
	F2: *ACGGTTGTAATAA*AACCAAAGAGCAGCTTCTCAC	157	62
	R: GCATATAGAGCACTGCCGAC		

© 2012 by the authors; licensee MDPI, Basel, Switzerland. This article is an open access article distributed under the terms and conditions of the Creative Commons Attribution license (http://creativecommons.org/licenses/by/3.0/).
